# Self-aligned patterning of tantalum oxide on Cu/SiO_2_ through redox-coupled inherently selective atomic layer deposition

**DOI:** 10.1038/s41467-023-40249-2

**Published:** 2023-07-26

**Authors:** Yicheng Li, Zilian Qi, Yuxiao Lan, Kun Cao, Yanwei Wen, Jingming Zhang, Eryan Gu, Junzhou Long, Jin Yan, Bin Shan, Rong Chen

**Affiliations:** 1 State Key Laboratory of Intelligent Manufacturing Equipment and Technology, School of Mechanical Science and Engineering, Huazhong University of Science and Technology, Wuhan, Hubei People’s Republic of China; 2 State Key Laboratory of Materials Processing and Die & Mould Technology, School of Materials Science and Engineering, Huazhong University of Science and Technology, Wuhan, Hubei People’s Republic of China; 3 Hubei Yangtze Memory Laboratories, Wuhan, Hubei People’s Republic of China

**Keywords:** Surface patterning, Surface patterning

## Abstract

Atomic-scale precision alignment is a bottleneck in the fabrication of next-generation nanoelectronics. In this study, a redox-coupled inherently selective atomic layer deposition (ALD) is introduced to tackle this challenge. The ‘reduction-adsorption-oxidation’ ALD cycles are designed by adding an in-situ reduction step, effectively inhibiting nucleation on copper. As a result, tantalum oxide exhibits selective deposition on various oxides, with no observable growth on Cu. Furthermore, the self-aligned TaO_x_ is successfully deposited on Cu/SiO_2_ nanopatterns, avoiding excessive mushroom growth at the edges or the emergence of undesired nucleation defects within the Cu region. The film thickness on SiO_2_ exceeds 5 nm with a selectivity of 100%, marking it as one of the highest reported to date. This method offers a streamlined and highly precise self-aligned manufacturing technique, which is advantageous for the future downscaling of integrated circuits.

## Abstract (web summary)

‘Atomic-scale precision alignment is a bottleneck in the fabrication of next generation nanoelectronics. Here, the authors used redox-coupled inherently selective atomic layer deposition of tantalum oxide on Cu/SiO_2_ and achieved 100% selective deposition on SiO_2_ and no observable growth on copper.’

## Introduction

The semiconductor industry continues to develop smaller and better-performing nano-electronic devices; high-resolution patterning is a critical step in determining the manufacturability of such devices. Misalignment impedes high-precision patterning because nanodevices tend to shrink to the atomic scale^1–6^. Traditional top-down “deposition-lithography-etch” multiple manufacturing steps are limited by significant challenges such as control of edge-placement error (EPE) and complexity of the steps^7^. Moreover, new materials and 3D nanostructures have stringent requirements for high-volume manufacturing (HVM) technology^8–12^. Atomic layer deposition (ALD) is a powerful thin film manufacturing technology, wherein thin films are grown by self-limiting chemical reactions between the precursors and the substrate^13^. Moreover, selective ALD is a promising technique because it allows the atomic-scale precision alignment with simplified steps^14–19^. Selective ALD enables deposition of films only on the desired regions of pre-patterned substrates. This method promotes effective nanoelectronics manufacturing. For example, previous studies have adopted a selective dielectric-on-dielectric ALD process to create a dielectric scaffold that prevented the vias from getting too close to the neighboring metal features, leading to a more significant process margin for EPE during via formation^20,21^.

Selective ALD between dielectric oxides and metals was achieved through surface passivation. Polymers^22^, self-assembled monolayers (SAMs)^23–27^, and small molecule inhibitors^28,29^ have been used to block nucleation in non-growth areas. For example, SAMs have been used to block nucleation on Cu/Co regions to achieve fully self-aligned via (FSAV) integration. This approach demonstrated improvement of two orders of magnitude in the via-to-line time-dependent dielectric breakdown for interconnect scaling beyond the 3 nm node^30^. Moreover, the selective tungsten filling technology eliminated MOL/BEOL parasitic resistance caused by the liner/barrier and seed layer, resulting in reduced resistivity and improved circuit performance^31^. These inhibitors assisted selective ALD methods are close to practical industrial applications, but challenges still exist. This process requires a long immersion time and subsequent removal of SAMs or inhibitors. Moreover, severe limitations exist because the blocking effect of SAMs deteriorates during the deposition process and is heavily temperature-dependent^32^. Other inhibitors suffer from limited materials and process parameters required for reliable selectivity^33–35^. Thus, as the critical size decreases below 10 nm and the demand for new metallic and dielectric materials and 3D nanostructures continue to increase, the appropriate chemicals for passivation-assisted selective ALD become very important. Inherently selective ALD is a more straightforward method for alignment manufacturing, that is free of inhibitor passivation and removal steps^36–39^. In particular, ALD relies on intrinsic surface properties differences. For example, inherently selective deposition process has been reported for oxides deposited on noble metals such as Ru, Pt. The selectivity originates from the catalytic combustion of the precursor ligands and preferential dissociation of co-reactants on metals. Thus, the target film is deposited on the metal, but not the dielectric^40,41^. In contrast, selective deposition of dielectric on dielectric is challenging to suppress nucleation on metals while ensuring deposition on dielectrics. This process is vital for FSAV fabrication becasue it increases the spacing between vias and metal lines. Hence, the use of reductive co-reactants to reduce metal catalytic activity is a promising method. Some chemicals, such as acetic acid^42^, ethanol^43–45^, isopropyl alcohol^46^, and tert-butylamine^47^, are used to prevent surface oxidation and undesired nucleation on the metal. Nonetheless, nucleation on metal is still difficult to suppress^48^. Inherently selective ALD is very sensitive to surface chemistry, and adjustment of the metal surface is an essential factor for selective ALD.

Herein, a redox-coupled inherently selective ALD is developed that effectively inhibits nucleation on Cu. Tantalum oxide exhibits selective deposition on various oxides, with no observable growth on copper. TaO_x_ films are widely used as insulating layer for nanoelectronics, functional layer for memory devices, etc^49–52^. This study reveals that the loss of selectivity is attributed to the surface oxidation of Cu, and proposes the “reduction-adsorption-oxidation” ALD cycles. The optimized selectivity of 100% is achieved, and the maximum thickness of the film deposited on SiO_2_is 5–6 nm. This work is one of the highest reported to date. During the redox-coupled ALD process, the EtOH pulse before each binary ALD cycle can reduce surface oxidation in situ and suppress undesired nucleation on Cu. The selectivity originates from the higher energy barrier during ALD nucleation on reduced Cu than that of OH-terminated SiO_2_. Finally, the selective deposition approach is transferred for self-alignment on nanoscale Cu/SiO_2_ patterns, and excessive ‘mushroom’ growth at the edges and formation of nucleation defects on the Cu region are not observed. The ALD method provides a streamlined bottom-up avenue for self-alignment nanomanufacturing and unfolds possibilities for semiconductor applications.

## Results and discussion

### AB and ABC-type ALD processes

**Fig. 1:**
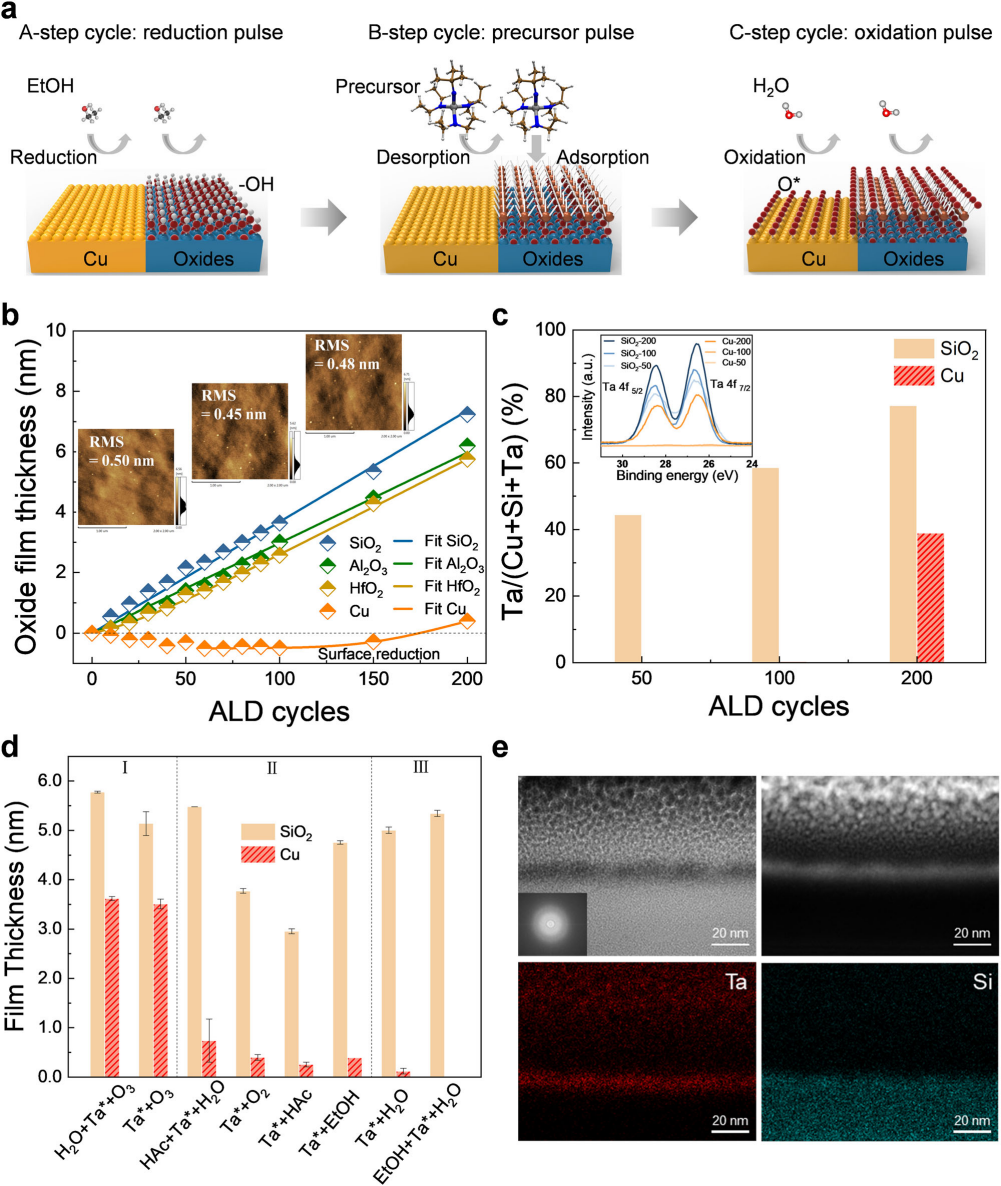
The selective ALD processes. **a** The scheme of redox-coupled ABC-type ALD. **b** The TaO_x_ film thickness as a function of ALD cycles, the line curves are fitting data through the nucleation model, and the insets show the AFM images and the root-mean-square (RMS) roughness of the films corresponding to 50, 100, and 150 ALD cycles. **c** The proportion of Ta element on Cu and SiO_2_ during 50,100, and 200 ALD cycles, respectively, the inset shows the corresponding high-resolution XPS scan of Ta 4 f. **d** Film thickness versus different AB-type and ABC-type ALD processes between Cu and SiO_2_. Each data point of the film thickness is measured at two positions of one sample by spectroscopic ellipsometry. Error bars represent standard deviations after two measurements of each sample. **e** The cross-sectional bright-field and dark-field TEM images, and element scans of Ta and Si of the TaO_x_ thin film, the insetting electron diffraction image shows the film is amorphous.

Traditional binary ALD was optimized by incorporating an in-situ reduction step into each binary AB-type ALD cycle. The ‘reduction-adsorption-oxidation’ ALD cycles were designed, and the scheme of this redox-coupled ABC-type (co-reactant A $\to$ precursor B $\to$ co-reactant C) ALD is presented in Fig. 1a. An additional reduction pulse continuously mitigated the oxidation of Cu regions, which further improved the selectivity between Cu and oxides (including SiO_2_, Al_2_O_3_, and HfO_2_). The in-situ reduction step has potential to be integrated into the industrial process without the requirement of long-term liquid passivation with SAMs and removal steps.

The deposition temperature is essential for selective ALD. First, the AB-type ALD process with Ta(N^t^Bu)(NEt_2_)_3_-H_2_O as precursors was conducted at 100, 200, and 300 °C. When the deposition temperature was decreased to 100 °C (Supplementary Fig. 1), the average deposition rate on SiO_2_ and Cu is 0.16 and 0.14 nm/cycle, respectively. The selectivity of the AB-type ALD process decreased significantly, which was probably attributed to the partial condensation of the precursor on the substrate. When the temperature was increased to 300 °C (Supplementary Fig. 2), the selectivity between Cu and SiO_2_ also deteriorated. This may be ascribed to the oxidation of Cu and the decomposition of the precursors^53^. At 200 °C (Supplementary Fig. 3), ALD initial growth rate on Cu was inhibited by using EtOH and H_2_O as co-reactant. The selectivity is quantified with the formula (θ_GA_-θ_NGA_)/(θ_GA_ + θ_NGA_), θ_GA_ is the thickness or amount of material deposited on the growth region, and θ_NGA_ is the thickness or amount of material on the non-growth. The selectivity of ALD process performed with Ta(NtBu)(NEt_2_)_3_-H_2_O was obtained 32%, 91%, and 88% at 100 °C, 200 °C, and 300 °C, respectively (Supplementary Fig. 4). The saturate time of Ta precursor on SiO_2_ was ~2 s. Decreasing the Ta precursor pulse time to 1 s, the nucleation delay on Cu could be maintained to 100 cycles while the growth rate on SiO_2_ was too slow. Increasing the pulse time to 3 s, the selectivity deteriorated due to quick nucleation on Cu. (Supplementary Fig. 7).

Then, the redox-coupled ABC-type ALD performed with EtOH-Ta(N^t^Bu)(NEt_2_)_3_-H_2_O as precursors at 200 °C was studied. An apparent nucleation delay of more than 150 cycles on the Cu surface was observed (Fig. 1b). It was found that the film thickness could be lower than zero when ethanol was utilized in the ALD process. This was reasonable as ethanol could reduce the native oxide of copper. To study it further, bare copper substrates were tested using SE measurements after exposure to ethanol, H_2_O, O_3_ pulses (Supplementary Fig. 8). The results showed that the decrease of surface oxide layer thickness with ethanol pretreatment was about 0.5 nm. H_2_O had minimal influence on the surface oxide layer, while O_3_ strongly oxidized the Cu surface, thereby increasing the surface oxide layer. The surface reduction process was also reported capable to improve the interface and film quality^54^. The surface morphology of the TaO_x_ films deposited on SiO_2_ was studied through atomic force microscopy (AFM) (insets in Fig. 1b). The roughness of the original substrates was evaluated through AFM in Supplementary Fig. 5. After the ALD process, the surface roughness values were 0.50, 0.45, and 0.48 nm after 50, 100, and 150 ABC-type ALD cycles, respectively. Barely particles were observed on the SiO_2_ and Cu surface (Supplementary Figs. 5 and 6), indicating that smooth film was obtained. For the ABC-type ALD, it was found that the growth rate of Ta_2_O_5_ on Al_2_O_3_ and HfO_2_ was lower than that on SiO_2_, which confirmed the occurrence of previously reported surface acidity-induced selective deposition^55^. A total of 10 s purge time was sufficient to remove excess precursors and by-products. High-precision X-ray photoelectron spectroscopy (XPS) was conducted to quantitatively compare the amount of TaO_x_ on Cu and SiO_2_. At 50 and 100 ALD cycles, peak ascribed to Ta 4 f was not observed, indicating barely growth of TaO_x_ on Cu (inset in Fig. 1c). The proportion of Ta was almost zero on the Cu surface at 50 cycles and 100 cycles indicating 100% selectivity. With increasing number of ALD cycles to 200, the peak intensity of Ta on the SiO_2_ and Cu substrates increased simultaneously. The Ta element ratios were 39% for Cu and 77% for SiO_2_ after 200 ALD cycles, resulting in loss of selectivity (Fig. 1c).

A nucleation model proposed by Parsons^18^ is adopted and varied to fit the selective ALD process, as indicated by the line curves in Fig. 1b. The nucleation model includes the factors of the normal ALD nucleation ($\dot{N}$ (nm^−2^)), defect induced nucleation ($\hat{N}$ (nm^−2^)), the anisotropic growth of the existed nucleus, and atomic diffusion induced nucleation in the dynamic expanding region at the edge of nucleus (${\dot{N}}^{{\prime} }$ (nm^−2^). The details of the nucleation model are described in Supplementary Materials (Supplementary Fig. 9). The fitting curves are consistent with the experimental results, and the proper error f_err_ is less than 2 × 10^−2^. For ABC-type ALD processes on SiO_2_ and Al_2_O_3_ substrates, the values of nucleation site density induced per ALD cycle on non-defect sites $\dot{N}$, defect-induced nucleation site density $\hat{N}$ are at the order of 10^−1 ^nm^−2^. This suggests that the rapid and linear growth observed on these surfaces is associated with the formation of a significant number of nucleation sites. The G_v_ value of TaO_x_ on Al_2_O_3_ is fitted to be about 62% that of SiO_2_ in Supplementary Fig. 10, which agrees with the experimental ratio 78%. (Supplementary Table 1) The values of $\dot{N}$ and ${\dot{N}}^{{\prime} }$ on the HfO_2_ substrate are on the order of 10^−3 ^nm^−2^, which is significantly lower than the values on the SiO_2_ and Al_2_O_3_ substrates, resulting in slow initial nucleation rate. The values of $\dot{N}$ and $\hat{N}$ on Cu are 2.9 × 10^−7 ^nm^−2^ and 1.1 × 10^−4 ^nm^−2^, respectively, which are much lower than those on oxide substrates (Supplementary Fig. 10). The results suggest that defect-induced nucleation is limited on Cu, and those parameters $\dot{N}$ and $\hat{N}$ are the critical factors for nucleation delay.

For selective ALD process optimization, a series of AB- and ABC-type ALD processes were conducted and compared in Fig. 1d. The reductant (EtOH), acid (HAc), and oxidants (H_2_O, O_2_, and O_3_) were studied as co-reactants. For the AB-type ALD, the growth rate on SiO_2_ with different co-reactants decrease as follows: H_2_O > O_3_ > O_2_ > HAc > EtOH (Supplementary Fig. 11). H_2_O provides more active hydroxyl sites on the surface and promotes the precursor adsorption. It is expected that the next half-reaction of ALD may be different for various co-reactants^56^. Except for the O_3_ process, an apparent nucleation delay on the Cu surface is observed. (Supplementary Fig. 3) The O_3_ co-reactant strongly oxidizes the Cu surface and offers active surface sites to initiate growth; thus, there’s no nucleation delay on the Cu surface. The EtOH as a co-reactant could achieve high selectivity; however, it is difficult to oxidize the Ta precursor; thus, the deposition rate is slow (Supplementary Fig. 3a, b). The film thickness is <5 nm after 650 ALD cycles, which is time-consuming and precursor-wasting. Although the H_2_O-based ALD process has high selectivity, the nucleation delay is quickly lost after 50 ALD cycles. Other Ta precursors with different coordinating groups were also exploited, including Ta(NMe_2_)_5_ and Ta(OEt)_5_. However, both precursors need highly-active O_3_ as co-reactant, thus the Cu substrate is strongly oxidized which is harmful to the selective ALD process (Supplementary Fig. 12). Different ABC-type ALD processes (co-reactant A $\to$ precursor B $\to$ co-reactant C) are developed, including EtOH-Ta(N^t^Bu)(NEt_2_)_3_-H_2_O, HAc-Ta(N^t^Bu)(NEt_2_)_3_-H_2_O, H_2_O-Ta(N^t^Bu)(NEt_2_)_3_-O_3_. The EtOH-Ta(N^t^Bu)(NEt_2_)_3_-H_2_O-type process exhibits the highest selectivity of 100% and can be maintained for 100 cycles (Supplementary Figs. 13, 14). During each ALD cycle, the co-reactant in the A-step is proposed to be utilized for in situ surface reduction, followed by two subsequent half-reactions, including the chemisorption of the precursor and the subsequent oxidation reaction with the co-reactants. To suppress nucleation in the non-growth Cu region, the EtOH-based A-step is preferred over other ABC-type processes. For the HAc-Ta(N^t^Bu)(NEt_2_)_3_-H_2_O ABC-type ALD process, acetic acid slightly etches Cu surface; and it provides more active sites for ALD nucleation that deteriorated the selectivity between Cu and SiO_2_.

The selective ALD processes of TaO_x_ on Cu/SiO_2_ could be divided into three groups (Fig. 1d). The O_3_-based process in region I is not appropriate for selective deposition, as it strongly oxidized the Cu surface. In region II, the EtOH-based AB-type binary process is also unsuitable because of its low growth rate. The selectivity of the H_2_O-based AB-type binary process is higher than that of the highly active O_3_ at the initial growth stage within 50 cycles, but decreases quickly. In region III, the EtOH-Ta(N^t^Bu)(NEt_2_)_3_-H_2_O ABC-type process is the most preferred one. It achieves the highest selectivity and the longest nucleation delay on the non-growth Cu region, both are critical for a reliable self-aligned oxide stacking. Nucleation on Cu is still inhibited when the film thickness is obtained ~5–6 nm on SiO_2_. Figure 1e shows the cross-section TEM images of TaO_x_ film on SiO_2_ after 150 ABC-type ALD cycles; the film is continuous and amorphous. GI-XRD results confirm the amorphous structure (Supplementary Fig. 15). For inherently selective ALD, there is a trade-off between selectivity and film thickness on SiO_2_. In this work, the selectivity between Cu and SiO_2_ is compared with those reported in previous studies. The obtained selectivity (100%) is the highest among those reported for other developed inherently selective ALD strategies. (Supplementary Fig. 16).

### Investigations of Cu surface chemical state to selective ALD

**Fig. 2:**
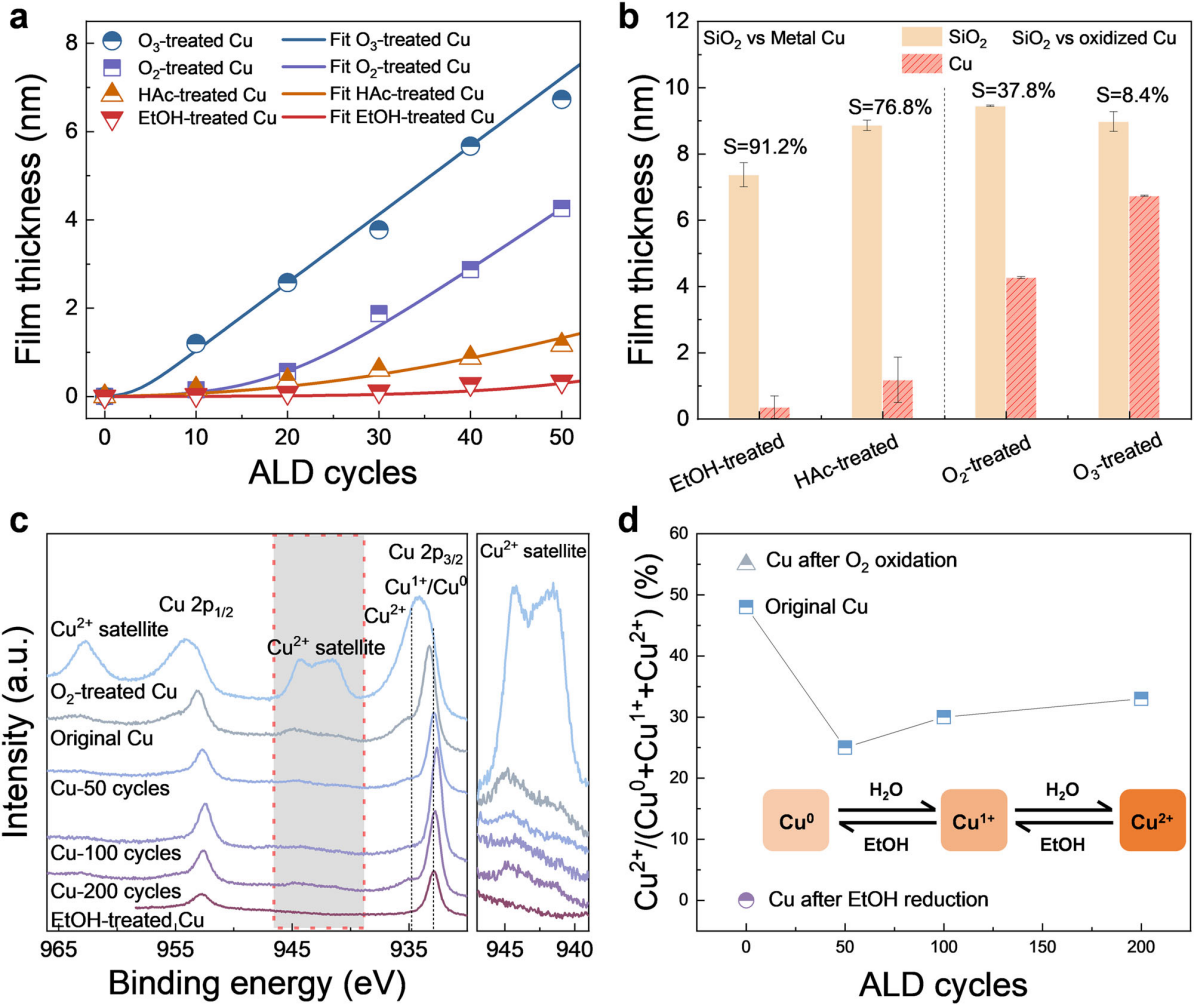
The effect of chemical state of Cu surface to selective ALD. **a** The film thickness *versus* ALD cycles for O_3_-treated, O_2_-treated, HAc-treated, and EtOH-treated Cu. **b** The film thickness and selectivity as a function of the ALD cycles on SiO_2_ and Cu substrates with different treatments. The selectivity is quickly lost for oxidized Cu. Each data point of the film thickness is measured at two positions of one sample by spectroscopic ellipsometry. Error bars represent standard deviations after two measurements of each sample. **c** The Cu *2p* scan of XPS and **d** the proportion of Cu^2+^ after O_2_ oxidation, EtOH reduction, and during 0, 50, 100, and 200 ALD cycles. The highlighted region on the left of **c** means the Cu^2+^ satellite of Cu *2p*, which is enlarged as the right of **c**. The insets in **d** show the competition between the removal of Cu oxides during EtOH dosing and the oxidation of the Cu surface during H_2_O dosing.

The nucleation delay is influenced by Cu surface chemical state. To investigate the origin of the selectivity loss, Cu surface with different pretreatments followed by AB-type ALD (Ta(N^t^Bu)(NEt_2_)_3_-H_2_O) process are conducted, including EtOH-treated Cu, HAc-treated Cu, O_2_-treated, and O_3_-treated Cu (Fig. 2a). EtOH-reduced Cu exhibits a high selectivity (91.2% during 50 ALD cycles). With HAc immersion pretreatment, the thickness of the Cu film decreases, indicating that the surface Cu oxides is slightly etched. HAc-etched Cu exhibits high selectivity (76.8% during 50 ALD cycles). With the oxidizing atmosphere treatment, the thickness of the Cu films increased, indicating that the surface is strongly oxidized to CuO. The selectivity for O_2_-oxidized and O_3_-oxidized Cu is decreased to 37.8% and 8.4%, respectively. For the O_2_-treated Cu surface, nucleation occurs quickly, and the growth rate is similar to that of the SiO_2_ substrate after 20 ALD cycles, indicating that surface oxidation decreases selectivity. The O_3_-treated Cu surface exhibits linear growth behavior similar to that of SiO_2_, which is also attributed to oxidation and activation with ozone. The growth curves on different Cu surfaces are fitted with the nucleation model, the key parameters are summarized in Supplementary Table 2. All fitting errors (f_err_) are less than 4 × 10^−2^. For EtOH-treated Cu, $\hat{N}$ is the lowest; thus, the nucleation delay is the longest. $\dot{N}$ and ${\dot{N}}^{{\prime} }$ increase roughly in the order of EtOH-treated Cu, HAc-treated Cu, O_2_-treated Cu, and O_3_-treated Cu. Thus, nucleation delays decreased in the same order.

The comparison of selectivity influenced by different pretreatments followed with AB-type ALD (Ta(N^t^Bu)(NEt_2_)_3_-H_2_O) process is summarized in Fig. 2b. The oxidation of Cu with O_2_ and O_3_ is unfavorable for initiating nucleation on Cu. The Cu *2p* XPS spectra are obtained (Fig. 2c). In the spectra, Cu^2+^ satellite peaks were observed for oxidized Cu, and the peaks located at 933.8 and 935.4 eV are ascribed to Cu^0^/Cu^1+^ and Cu^2+^, respectively (Supplementary Fig. 17). Except for oxidized Cu, other samples show a low-intensity Cu^2+^ satellite peak. The oxidation ratio of Cu during ABC-type ALD process is also presented in Fig. 2d. The original Cu surface has Cu^2+^ proportion of 48%. After 50 cycles with ABC-type ALD, the Cu^2+^ concentration decreases to 25%, indicating reduction of the Cu surface during EtOH pulses. As the number of ALD cycles increase, the Cu oxidation state slightly increase to 30% for 100 ALD cycles and 33% for 200 ALD cycles. In contrast, the ratio of Cu^2+^ could be decreased to zero after only EtOH pulses treatment, indicating the reduction of Cu by EtOH is very effective. Thus, during ABC-type ALD, the reduction during the EtOH pulse and oxidation during the Ta precursor and H_2_O pulses competitively occur on Cu films. Oxidation ultimately dominated and led to the nucleation of precursors on Cu.

The reduction of Cu oxides by EtOH is presented:

1 $${{{{{\mathrm{CuO}}}}}}+{{{{{\mathrm{C}}}}}}{{{{{\mathrm{H}}}}}}_{3}{{{{{\mathrm{C}}}}}}{{{{{\mathrm{H}}}}}}_{2}{{{{{\mathrm{OH}}}}}}\to {{{{{{\mathrm{Cu}}}}}}}_{2}{{{{{\mathrm{O}}}}}}+{{{{{\mathrm{H}}}}}}_{2} {{{{{\mathrm{O}}}}}} \left({{{{{\mathrm{g}}}}}}\right)+{{{{{\mathrm{C}}}}}}{{{{{\mathrm{H}}}}}}_{3}{{{{{\mathrm{CHO}}}}}}({{{{{\mathrm{g}}}}}})$$

2 $${{{{{{\rm{Cu}}}}}}}_{2}{{{{{\rm{O}}}}}}+{{{{{\rm{C}}}}}}{{{{{{\rm{H}}}}}}}_{3}{{{{{\rm{C}}}}}}{{{{{{\rm{H}}}}}}}_{2}{{{{{\rm{OH}}}}}}\to {{{{{\rm{Cu}}}}}}+{{{{{{\rm{H}}}}}}}_{2}{{{{{\rm{O}}}}}}\left({{{{{\rm{g}}}}}}\right)+{{{{{\rm{C}}}}}}{{{{{{\rm{H}}}}}}}_{3}{{{{{\rm{CHO}}}}}}({{{{{\rm{g}}}}}})$$

The etching of Cu oxides by HAc is presented:

3 $${{{{{\mathrm{CuO}}}}}}+{{{{{{\mathrm{CH}}}}}}}_{3}{{{{{\mathrm{COOH}}}}}}\to {{{{{\mathrm{Cu}}}}}}{({{{{{{\mathrm{CH}}}}}}}_{3}{{{{{\mathrm{COO}}}}}})}_{2}+{{{{{\mathrm{H}}}}}}_{2}{{{{{\mathrm{O}}}}}}({{{{{\mathrm{g}}}}}})$$

### The surface adsorption and reaction products analysis

**Fig. 3:**
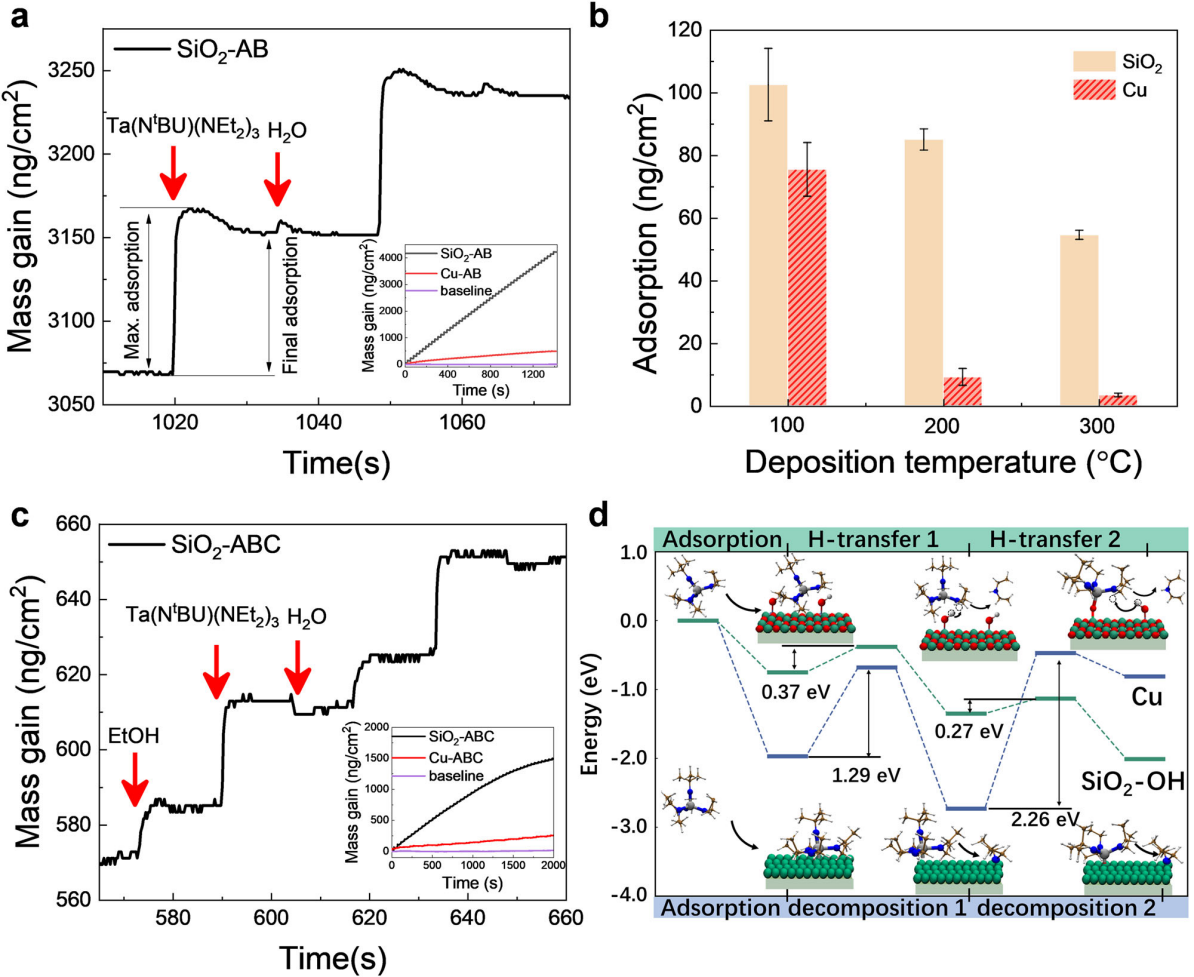
The surface adsorption characterizations of precursors on Cu and SiO2. **a** The mass gain as a function of deposition time during an AB-type (Ta(N^t^Bu)(NEt_2_)_3_-H_2_O) ALD cycle, the inset shows the total mass gain on SiO_2_ and Cu. **b** The mass gain during precursors’ pulse at the different growth temperatures. Error bars represent standard deviation of at least three measurements. **c** The mass gain as a function of deposition time during ABC-type (EtOH-Ta(N^t^Bu)(NEt_2_)_3_-H_2_O) ALD, the inset shows the total mass gain on SiO_2_ and Cu. **d** DFT calculations of reaction path for reduced Cu and OH-terminated SiO_2_.

An in-situ quartz crystal microbalance (QCM) was used to investigate the nucleation behavior. According to the Sauerbrey equation, the attenuation of the resonant frequency of the crystal oscillator was proportional to the minor mass change during deposition^57^. The mass gain during a single ALD cycle was presented (Fig. 3a). On the SiO_2_ surface, it was found to be much higher than that on Cu, according to the data in the insets of Fig. 3a. The mass gain on Cu and SiO_2_ during AB-type (Ta(N^t^Bu)(NEt_2_)_3_-H_2_O) ALD at (a) 100 °C and (b) 300 °C are also shown in Supplementary Fig. 18. The average mass gains of SiO_2_ and Cu after each precursor’s pulse are presented in Fig. 3b. Increasing the growth temperature promotes the desorption of ALD precursors; thus, the mass gain measured by the QCM decreases, which is consistent with the results of the ellipsometry tests. The adsorption of water is also influenced with deposition temperature, more H_2_O molecules are physically adsorbed at lower temperature, thus the initial mass gain is larger after dosing H_2_O at 100 °C. During ABC-type ALD, the adsorbed EtOH is found to hinder the subsequent adsorption of Ta precursors on SiO_2_ in Fig. 3c. EtOH is also capable to convert surface hydroxyl groups to ethoxide groups and act as an inhibitor to block adsorption sites. This is another reason to suppress nucleation on Cu surfaces (Supplementary Fig. 19). It should be noted that the growth rate on Cu surface with optimized ABC-type ALD is not suppressed to zero by QCM measurements, which may be a result of a highly rough Cu morphology deposited on crystal oscillator (Supplementary Fig. 20). For AB-type ALD reactions, HNEt_2_ and [HNEt_2_-CH_3_]^+^ can be detected with in-situ quadrupole mass spectrometer when the Ta precursors are dosed (Supplementary Fig. 21). The results indicate that the H-transfer reaction occurs between the hydroxyl groups and Ta precursors on SiO_2_. In addition, the partial pressure of the by-products during the precursor pulse in the ABC-type process is lower than that in the AB-type process, indicating that the EtOH pulses may hinder the chemisorption of the Ta precursor. To compare the composition and dielectric constant of TaO_x_ films fabricated by AB-type (Ta(N^t^Bu)(NEt_2_)_3_-H_2_O) and ABC-type (EtOH-Ta(N^t^Bu)(NEt_2_)_3_-H_2_O) ALD, XPS sputter depth profiles are analyzed. The results show that the ethanol used in the ABC-type ALD process do not influence the carbon concentration in the deposited film (Supplementary Fig. 22). The dielectric constants of the tantalum oxide film for AB and ABC ALD processes are 21.6 and 20.6, respectively. The k values are obtained through CV test and the values are similar (Supplementary Fig. 23).

### DFT simulations

To study the origin of the selectivity between Cu and SiO_2_, DFT calculations are employed. (Table S3, Fig. 3d) The Ta(N^t^Bu)(NEt_2_)_3_ precursor is thought to decompose the -NEt_2_ ligands on the reduced Cu surface. The reaction equation is shown as follows, where $*$ indicates empty sites, Ta(N^t^Bu)(NEt_2_)_x-1_^*^ is the remaining ligand on the surface, and NEt_2_^*^ is the ligand decomposes to the empty site:

4 $${{{{{\mathrm{Ta}}}}}}\left({{{{{\mathrm{N}}}}}}^{{{{{{\mathrm{t}}}}}}}{{{{{\mathrm{Bu}}}}}}\right){\left({{{{{\mathrm{NEt}}}}}}_{2}\right)}_{{{{{{\mathrm{x}}}}}}}^{*}+*\to {{{{{\mathrm{Ta}}}}}}\left({{{{{\mathrm{N}}}}}}^{t}{{{{{\mathrm{Bu}}}}}}\right){\left({{{{{\mathrm{NEt}}}}}}_{2}\right)}_{{{{{{\rm{x}}}}}}-1}^{*}+{{{{{\mathrm{NEt}}}}}}_{2}^{*}\left({{{{{\rm{x}}}}}} \ge 1\right)$$

The decomposition of the first NEt_2_^*^ ligand is difficult because of its high reaction barrier (*E*_*b*_ = 1.29 eV). In addition, the second -NEt_2_ decomposition is hindered, as verified by thermodynamics ($\varDelta H=1.92$ eV) and kinetically (*E*_*b*_ = 2.26 eV). On the OH-terminated SiO_2_ surface, H-transfer from the hydroxyl on the surface to the Ta(N^t^Bu)(NEt_2_)_3_ precursor occurrs. The H-transfer reaction is revealed to be a key factor for the inherently selective ALD on oxide substrates^55^. When the precursor reacts on the surface, it is assumed that some -NEt_2_ and -N^t^Bu groups are released through ligand exchange reactions with the OH surface groups. The ratio between the two possible reaction by-products, HNEt_2_(g) and H_2_N^t^Bu(g), are reported to be 1.7:0.3^58^. Then, a subsequent H_2_O pulse transferred the remaining ligands and transformed the surface back to a hydroxyl. The reaction equations are as follows:

H-transfer-1:

5 $${{{{{\mathrm{Ta}}}}}}\left({{{{{\mathrm{N}}}}}}^{{{{{\mathrm{t}}}}}}{{{{{\mathrm{Bu}}}}}}\right){\left({{{{{{\mathrm{NEt}}}}}}}_{2}\right)}_{3}^{*}+{{{{{{\mathrm{OH}}}}}}}^{*}\to {{{{{\mathrm{Ta}}}}}}\left({{{{{\mathrm{N}}}}}}^{{{{{\mathrm{t}}}}}}{{{{{\mathrm{Bu}}}}}}\right){\left({{{{{{\mathrm{NEt}}}}}}}_{2}\right)}_{2}^{*} \sim {{{{{\mathrm{O}}}}}}+{{{{{\mathrm{HNE}}}}}}{{{{{\mathrm{t}}}}}}_{2}({{{{{\mathrm{g}}}}}})$$

6 $${{{{{\mathrm{Ta}}}}}} \left({{{{{\mathrm{N}}}}}}^{{{{{{\mathrm{t}}}}}}}{{{{{\mathrm{Bu}}}}}}\right){\left({{{{{{\mathrm{NEt}}}}}}}_{2}\right)}_{3}^{*}+{{{{{{\mathrm{OH}}}}}}}^{*} \to {{{{{\mathrm{Ta}}}}}} {\left({{{{{{\mathrm{NEt}}}}}}}_{2}\right)}_{3}^{*} \sim {{{{{\mathrm{O}}}}}} \sim {{{{{\mathrm{H}}}}}}{{{{{\mathrm{N}}}}}}^{{{{{\mathrm{t}}}}}}{{{{{\mathrm{Bu}}}}}}$$

H-transfer-2:

7 $${{{{{\mathrm{Ta}}}}}}\left({{{{{\mathrm{N}}}}}}^{{{{{\mathrm{t}}}}}}{{{{{\mathrm{Bu}}}}}}\right){\left({{{{{{\mathrm{NEt}}}}}}}_{2}\right)}_{2}^{*} \sim {{{{{\mathrm{O}}}}}}+{{{{{{\mathrm{OH}}}}}}}^{*} \to {{{{{\mathrm{Ta}}}}}}\left({{{{{\mathrm{N}}}}}}^{t}{{{{{\mathrm{Bu}}}}}}\right)\left({{{{{{\mathrm{NEt}}}}}}}_{2}\right)*\sim {{{{{\mathrm{O}}}}}} \sim {{{{{\mathrm{O}}}}}}+{{{{{\mathrm{HNE}}}}}} {t}_{2} ({{{{{\mathrm{g}}}}}})$$

Although two reactions for H-transfer-1 are both possible, the proton is preferentially transferred to the -NEt_2_ ligands than the -N^t^Bu ligand on the SiO_2_ surface. Therefore, hydrogenation of the -NEt_2_ ligands is mainly considered in the following H-transfer reaction. H-transfer reactions are exothermic with a low reaction barrier on OH-terminated SiO_2_, indicating that reactions could occur easily on OH-terminated surfaces. Overall, the Ta(N^t^Bu)(NEt_2_)_3_ precursor shows lower reactivity on reduced Cu than that on OH-terminated SiO_2_ because of the higher energy barrier, which is the origin of the selectivity between the reduced Cu and OH-terminated SiO_2_.

### Selective ALD on Cu/SiO_2_ nanopatterns

**Fig. 4:**
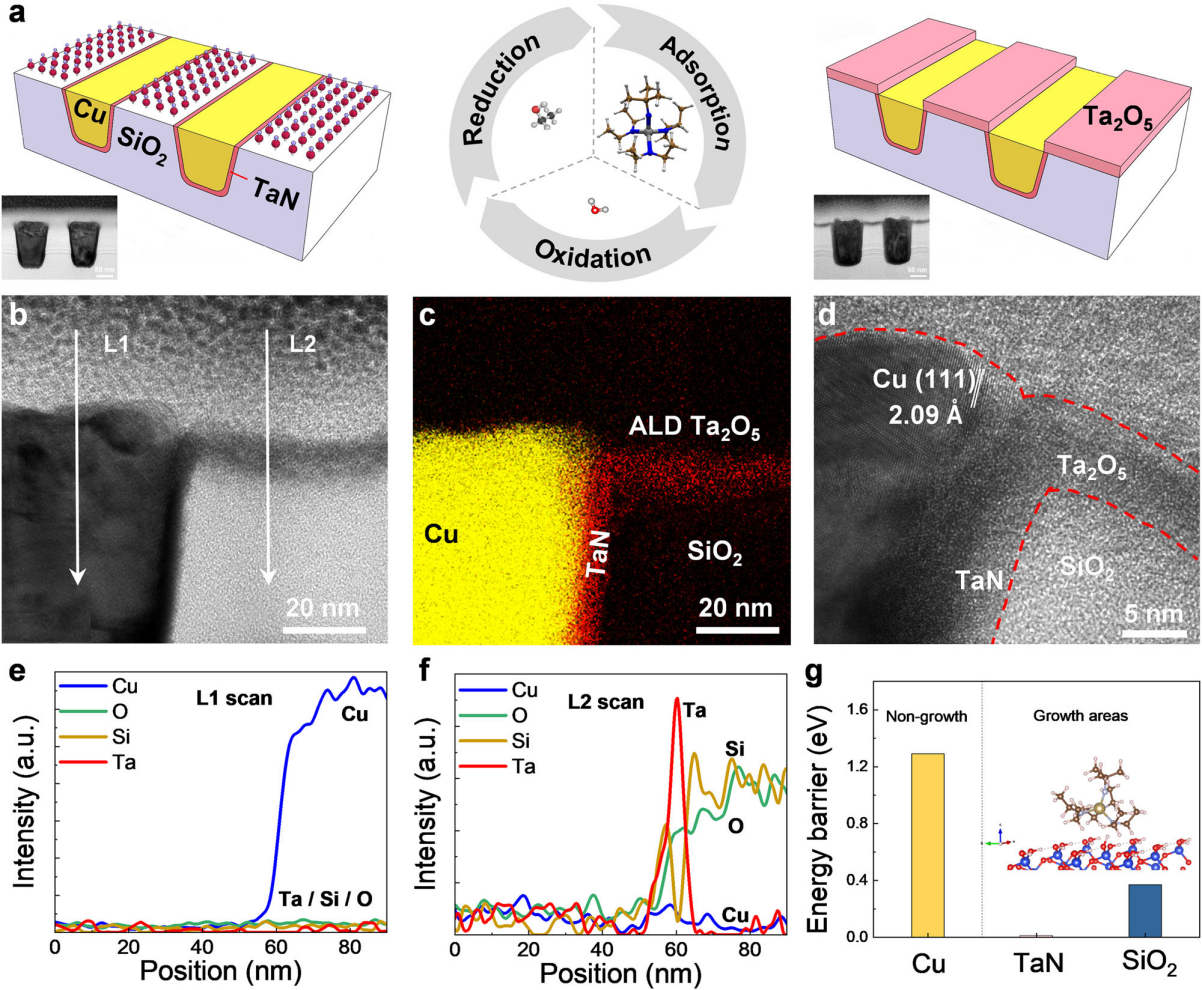
The characterizations of self-aligned TaO_x_ deposited on Cu/SiO_2_ nanopatterns. **a** The scheme and TEM image of original 50 nm critical dimension Cu/SiO_2_ patterns and self-aligned patterning of TaO_x_ film after redox-coupled inherently selective ALD. **b** The cross-section TEM image of self-aligned TaO_x_ film on the Cu/SiO_2_ pattern. The L1 and L2 mean the region 1 and 2 for line scan of Cu, O, Si, and Ta elements, respectively. **c** The element mapping of Cu and Ta. **d** The high-resolution TEM image of the border between Cu and SiO_2_. The line scan across the **e** Cu and **f** SiO_2_ region after 100 ALD cycles, respectively. **g** The DFT calculation of TaO_x_ deposited on Cu, TaN, and SiO_2_, the nucleation on the Cu region is inhibited.

To directly observe the selective deposition on the nanopattern structures, the cross-section TEM of TaO_x_ deposited on the Cu/SiO_2_ nanopatterns in a dense via-chain array is performed. Cu/SiO_2_ patterns were prepared from the chip production line. Schemes and TEM images of the original Cu/SiO_2_ patterns and self-aligned patterned Ta_2_O_5_ films prepared by redox-coupled inherently selective ALD are presented in Fig. 4a. The pitch of the Cu/SiO_2_ patterns is ~100 nm, and the critical dimension is ~50 nm. After ABC-type ALD, the SiO_2_ surface is coated with conformal Ta_2_O_5_ film, whereas the Cu region remains in its original morphology without any Ta_2_O_5_ deposition (Fig. 4b**)**. Through EDS mapping, Ta is detected on the Si region while no signals on Cu regions (Fig. 4c**)**. The element mappings of Si, Cu, and Ta are presented in Supplementary Fig. 24. Enlarged high-resolution TEM image of the border between the Cu and SiO_2_ is presented in Fig. 4d. The Cu feature is non-oxidized, and the crystallographic orientation is (111). In the enlarged images, there is no mushroom growth at the edge, and no defects are observed within the Cu regions. Element line scans across the Cu and SiO_2_ regions are presented in Fig. 4e, f. There is no Ta element detected on copper region, while a strong Ta signal is detected on the SiO_2_, which also confirm the 100% selectivity is achieved. DFT calculations show that the chemisorption of the Ta(N^t^Bu)(NEt_2_)_3_ precursor on the TaN barrier and SiO_2_ is thermodynamically favored (Fig. 4g). The reaction barrier on Cu is much larger. Thus, the Ta_2_O_5_ film is preferentially deposited on SiO_2_ and TaN instead of Cu, forming self-aligned patterning. High resolution TEM images of different regions also verified that TaO_x_ deposition is restricted to the TaN barrier and SiO_2_, and diffusion of Ta atoms to neighboring Cu regions is unlikely to occur, avoiding excessive mushroom growth at the edges or the emergence of undesired nucleation defects within the Cu region (Supplementary Fig. 25). Several oxides, including NbO_x_, MoO_x_, and WO_x_, have also been tried using AB and ABC-type ALD processes. The results indicated that the redox-coupled ALD method is capable to achieve higher selectivity compared to the binary ALD process, demonstrating the generality of this method (Supplementary Figs. 26–28).

In conclusion, TaO_x_ is selectively deposited on SiO_2_ before Cu for self-alignment process. The Ta(N^t^Bu)(NEt_2_)_3_ precursor has a higher energy barrier on reduced Cu surface than that of OH-terminated SiO_2_ surface, which is the origin of the selectivity. The selectivity decreases with the oxidation of Cu during ALD. Optimized selectivity is achieved with redox-coupled ABC-type (EtOH-Ta(N^t^Bu)(NEt_2_)_3_-H_2_O) ALD process, and EtOH is used to reduce the surface oxidation of Cu in situ. Moreover, the inherently selective ALD process is successfully transferred onto Cu/SiO_2_ nanopatterns with ~100 nm pitch and obtained high selectivity with 5–6 nm films on SiO_2_ and no defects in the Cu region. The results indicate that inherently selective ALD is a robust and general tool that has excellent application prospects in back-end-of-line processes, which provides an innovative avenue for self-aligned nanostructures.

## Methods

### Substrate preparation

The Si wafer with ~2 nm native oxides were used as the initial SiO_2_ substrate. The Cu films were evaporated on Si wafer with thickness of ~5 nm. The substrates were stored in an argon atmosphere until ALD process were performed. The quartz crystal oscillators were coated with ~5 nm Cu or SiO_2_ for quartz crystal microbalance (QCM) tests (Inficon, SQM-160).

### ALD process

The ALD reactions were performed in a custom-built hot-walled ALD reactor (Material Design and Nano-manufacturing center @ HUST, Wuhan, China). Ta_2_O_5_ was deposited using Ta(N^t^BU)(NEt_2_)_3_ precursor (>99.9%, Aimou Yuan, Nanjing, China) and various co-reactants, including O_3_, O_2_, H_2_O, EtOH, CH_3_COOH. All the chemicals are high purity >99.9%. Ozone was prepared with high-purity oxygen (99.999%) through an ozone generator (11 vol% of O_3_ in O_2_). The heating temperature of the Ta precursor steel bubbler was 65 °C. The high-purity Ar gas (40sccm, 99.999%) was injected into the bubbler and carried precursors dosed into the ALD cavity. The pipeline temperature was up to 90 °C to prevent the precursors’ condensation.

### Characterization methods

The film thickness was measured by a spectroscopic ellipsometer (M-200X, J. A. Woollam Co.). The modified TaO_x_ Cauchy model was used to fit the ellipsometer data through the Complete EASE software. J.A.Woollam M2000 spectroscopic ellipsometry was utilized to collect data. Each data point of the film thickness is measured at two positions of one sample by spectroscopic ellipsometry. Error bars represent standard deviations after two measurements of each sample. The surface composition was detected by X-ray photoelectron spectroscopy (XPS, AXIS-ULTRA DLD-600 W). The surface morphology was determined by atomic force microscopy (AFM, SPM9700). AFM was performed in tapping mode using a Molecular Imaging PicoScan Controller. Aluminum reflex coated Si AFM probes were used. Data were processed and analyzed using Gwyddion 2.49 software. The cross-section films were analyzed by transmission electron microscopy (TEM, Talos F200X) with bright-field and annular dark-field scanning modes, and the element distribution was analyzed by energy dispersive X-ray spectroscopy (EDS). The electrical measurements were performed through capacitance-voltage using a Keithley 4200 impedance analyzer. 100 nm thick Ag film was evaporated as the back electrode, circular Ag electrode with 200 um diameter and 100 nm thickness was evaporated on target film through a shadow mask served as the front side electrode. Capacitance measurements were conducted at 500 kHz 100 mV ac modulation while the DC gate voltage was swept from −4 V to 4 V. DFT calculations were carried out through first-principles plane-wave pseudopotential formulation implemented in the Vienna ab-initio Simulation Package^59^. The exchange−correlation function was in the form of Perdew–Burke–Ernzerhof with the generalized gradient approximation^60^. Van der Waals interactions were also considered using the DFT-D3 method^61^. A 6.5-Å-thick SiO_2_ (001) surface with hydroxylation was built to resemble the experimental surface. The nudged elastic band method was used to locate the transition state between two local minima states. Gibbs correction was proposed for precursor adsorption using VASPKIT^62^ at the temperature of 473 K.

### Reporting summary

Further information on research design is available in the Nature Portfolio Reporting Summary linked to this article.

## Supplementary information

Supplementary Information

Peer Review File

Reporting Summary

DFT configurations

## Data Availability

All data in this study are available in the manuscript and in the Supplementary information section. Source data are provided with this paper.

## Source data

Source Data
