# Supplementary material for: Self-aligned patterning of tantalum oxide on Cu/SiO2 through redox-coupled inherently selective atomic layer deposition
Source: Nat Commun. 2023 Jul 26;14:4493. doi: 10.1038/s41467-023-40249-2 (PMC10372027; doi:10.1038/s41467-023-40249-2)
Supplement: Supplementary file 1 — Supplementary Information [file 41467_2023_40249_MOESM1_ESM.pdf]

Supplementary Information

Self-Aligned Patterning of Tantalum Oxide on Cu/SiO_2 through Redox-coupled Inherently Selective Atomic Layer Deposition

Yicheng Li,[1,4] Zilian Qi,[1,4] Yuxiao Lan,[2] Kun Cao,[1]* Yanwei Wen,[2] Jingming Zhang,[2] Eryan Gu,[1] Junzhou Long,[1,3] Jin Yan,[1] Bin Shan,[2] and Rong Chen,[1,3]*

[1]State Key Laboratory of Intelligent Manufacturing Equipment and Technology, School of Mechanical Science and Engineering, Huazhong University of Science and Technology, Wuhan 430074 Hubei, People's Republic of China
[2]State Key Laboratory of Materials Processing and Die & Mould Technology, School of Materials Science and Engineering, Huazhong University of Science and Technology, Wuhan 430074 Hubei, People's Republic of China
[3]Hubei Yangtze Memory Laboratories, Wuhan 430205 Hubei, People's Republic of China
[4]These authors contributed equally: Yicheng Li, Zilian Qi
*Corresponding Author. E-mail: kuncao@hust.edu.cn, rongchen@mail.hust.edu.cn

This file includes:

Supplementary Figures 1 to 28
Supplementary Tables 1 to 3
References (1 to 12)

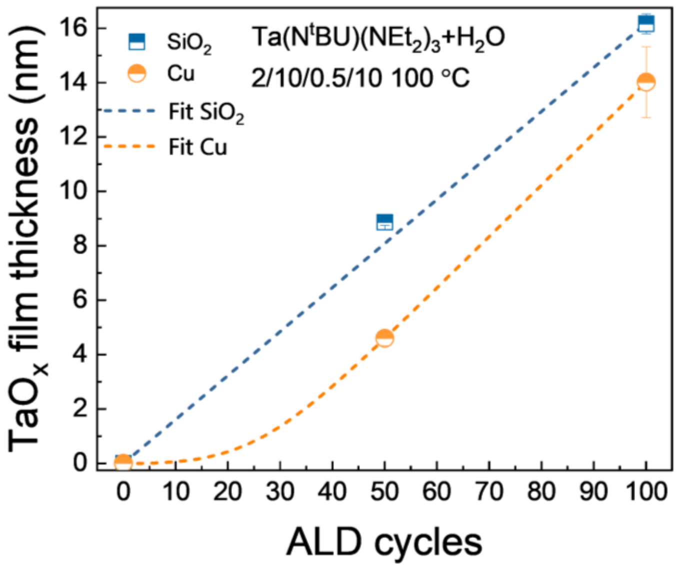

Supplementary Fig. 1 The films thickness of TaO_x prepared by AB-type ALD process with Ta(NtBu)(NEt_2)$_3$-H_2O as precursors at 100 ˚C. The average growth rate on SiO_2 and Cu is 0.16 and 0.14 nm/cycle within 100 cycles, respectively. Each data point of the film thickness is measured at two positions of one sample by spectroscopic ellipsometry. Error bars represent standard deviations after two measurements of each sample.

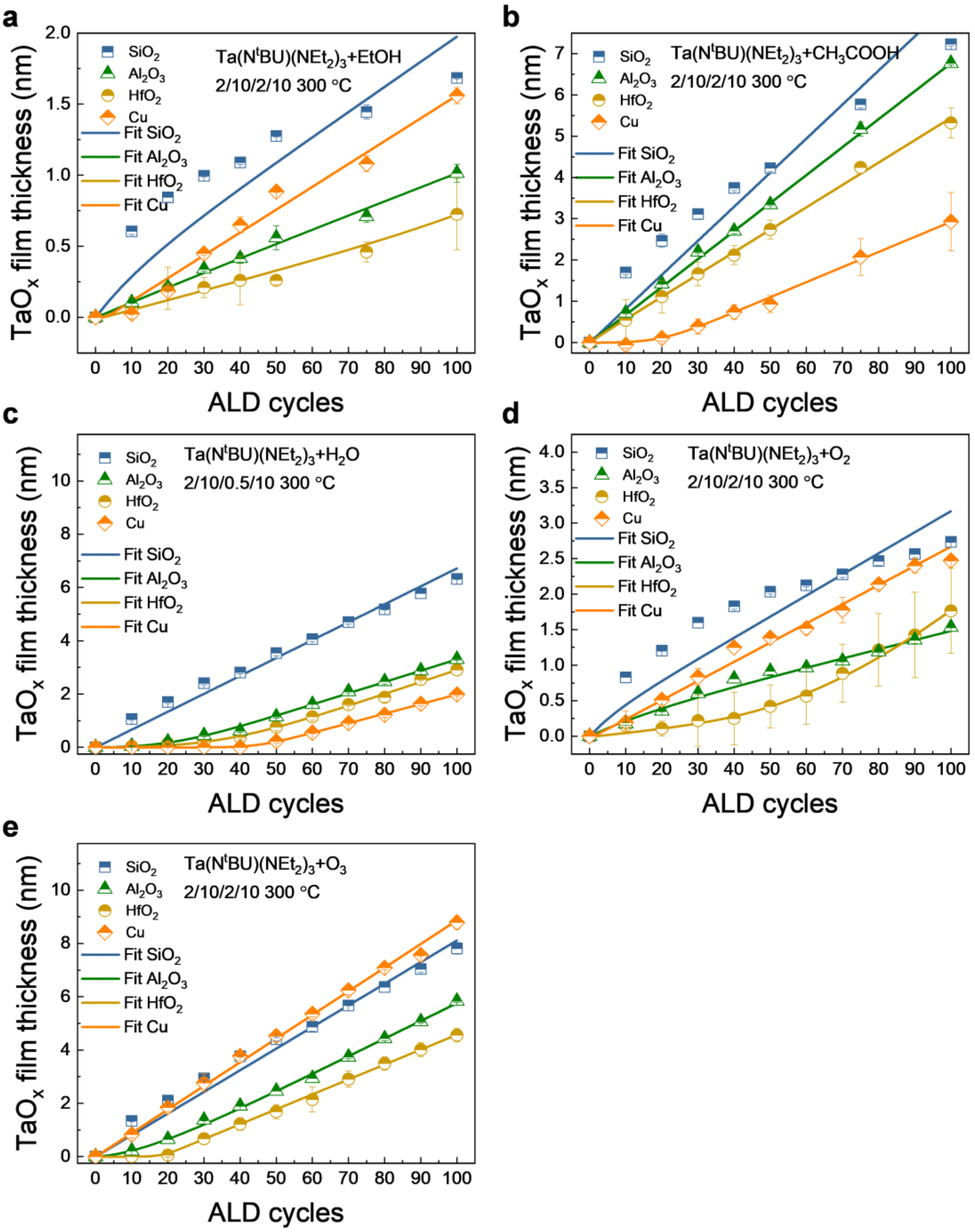

Supplementary Fig. 2 The films thickness of TaO_x prepared by AB-type ALD process with $Ta(NtBu)(NEt_2)_3$ and another co-reactant as precursors at 300 ˚C. The ALD process was performed on four substrates include SiO_2, Al_2O_3, HfO_2, and Cu. by using (**a**) EtOH, (**b**) CH_3COOH, (**c**) H_2O, (**d**) O_2, and (**e**) O_3 as co-reactants. Each data point of the film thickness is measured at two positions of one sample by spectroscopic ellipsometry. Error bars represent standard deviations after two measurements of each sample.

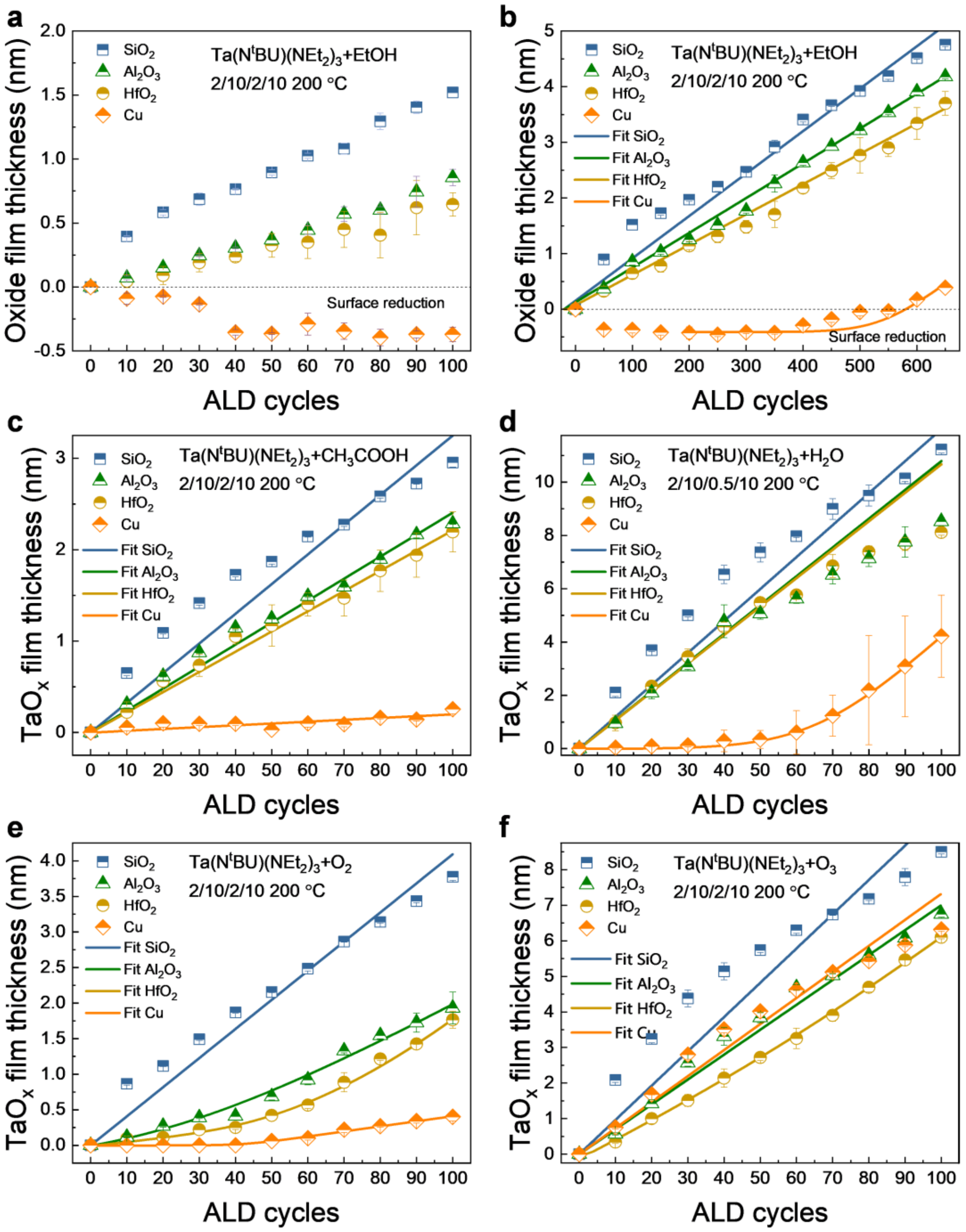

Supplementary Fig. 3 The films thickness of TaO_x prepared by AB-type ALD process with $Ta(NtBu)(NEt_2)_3$ and another co-reactant as precursors at 200 ˚C. The ALD process was performed on four types of substrates include SiO_2, Al_2O_3, HfO_2, and Cu. with (**a-b**) EtOH, (**c**) CH_3COOH, (**d**) H_2O, (**e**) O_2, and (**f**) O_3 as co-reactants. Each data point of the film thickness is measured at two positions of one sample by spectroscopic ellipsometry. Error bars represent standard deviations after two measurements of each sample.

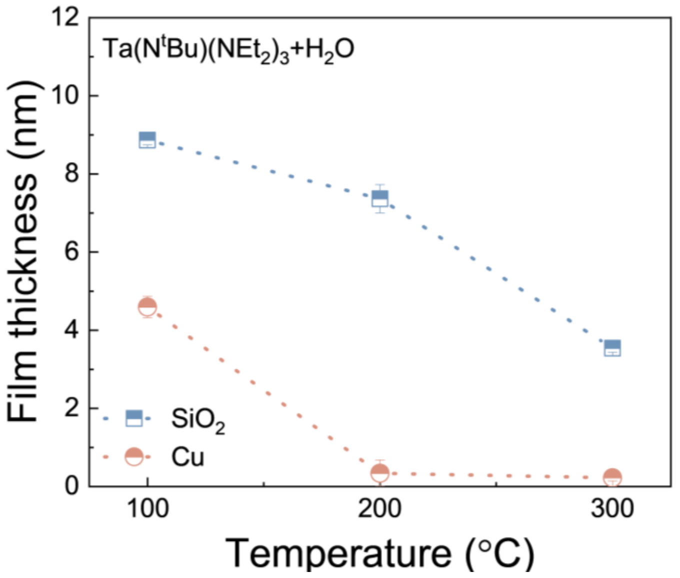

Supplementary Fig. 4 The film thickness as a function of deposition temperature on SiO_2 and Cu. 50 cycles AB-type ALD process was performed with Ta(NtBu)(NEt$_2$)$_3$-H_2O as precursors at 100 °C, 200 °C, and 300 °C, respectively. Each data point of the film thickness is measured at two positions of one sample by spectroscopic ellipsometry. Error bars represent standard deviations after two measurements of each sample.

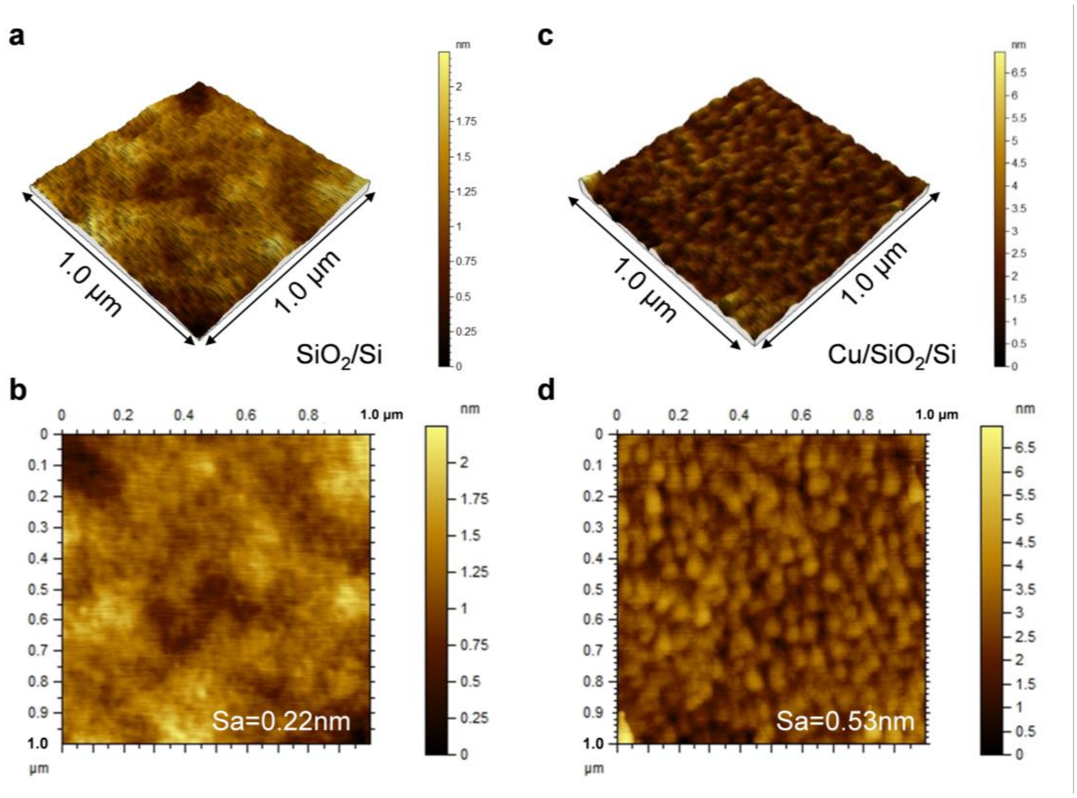

Supplementary Fig. 5 The AFM images of (a-b) SiO_2 and (c-d) Cu substrates. The surface roughness of SiO_2/Si wafer and Cu thin film prepared on Si wafer was measured by AFM. The RMS roughness were 0.22 nm and 0.53 nm for SiO_2 and Cu, respectively. Barely particles were observed on the SiO_2 and Cu surface after 50, 100, and 150 ABC-type ALD cycles.

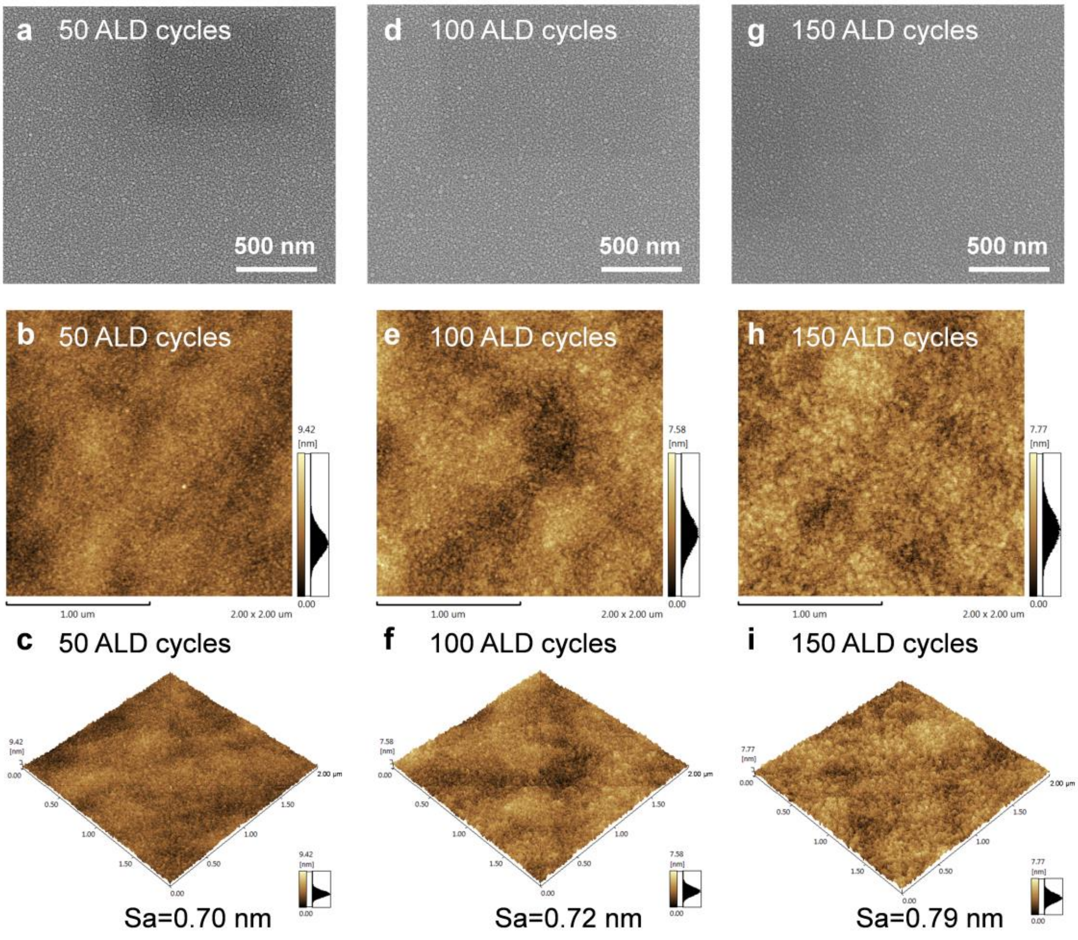

Supplementary Fig. 6 SEM and AFM images of Cu substrates after 50, 100, and 150 ALD cycles. (**a**) SEM, (**b**) AFM and (**c**) 3-D AFM images after 50 ALD cycles; (**d**) SEM, (**e**) AFM and (**f**) 3-D AFM images after 100 ALD cycles; (**g**) SEM, (**h**) AFM and (**i**) 3-D AFM images after 150 ALD cycles

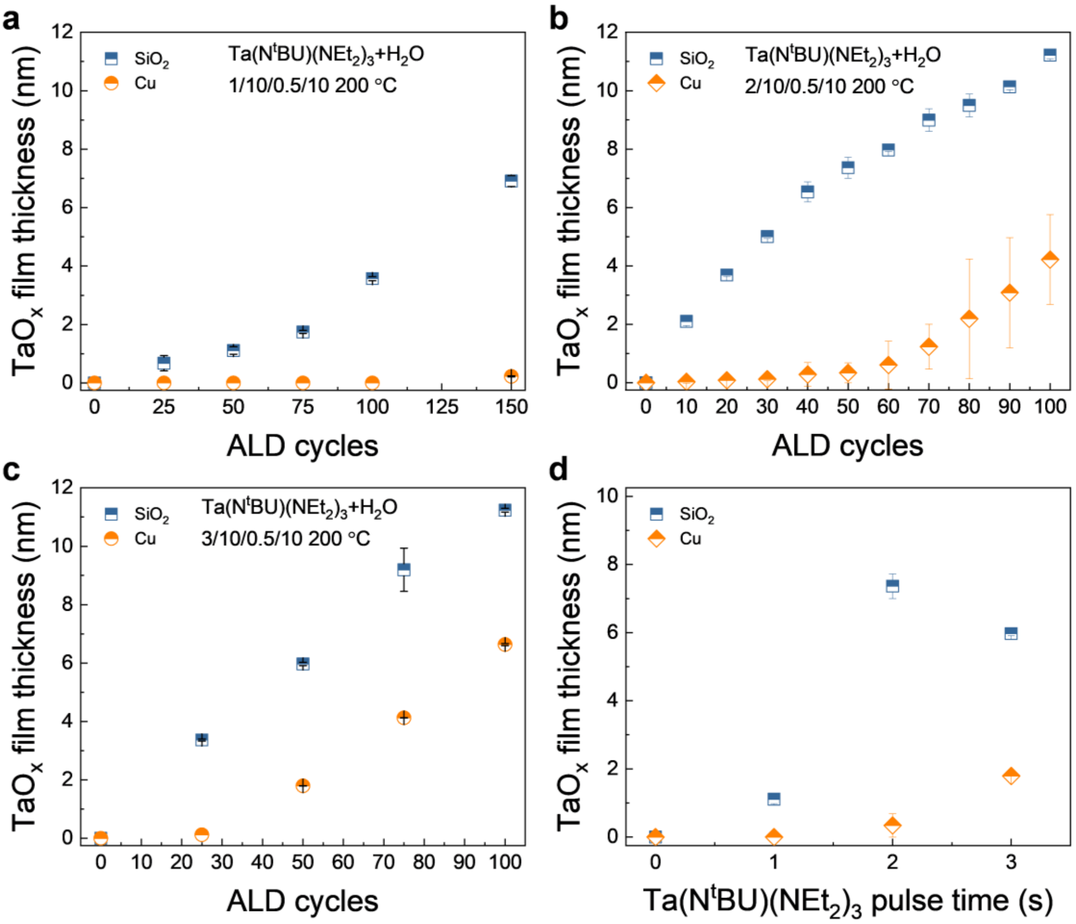

Supplementary Fig. 7 The effect of pulse time during AB-type ALD process. The TaO_x film thickness as a function of the number of ALD cycles, and the precursors' pulse and purge processes are (**a**) 1-10-0.5-10s, (**b**) 2-10-0.5-10s, and (**c**) 3-10-0.5-10s, respectively, in seconds; (**d**) The relationship between TaO_x film thickness and Ta(N^{t}BU)(NEt$_2$)$_3$ pulse time at the deposition temperature of 200°C. Each data point of the film thickness is measured at two positions of one sample by spectroscopic ellipsometry. Error bars represent standard deviations after two measurements of each sample.

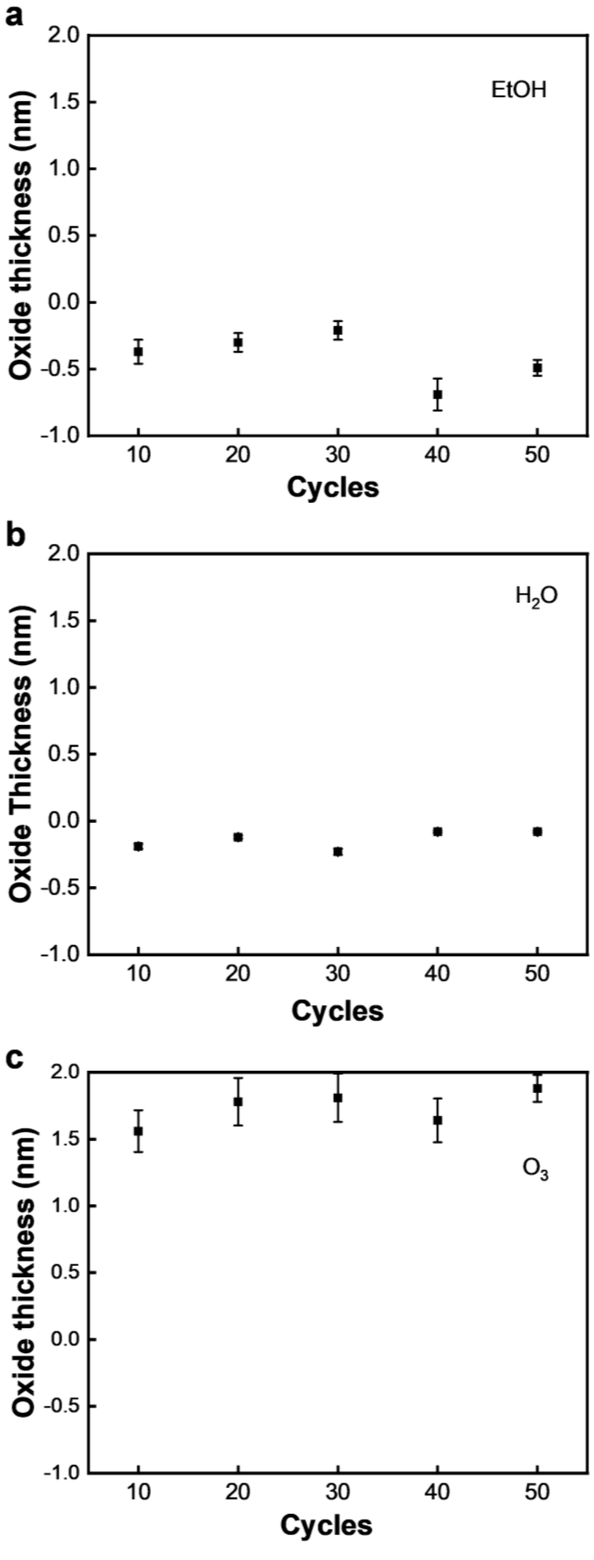

Supplementary Fig. 8 Bare Cu substrates tested using SE after exposure to ethanol, H_2O, O_3 pulses at 200 °C. The decrease of Cu's surface native oxide layer thickness with ethanol pretreatment is ~0.5nm. H_2O has minimal influence on the surface oxide layer, while O_3 strongly oxidized the Cu surface, thereby increasing the surface oxide layer thickness. Each data point of the film thickness is measured at two positions of one sample by spectroscopic ellipsometry. Error bars represent standard deviations after two measurements of each

sample.

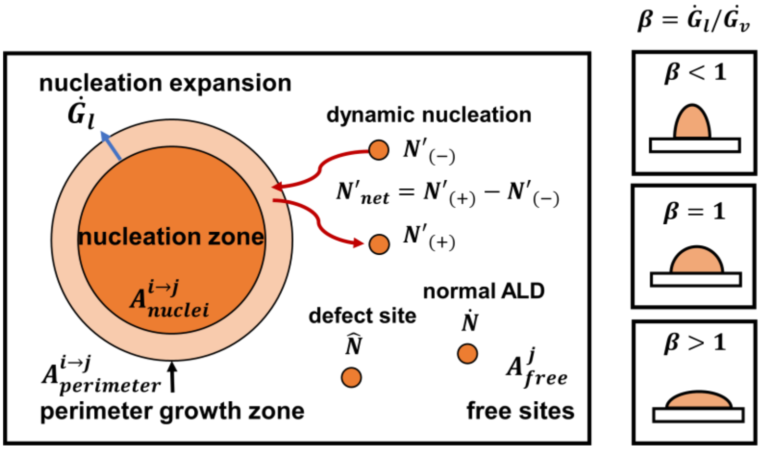

Supplementary Fig. 9 The nucleation model schematic diagram.

Nucleation Modeling:

The nucleation model includes five independent parameters: (1) the ALD growth rate of the material deposition on itself along the vertical direction per cycle $\dot{G}_v$ (nm/cycle), (2) the ALD growth rate of the nuclei expansion on the substrate along the lateral direction per cycle $\dot{G}_l$ (nm/cycle), (3) the nucleation site density induced per ALD cycle on non-defect sites $\dot{N}$ (nm^{-2}), (4) the defect-induced nucleation site density during the initial ALD cycle $\hat{N}$ (nm^{-2}), and (5) the atomic diffusion induced nucleation in the dynamic expanding region at the edge of nucleus $\dot{N}'$ (nm^{-2}). The perimeter of the growth zone represents the circumferential growth area around the nuclei. Growth along the lateral direction $\dot{G}_l$ is characterized by the nuclei-substrate-precursor triple boundary and its rate is different from the intrinsic deposition rate. The parameter β is used to denote the ratio of $\dot{G}_l$ to $\dot{G}_v$.The remaining zones on the surface were free sites. The areas corresponding to these three zones after the jth ALD cycle are denoted by A^{j}_{nuclei}, $A^{j}_{perimeter}$, and A^{j}_{free}, respectively. During the jth cycle, the nuclei density can be described as follows:

$$N^{j} = \frac{A^{j-1}_{free}}{A}\left(\dot{N} + \frac{A^{j-1}_{nuclei}}{A}\dot{N}'\right)\ (j \geq 2) \tag{1}$$

After the jth cycle, the nucleation area includes two parts:

$$A^{i\to j}_{nuclei} = A^{i\to(j-1)}_{nuclei} + A^{i\to(j-1)}_{perimeter}\ (1 \leq i \leq j-1) \tag{2}$$

Area of perimeter zone corresponds to nucleus expansion along the lateral direction, the evolution of which can be approximated by multiplying the lateral growth rate ($\dot{G}_l = \beta\dot{G}_v$) with the perimeter of the nuclei $L^{i\to(j-1)}$, and normalized by the fraction of free sites:

$$A_{perimeter}^{i\to j-1} = \beta\dot{G}_v \left(\frac{A_{free}^{j-2}}{A}\right) L^{i\to(j-1)} \qquad (1 \le i \le j-1) \tag{3}$$

And the nuclei (seed generated during the ith cycle) perimeter $L^{i\to j-1}$ after the (j-1)^th^ cycle can be estimated from geometrical considerations. Here we estimate the nucleus is hemisphere, so the circular projection of the nucleus on substrate is circle, the relationship between the nucleus area and perimeter can be deduced:

$$A_{nuclei}^{i\to(j-1)} / n^i = \pi\left(\frac{L^{i\to(j-1)}}{2\pi n^i}\right)^2 \tag{4}$$

n^i is the number of nuclei in i^{th} cycle ($n^i = A \cdot N^{\mathrm{i}}$). We re-write Eq. (4) in the following form:

$$L^{i\to(j-1)} = 2\sqrt{\pi}\sqrt{A_{nuclei}^{i\to(j-1)} n^i} \tag{5}$$

The perimeter area for nuclei after the jth cycle can be obtained as:

$$\mathrm{A_{perimeter}^{i\to j}} = \beta\dot{\mathrm{G}}_\mathrm{v}\alpha\left(\frac{\mathrm{A_{free}^{j-1}}}{\mathrm{A}}\right)\sqrt{\mathrm{A_{nuclei}^{i\to j} n^i}},\ \alpha = 2\sqrt{\pi} \tag{6}$$

The area of the free site zone after the jth cycle decreases owing to the accumulation of j cycles of ALD growth:

$$\mathrm{A_{free}^{j}} = \mathrm{A} - \sum_{\mathrm{i=1}}^{\mathrm{j}}\left(\mathrm{A_{nuclei}^{i\to j}} + \mathrm{A_{perimeter}^{i\to j}}\right) \tag{7}$$

Through the recurrence relations, it is straightforward to obtain the value of $\mathrm{A_{nuclei}^{i\to j}}$, $\mathrm{A_{perimeter}^{i\to j}}$, and $\mathrm{A_{free}^{j}}$ iteratively with the initial value of $\widehat{\mathrm{N}}$, $\dot{\mathrm{N}}$, $\dot{\mathrm{N}}'$, $\dot{\mathrm{G}}_\mathrm{v}$, and β. Such model is tested to well fit the experimental data [ref. 1] of the selectively growth on carbon substrates with MAE=0.081.

Supplementary Table 1. Summary of the thicknesses for TaO_x on different substrates and GPCs and fitting values of G_v

process	SiO_2	Al_2O_3	HfO_2	Ratio Al_2O_3/SiO_2	Fitting values
Ta-H_2O 200°C 100 cycles	11.22	8.525	8.115	76.0%	2.3/3.7=62.3%
Ta-EtOH 200°C 100 cycles	1.52	0.855	0.645	56.3%	0.64/0.78=82.1%
Ta-HAc 200°C 100 cycles	2.955	2.285	2.195	77.3%	2.4/3.2=75.0%
Ta-O_2 200°C 100 cycles	3.775	1.93	1.765	51.1%	0.97/4.1=23.7%
Ta-O_3 200°C 100 cycles	8.505	6.755	6.1	79.4%	7.0/9.6=72.9%
EtOH-Ta-H_2O 200°C 100 cycles	3.64	3.005	2.575	82.6%	2.3/3.7=62.2%
HAc-Ta-H_2O 200°C 100 cycles	8.52	7.33	6.75	86.0%	8.1/9.1=89%
H_2O-Ta-O_3 200°C 100 cycles	11.8	9.755	8.235	82.7%	8.1/13=62.3%

Supplementary Table 2. The fit parameters for Cu substrates, β was set as 1.

Chemical states	$\hat{N}$ (nm^{-2})	$\dot{N}$ (nm^{-2})	$\dot{N}'$ (nm^{-2})	$\dot{G}_v$ (nm/cycle)	β	f_{err}
EtOH-treated Cu	$3.3*10^{-4}$	$1.0*10^{-5}$	$4.3*10^{-3}$	$1.2*10^{-1}$	1.0	$2.0*10^{-2}$
HAc-treated Cu	$3.0*10^{-2}$	$4.0*10^{-4}$	$1.5*10^{-3}$	$6.2*10^{-2}$	1.0	$3.8*10^{-2}$
O_2-treated Cu	$6.6*10^{-3}$	$9.8*10^{-4}$	$1.5*10^{-2}$	$1.4*10^{-1}$	1.0	$1.4*10^{-2}$
O_3-treated Cu	$5.2*10^{-2}$	$4.0*10^{-2}$	$1.3*10^{-1}$	$1.5*10^{-1}$	1.0	$1.7*10^{-2}$

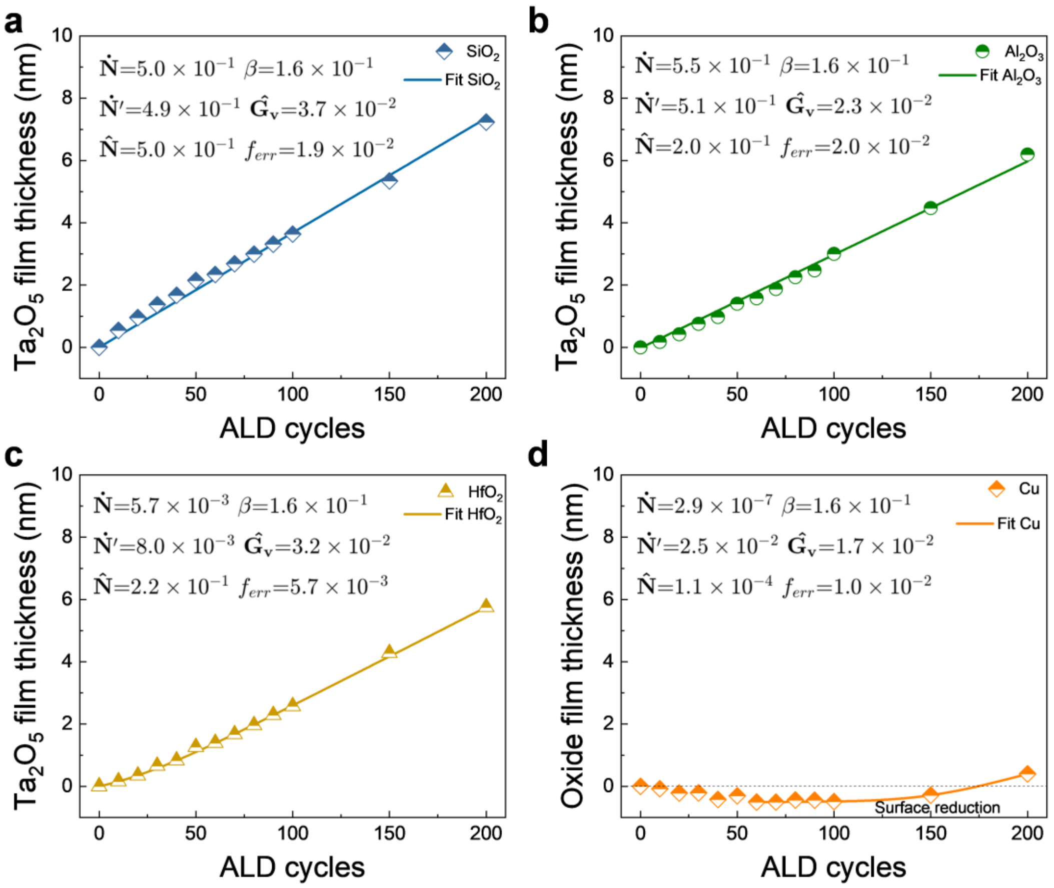

Supplementary Fig. 10 The experimental and fitting film thickness as a function of the ALD cycles. The ABC-type ALD processes with EtOH-Ta(N^tBu)(NEt_2)$_3$-H_2O as reactants at 200 °C are performed on (**a**) SiO_2, (**b**) Al_2O_3, (**c**) HfO_2, and (**d**) Cu substrates. The values of $\dot{N}$ and $\dot{N}'$ on the HfO_2 substrate are on the order of 10^{-3} nm^{-2}, which is significantly lower than the values on the order of 10^{-1} nm^{-2} on the SiO_2 and Al_2O_3 substrates. The values of $\dot{N}$ and $\hat{N}$ on Cu are 2.9×10^{-7} nm^{-2} and 1.1×10^{-4} nm^{-2}, respectively, which are much lower than those on oxide substrates. The results suggest that defect-induced nucleation is limited on Cu. The experimental data are obtained from source data of figure 1b in the main text. Each data point of the film thickness is measured at two positions of one sample by spectroscopic ellipsometry. Error bars represent standard deviations after two measurements of each sample.

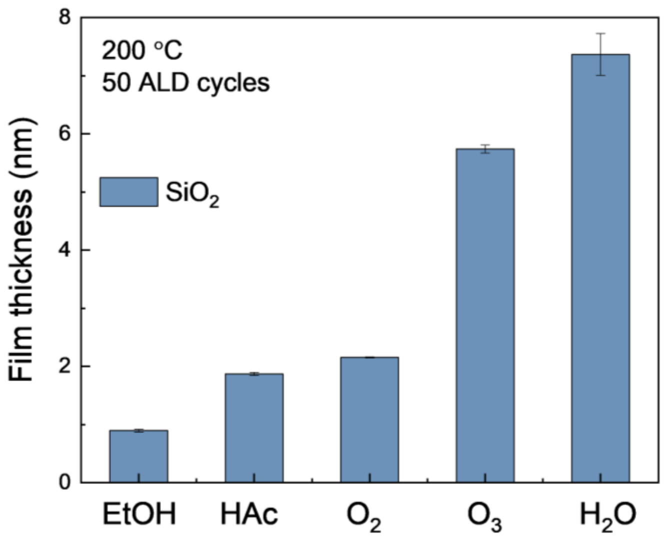

Supplementary Fig. 11 The TaO_x film thickness on SiO_2 as a function of different co-reactants. The AB-type ALD is performed, the growth rate on SiO_2 with different co-reactants decrease as follows: $H_2O > O_3 > O_2$ > HAc > EtOH. For AB-type ALD process, the pulse time for $Ta(N^tBu)(NEt_2)_3$ is 2s, the pulse time for O_3, O_2, Hac, and EtOH is 2s, for H_2O is 0.5s. The purge time is 10s. Each data point of the film thickness is measured at two positions of one sample by spectroscopic ellipsometry. Error bars represent standard deviations after two measurements of each sample.

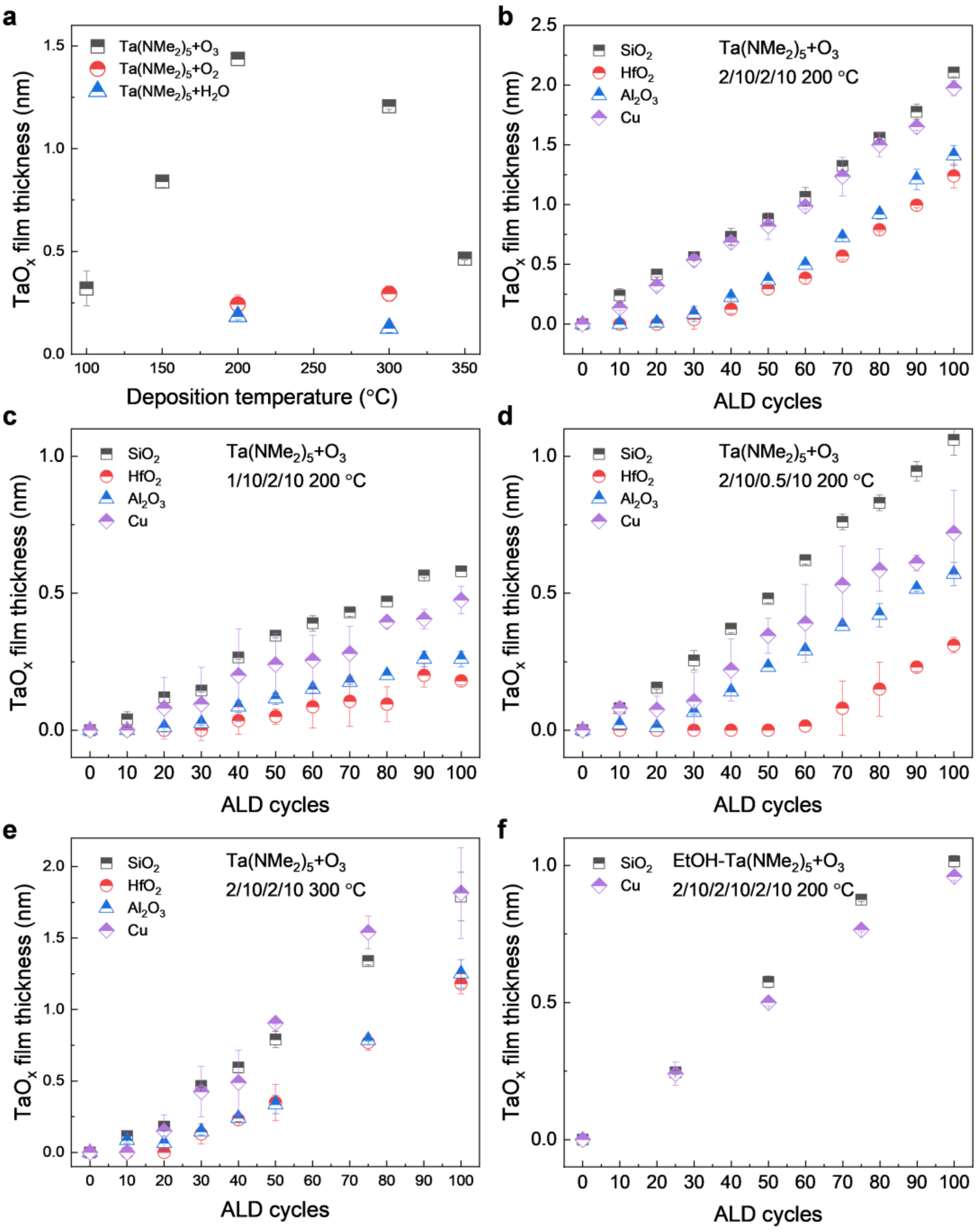

Supplementary Fig. 12 The film thickness as a function of the ALD cycles on SiO_2 and Cu substrates at 200 ˚C by exploiting $Ta(NMe_2)_5$ precursor. (**a**) The ALD of $Ta(NMe_2)_5$ with different co-reactants, including H_2O, O_2, and O_3. H_2O and O_2 are not active enough to initiate growth of TaO_x at 200 ˚C. The ALD growth rate of $Ta(NMe_2)_5$ and O_3 with ALD processes (**b**) 2-10-2-10s, (**c**) 1-10-2-10s, and (**d**) 2-10-0.5-10s, respectively. By regulating the pulse time of the precursor (2 seconds shortened to 1 second), there are only 10 cycles of nucleation delay on Cu, and the selectivity window is narrow. (**e**) The corresponding relationship between TaO_x film thickness and ALD cycle number at deposition temperature of 300 ˚C with 2-10-2-10s. (**f**) The TaO_x film thickness as a function of ALD cycles by ABC-type ALD of EtOH (2s)- $Ta(NMe_2)_5$ (2s)-O_3 (2s). The growth rate on Cu and on SiO_2 is similar with no nucleation delay. Each data point of the film thickness is measured at two positions of one sample by spectroscopic ellipsometry. Error bars represent standard deviations after two measurements of each sample.

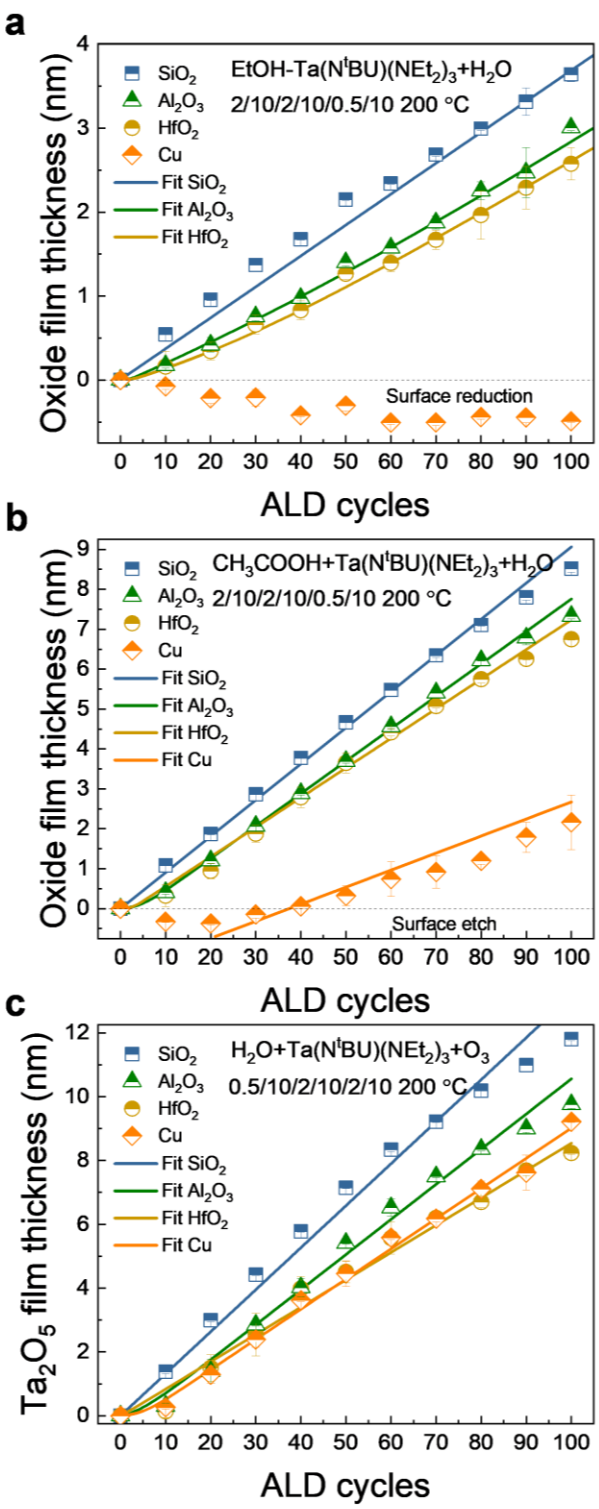

Supplementary Fig. 13 The TaO_x film thickness as a function of the ALD cycles on four substrates at 200 ˚C with three ABC-type ALD processes. (**a**) EtOH-Ta(N^tBu)(NEt_2)$_3$-H_2O, (**b**) CH_3COOH-Ta(N^tBu)(NEt_2)$_3$-H_2O and (**c**) H_2O-Ta(N^tBu)(NEt_2)$_3$-O_3. The ellipsometry of a sample is measured twice at different positions. Each data point of the film thickness is measured at two positions of one sample by spectroscopic ellipsometry. Error bars represent standard deviations after two measurements of each sample.

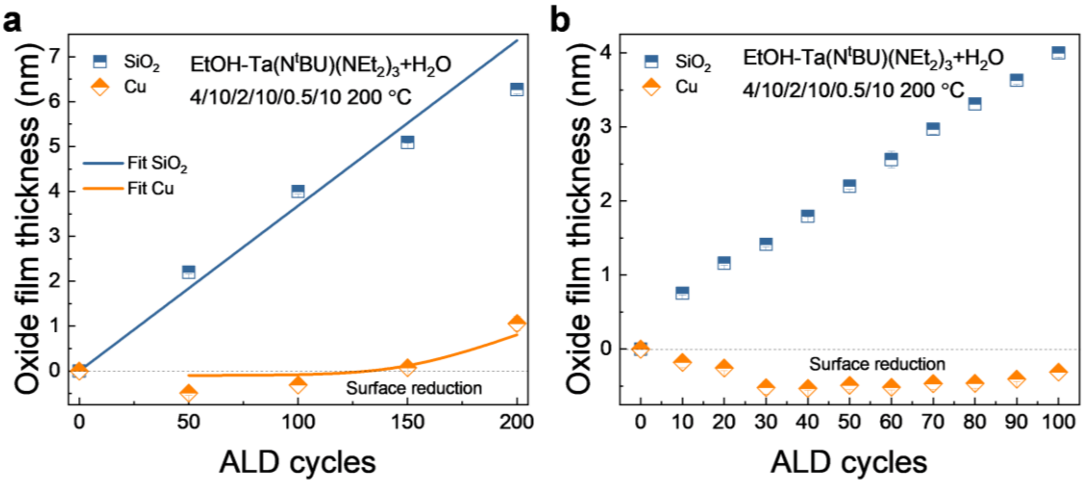

Supplementary Fig. 14. The film thickness as a function of the ALD cycles. (**a**) 200 ALD cycles and (**b**) initial 100 cycles on Cu and SiO_2 at 200 °C by using an ABC-type (EtOH-Ta(N^tBu)(NEt_2)$_3$-H_2O) with the EtOH pulse time of 4 s. Each data point of the film thickness is measured at two positions of one sample by spectroscopic ellipsometry. Error bars represent standard deviations after two measurements of each sample.

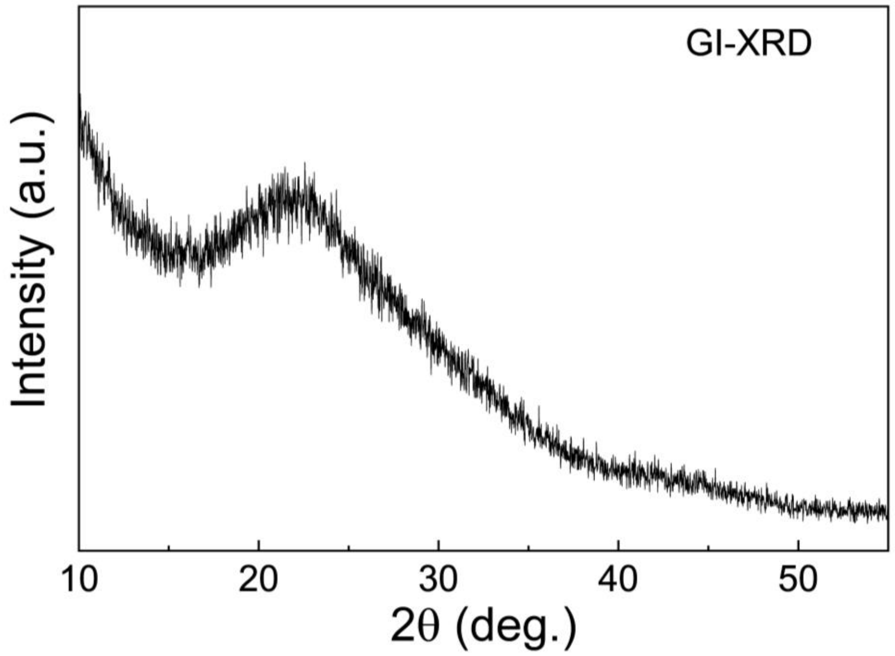

Supplementary Fig. 15 The GI-XRD of the TaO_x thin film. TaO_x film is prepared on SiO_2 after 150 ABC-type ALD cycles.

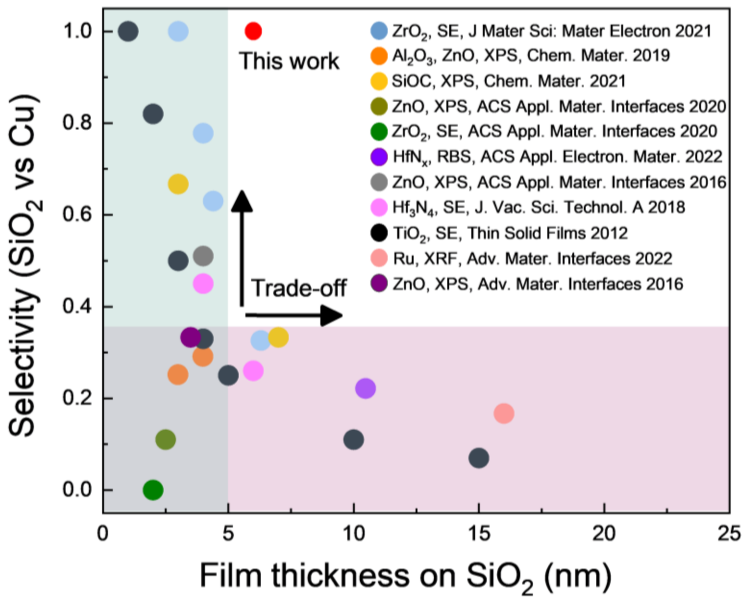

Supplementary Fig. 16. The comparison of selectivity and film thickness on growth area of SiO_2 with inherently selective ALD approaches. (ref. 2-12)

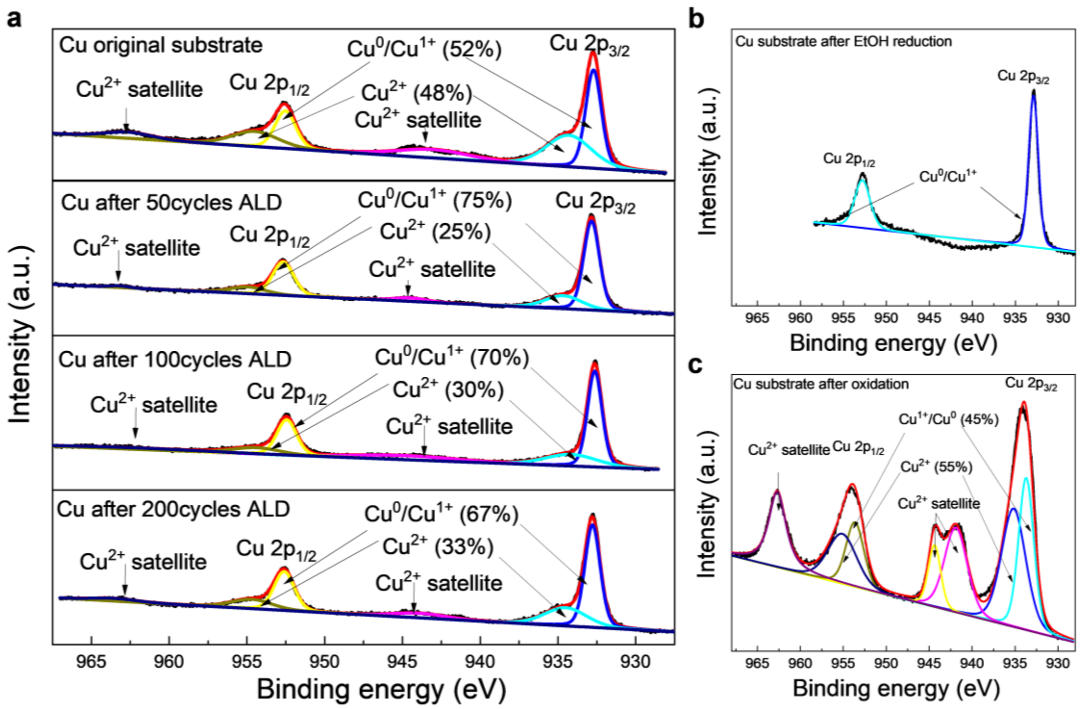

Supplementary Fig. 17 The high-resolution XPS scan of Cu 2p. (**a**) XPS tests of Cu surfaces after 0, 50, 100, and 200 ALD cycles; (**b**) after EtOH immersion and (**c**) O_2 treatment. The original Cu surface has Cu^{2+} proportion of 48%, EtOH is able to reduce the Cu oxidation, Cu^{2+} satellite peak is not observed. During ABC-type ALD process, the Cu^{2+} concentration decreases to 25% after 50 cycles, indicating reduction of the Cu surface during EtOH pulses. As the number of ALD cycles increase, the Cu oxidation state slightly increase to 30% for 100 ALD cycles and 33% for 200 ALD cycles.

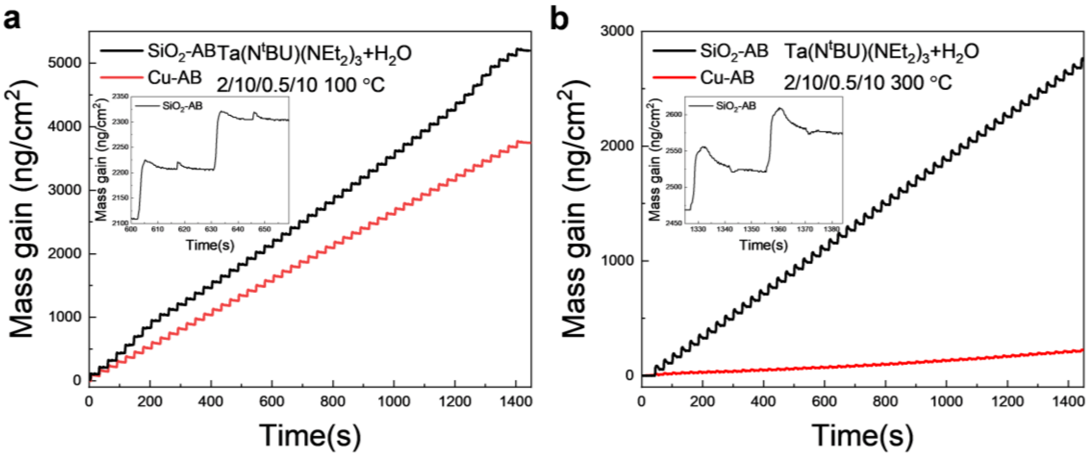

Supplementary Fig. 18 The mass gain of TaO_x deposited on Cu and SiO_2. (a) AB-type ($Ta(N^tBu)(NEt_2)_3$-H_2O) ALD at (**a**) 100 ˚C and (**b**) 300 ˚C. The insets show the amplified mass gain at 100 and 300 ℃.

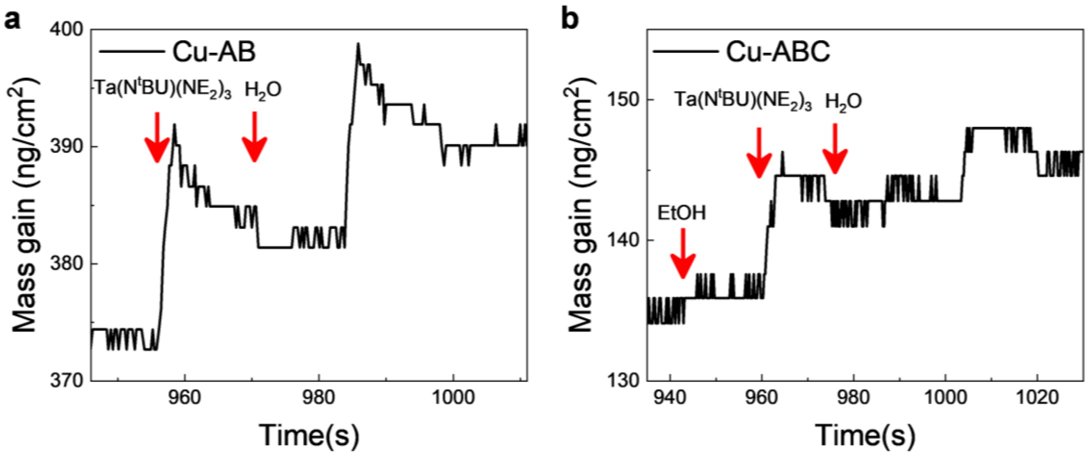

Supplementary Fig. 19 The mass gain of TaO_x deposited on Cu. (**a**)AB-type ($Ta(N^tBu)(NEt_2)_3$-H_2O) and (**b**) ABC-type (EtOH-$Ta(N^tBu)(NEt_2)_3$-H_2O) ALD.

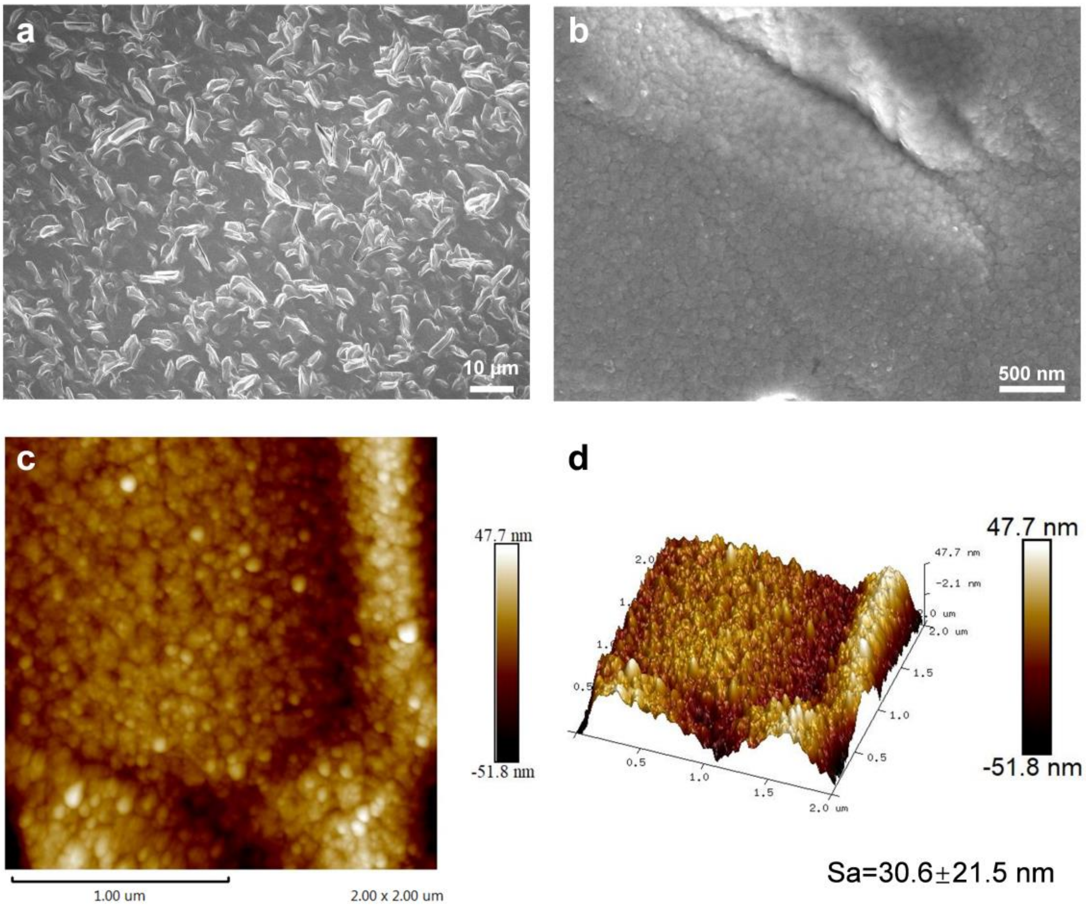

Supplementary Fig. 20 The surface morphology of Cu deposited on QCM's crystal oscillator. (**a-b**) SEM and (**c-d**) AFM tests for Cu film deposited on the QCM crystal oscillator. The surface of QCM sensor is highly rough compared with the smooth Cu films deposited on Si wafer. The roughness of Cu coated QCM sensor is ~30.6 nm, which is much higher than that on Cu deposited on Si wafer (~0.7nm). The non-uniform surface structure may induce the adsorption of Ta precursor on surface defect and wrinkle sites which is hard to be purged, therefore causes a slow growth rate on Cu with ABC-type ALD process with QCM measurements.

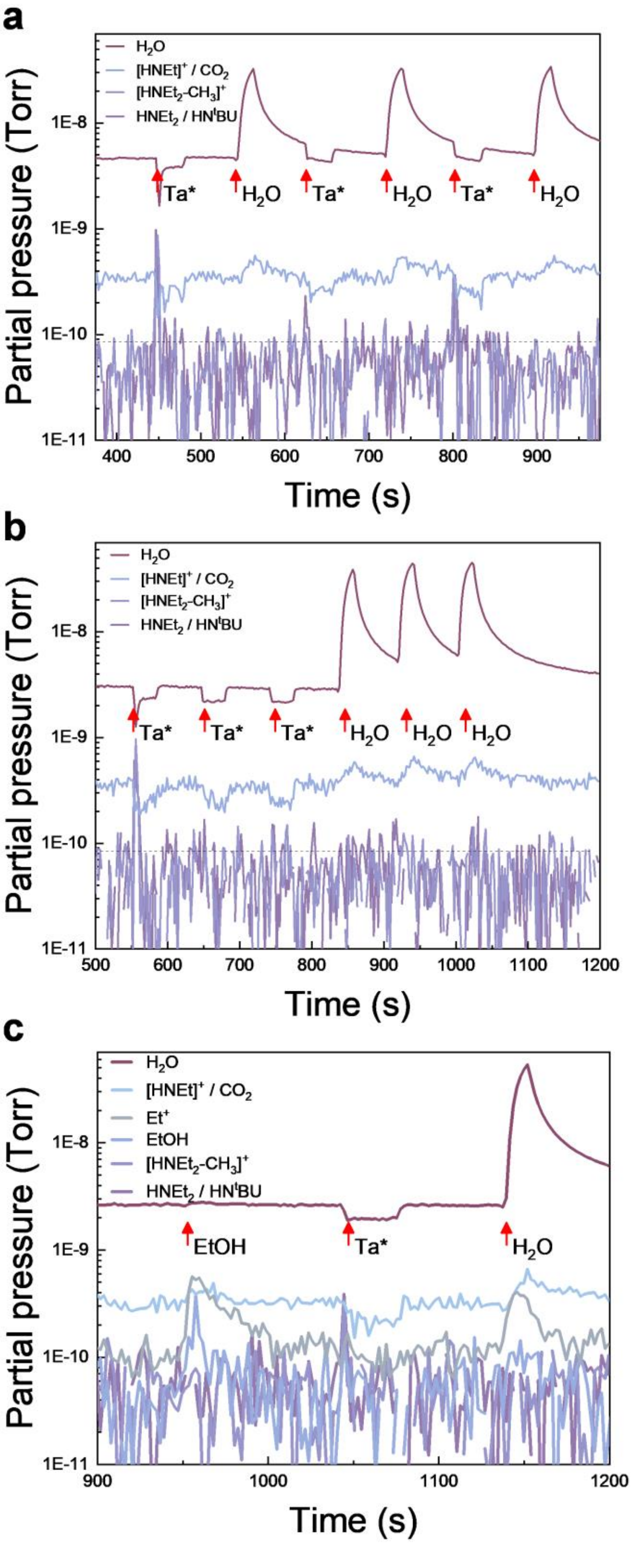

Supplementary Fig. 21 In-situ QMS measurements of the partial pressure of the by-products during precursors pulses. The precurosrs dosing sequence is (**a**) Ta precursor - H_2O - Ta precursor - H_2O- Ta precursor - H_2O; (**b**) Ta precursor - Ta precursor - Ta precursor - H_2O - H_2O - H_2O, and (**c**) EtOH - Ta precursor - H_2O. The experiments are performed at 200 ˚C.

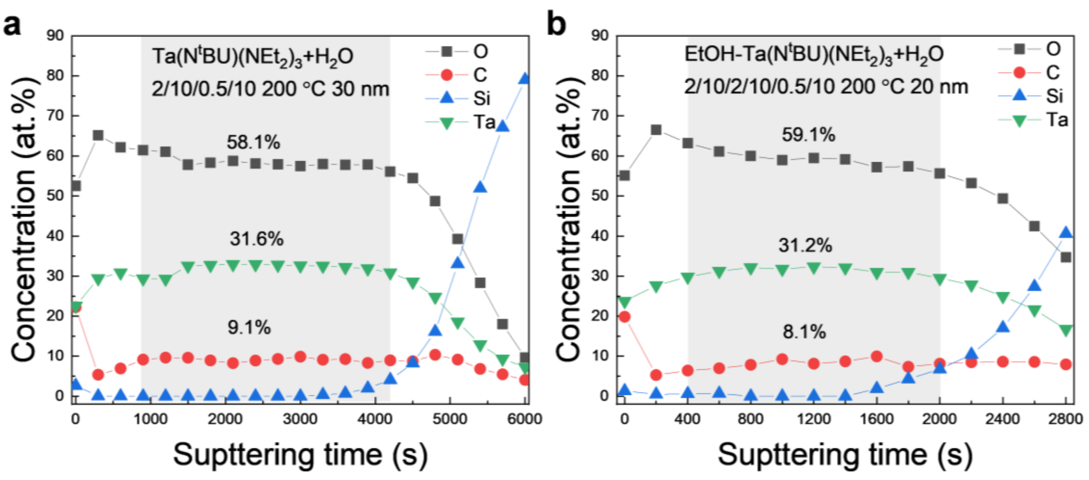

Supplementary Fig. 22. The XPS sputter depth measurements. (**a**) 30 nm-thick TaO_x film prepared by AB-type ($Ta(N^tBu)(NEt_2)_3$-H_2O) ALD and (**b**) 20 nm-thick TaO_x film prepared by ABC-type (EtOH-$Ta(N^tBu)(NEt_2)_3$-H_2O) ALD. After 300 s etching, the amount of surface C purities decreases, then reaches to a constant value. Both films contain a small amount of C composition with similar concentration. The TaO_x film fabricated with AB-type ALD contains 9.1 at. % C composition, and the ABC-type film contains 8.1 at.% C. The small amount of residual C maybe caused by the co-reactant of H_2O, which is not very active to remove all the ligands of Ta precursor. The introduction of EtOH has minimal influence to the C composition.

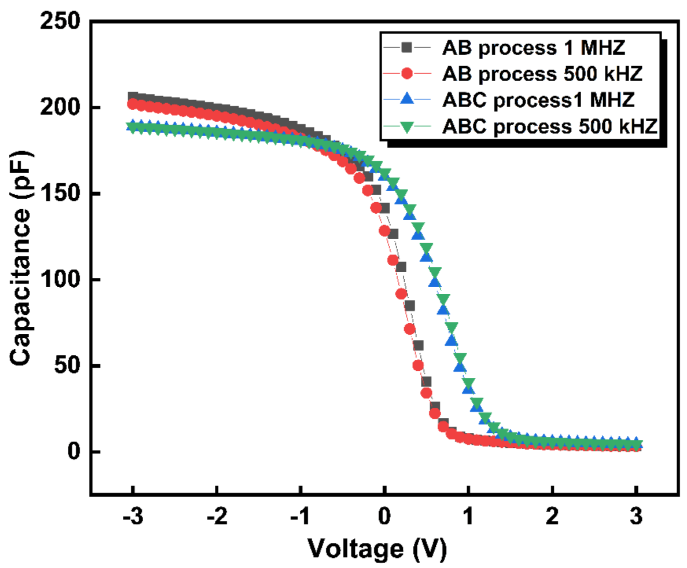

Supplementary Fig. 23 The C-V curves of TaO_x thin films. The films are fabricated with $Ta(N^tBu)(NEt_2)_3$-H_2O, EtOH-$Ta(N^tBu)(NEt_2)_3$-H_2O ALD process, respectively.

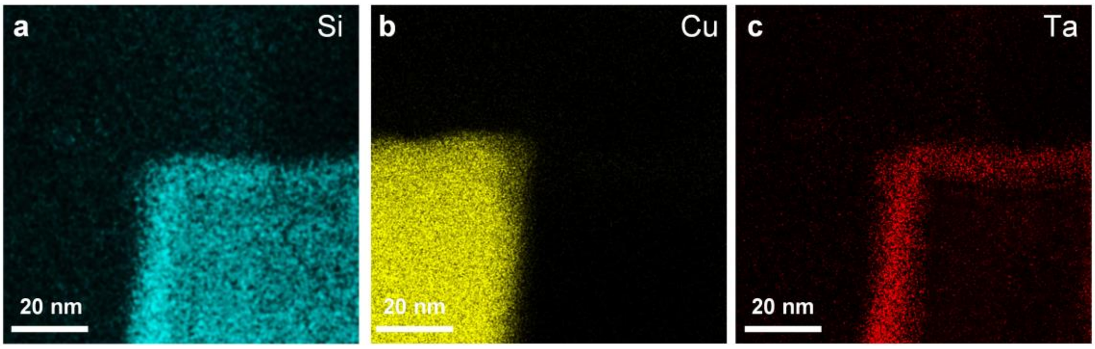

Supplementary Fig. 24 The element mappings of TaOx deposited on Cu/SiO_2 nanopatterns with ABC-type ALD at 200˚C. (**a**) Si, (**b**) Cu, and (**c**) Ta elemental signals after inherently selective ALD.

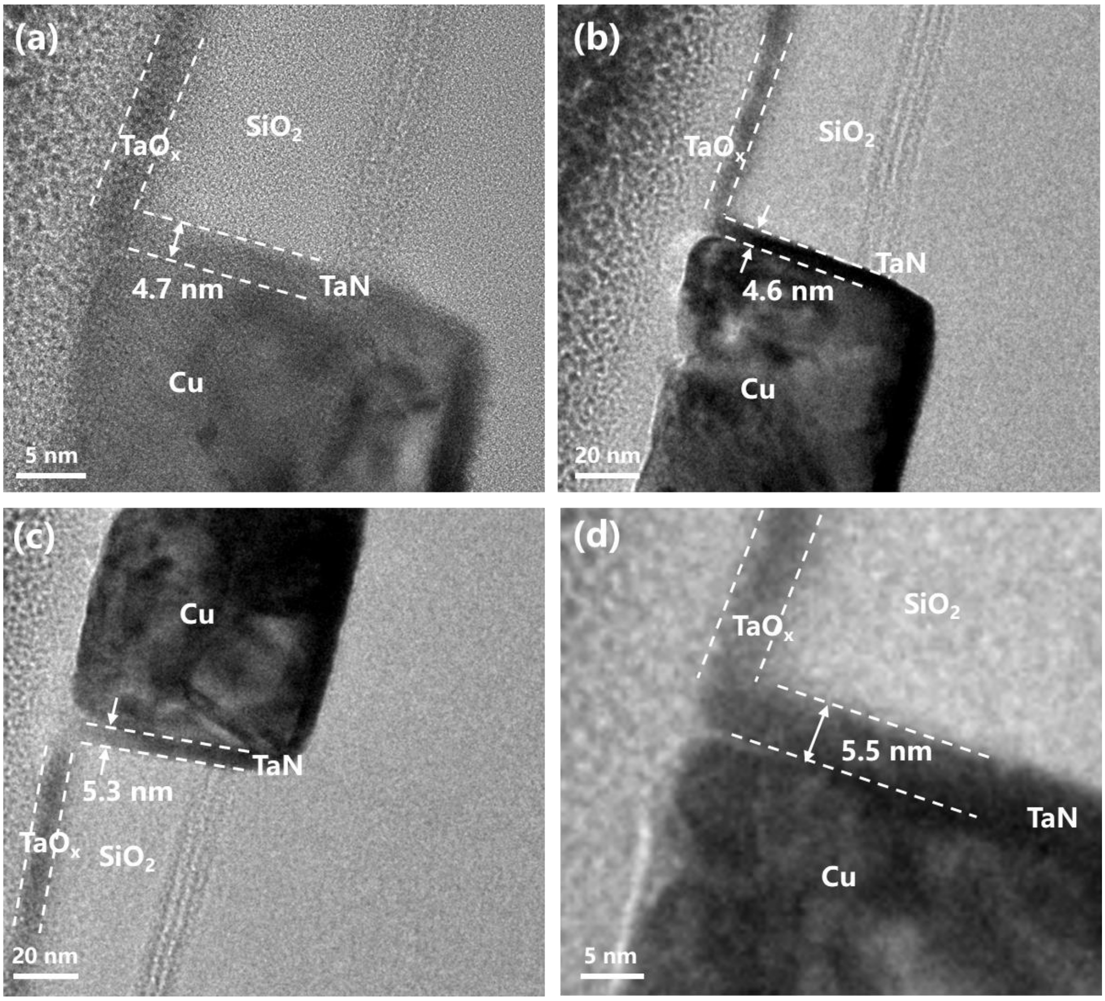

Supplementary Fig. 25 High resolution TEM images of the Cu/SiO_2 patterned structure. TEM images cover different regions of the Cu/SiO_2 substrate, TaOx deposition is exclusively localized on the TaN barrier and SiO_2. Ta atoms diffusing into neighboring Cu regions or of significant mushroom growth is not observed.

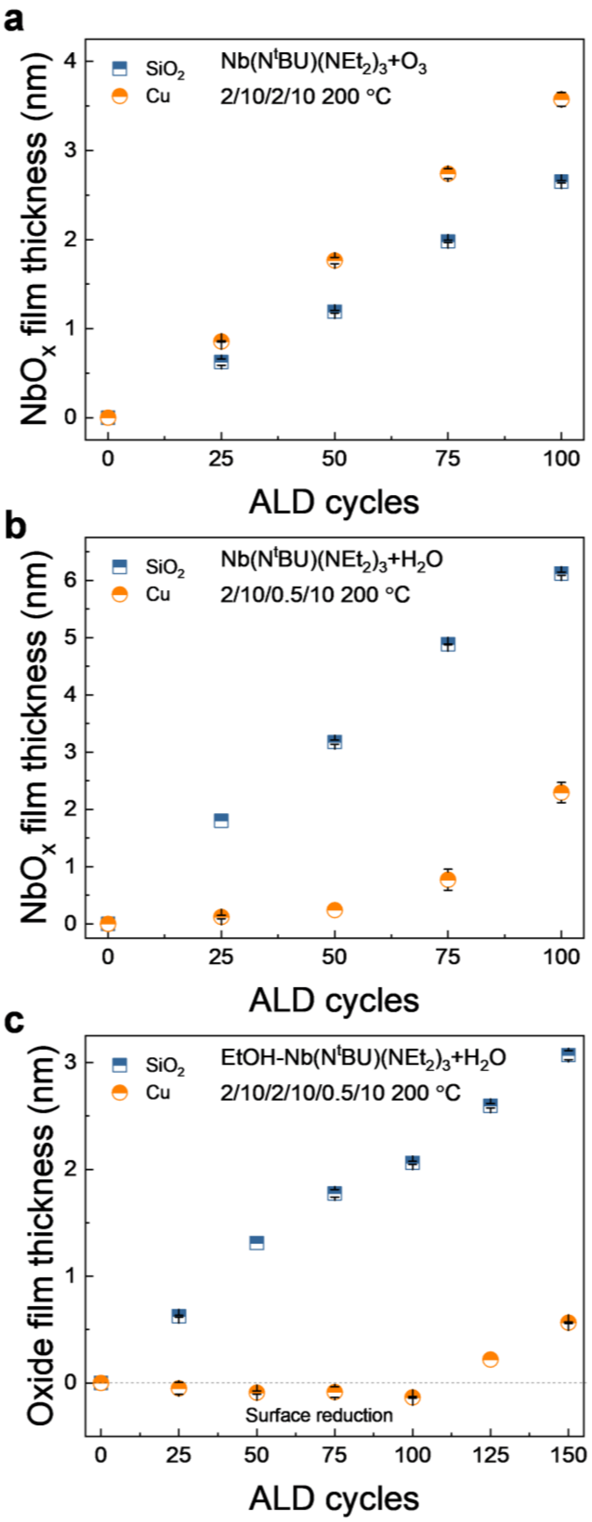

Supplementary Fig. 26 The NbO_x film thickness as a function of the ALD cycles on SiO_2 and Cu substrates. Nb(NtBu)(NEt_2)$_3$ precursor is utilized, the AB and ABC-type ALD processes are performed at 200 ˚C. The pulse sequences are (**a**) Nb(N^{t}Bu)(NEt_2)$_3$ (2 s)-O_3 (2 s); (**b**) Nb(N^{t}Bu)(NEt_2)$_3$ (2 s)-H_2O (0.5 s), and (**c**) EtOH (2 s)-Nb(N^{t}Bu)(NEt_2)$_3$ (2 s)-H_2O (0.5 s). Each data point of the film thickness is measured at two positions of one sample by spectroscopic ellipsometry. Error bars represent standard deviations after two measurements of each sample.

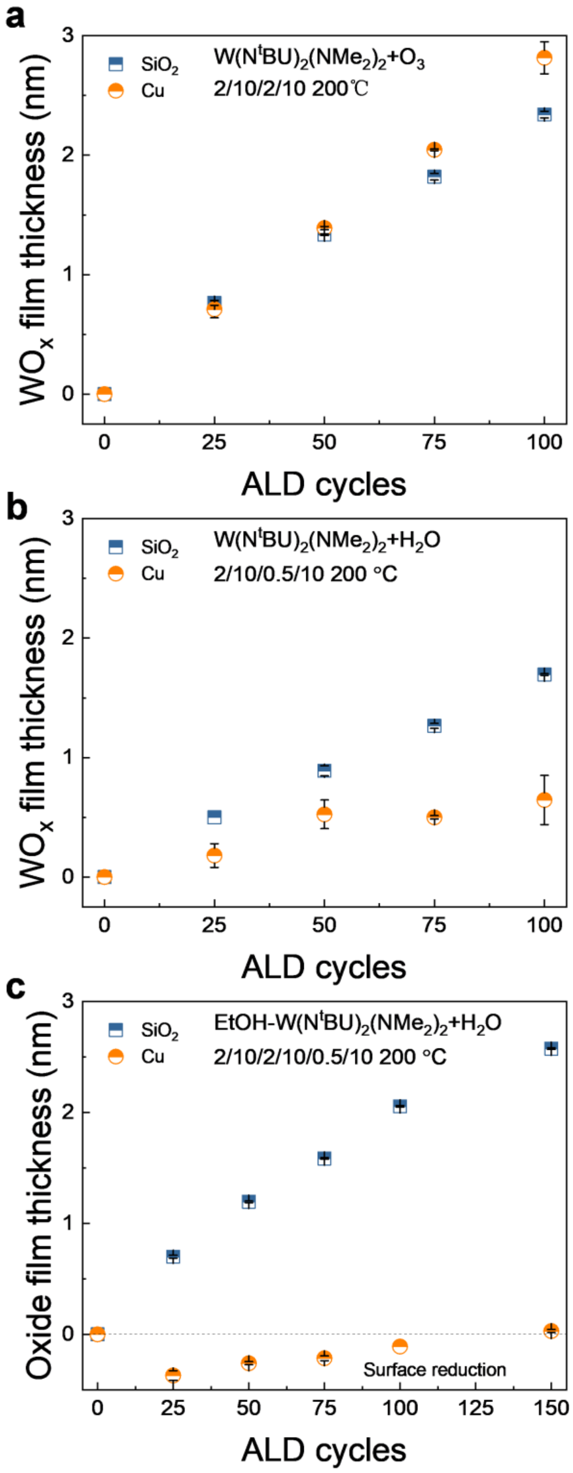

Supplementary Fig. 27 The WO_x film thickness as a function of the ALD cycles on SiO_2 and Cu substrates. $W(N^tBu)_2(NMe_2)_2$ precursor is utilized, the AB and ABC-type ALD processes are performed at 200 ˚C. The pulse sequences are (a) $W(N^tBu)_2(NMe_2)_2$ (2 s)-O_3 (2 s); (b) $W(N^tBu)_2(NMe_2)_2$ (2 s)-H_2O (0.5 s), and (c) EtOH (2 s)- $W(N^tBu)_2(NMe_2)_2$ (2 s)-H_2O (0.5 s). Each data point of the film thickness is measured at two positions of one sample by spectroscopic ellipsometry. Error bars represent standard deviations after two measurements of each sample.

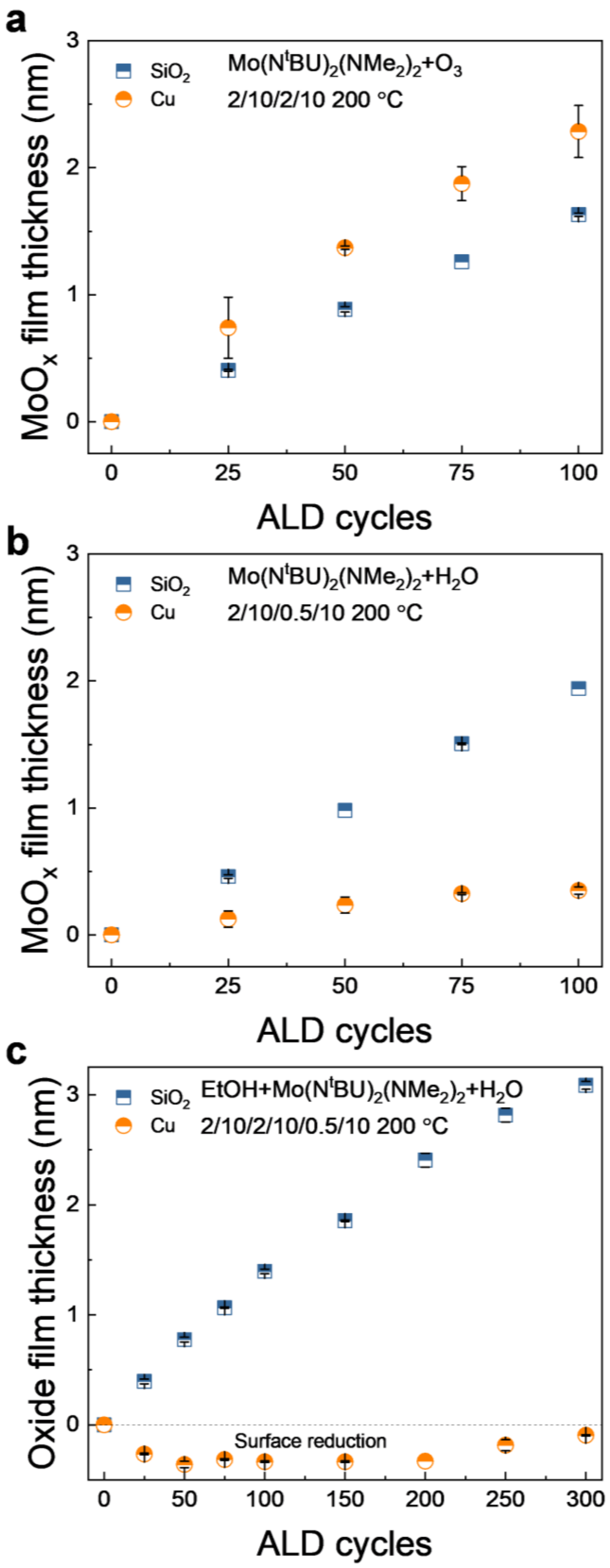

Supplementary Fig. 28 The MoO_x film thickness as a function of the ALD cycles on SiO_2 and Cu substrates. $Mo(N^tBu)_2(NMe_2)_2$ precursor is utilized, the AB and ABC-type ALD processes are performed at 200 ˚C. The pulse sequences are (a) $Mo(N^tBu)_2(NMe_2)_2$ (2 s)-O_3 (2 s); (b) $Mo(N^tBu)_2(NMe_2)_2$ (2 s)-H_2O (0.5 s), and (c) EtOH (2 s)- $Mo(N^tBu)_2(NMe_2)_2$ (2 s)-H_2O (0.5 s). Each data point of the film thickness is

measured at two positions of one sample by spectroscopic ellipsometry. Error bars represent standard deviations after two measurements of each sample.

Supplementary Table 3. Calculated energy for different reaction paths on Cu and SiO_2 substrate.

Surface		SiO_2			Cu	
Precursor	reaction	Eb (eV)	DeltaE (eV)	reaction	Eb (eV)	DeltaE (eV)
	pre_ads	0.00	-0.75	pre_ads	0.00	-1.97
Ta (N^tBu)(NEt_2)$_3$	H_trans1	0.37	-0.66	dec1	1.29	-0.76
	HNEt2_des1	0.22	-0.60	dec2	2.26	1.92
	pre_ads	0.00	-0.50	pre_ads	0.00	-2.36
	H_trans1	0.33	-0.79	dec1	0.70	0.12
Mo(NMe_2)$_2$(N^tBu)$_2$	HNEt2_des2	0.46	0.46	dec2	1.43	0.96
	H_trans1	0.46	-0.46			
	HNEt2_des2	0.80	0.80			
	pre_ads	0.00	-0.39	pre_ads	0.00	-2.15
	H_trans1	0.37	-0.69	dec1	0.79	0.27
W(N^tBu)$_2$(NMe_2)$_2$	HNEt2_des2	0.00	0.41	dec2	1.56	1.06
	H_trans1	0.59	-0.56			
	HNEt2_des2	0.74	0.74			
	pre_ads	0.00	-0.97	pre_ads	0.00	-2.01
Nb(N^tBu)(NEt_2)$_3$	H_trans1	0.03	-0.42	dec1	1.36	-0.71
	HNEt2_des1	0.00	-0.07	dec2	2.14	1.82

Supplementary references

1. Stevens, E. *et al.* Area-selective atomic layer deposition of TiN, TiO_2, and HfO_2 on silicon nitride with inhibition on amorphous carbon. *Chem. Mater.* **30**, 3223-3232(2018).
2. N. Anderson, S. Saha, G. Jursich, C. G. Takoudis, Optimization of substrate-selective atomic layer deposition of zirconia on electroplated copper using ethanol as both precursor reactant and surface pre-deposition treatment. *J. Mater. Sci.: Mater. Electron.* **32**, 5442-5456 (2021).
3. X. Yu *et al.*, Area-selective molecular layer deposition of a silicon oxycarbide low-k dielectric. *Chem. Mater.* **33**, 902-909 (2021).
4. D. Bobb-Semple, K. L. Nardi, N. Draeger, D. M. Hausmann, S. F. Bent, Area-selective atomic layer deposition assisted by self-assembled monolayers: a comparison of Cu, Co, W, and Ru. *Chem. Mater.* **31**, 1635-1645 (2019).
5. Taewon Suh *et al.*, Competitive adsorption as a route to area-selective deposition. *ACS Appl. Mater. Interfaces* **12**, 9989−9999 (2020).
6. T. L. Liu, K. L. Nardi, N. Draeger, D. M. Hausmann, S. F. Bent, Effect of multilayer versus monolayer dodecanethiol on selectivity and pattern integrity in area-selective atomic layer deposition. *ACS Appl. Mater. Interfaces* **12**, 42226-42235 (2020).
7. M. Pasquali *et al.*, Understanding selectivity loss mechanisms in selective material deposition by area deactivation on 10 nm Cu/SiO_2 patterns. *ACS Appl. Electron. Mater.* **4**, 1703-1714 (2022).
8. F. S. Minaye Hashemi, B. R. Birchansky, S. F. Bent, Selective deposition of dielectrics: limits and advantages of alkanethiol blocking agents on metal-dielectric patterns. *ACS Appl. Mater. Interfaces* **8**, 33264-33272 (2016).

9. L. Lecordier, S. Herregods, S. Armini, Vapor-deposited octadecanethiol masking layer on copper to enable area selective Hf_3N_4 atomic layer deposition on dielectrics studied by in situ spectroscopic ellipsometry. *J. Vac. Sci. Technol. A* **36**, 031605 (2018).
10. Q. Tao, K. Overhage, G. Jursich, C. Takoudis, On the initial growth of atomic layer deposited TiO_2 films on silicon and copper surfaces. *Thin Solid Films* **520**, 6752-6756 (2012).
11. J. Lee, J. M. Lee, J. H. Ahn, T. J. Park, W. H. Kim, Area-selective atomic layer deposition using vapor dosing of short-chain alkanethiol inhibitors on metal/dielectric surfaces. *Adv. Mater. Interfaces* **9**, 2102364 (2022).
12. F. S. M. Hashemi, S. F. Bent, Sequential regeneration of self-assembled monolayers for highly selective atomic layer deposition. *Adv. Mater. Interfaces* **3**, 1600464 (2016).
