# Supplementary material for: Self-aligned patterning of tantalum oxide on Cu/SiO2 through redox-coupled inherently selective atomic layer deposition
Source: Nat Commun. 2023 Jul 26;14:4493. doi: 10.1038/s41467-023-40249-2 (PMC10372027; doi:10.1038/s41467-023-40249-2)
Supplement: Supplementary file 4 — DFT configurations [file 41467_2023_40249_MOESM4_ESM.docx]

Configuration of Ta(NtBu)(NEt2)3 molecular

1.00000000000000

15.0000000000000000 0.0000000000000000 0.0000000000000000

0.0000000000000000 15.0000000000000000 0.0000000000000000

0.0000000000000000 0.0000000000000000 15.0000000000000000

Ta N C H

1 4 16 39

Selective dynamics

Direct

0.4800361009475954 0.5494764551632912 0.3653572370329336 T T T

0.4410984492142160 0.6574906200159281 0.2938874237257709 T T T

0.3927498501510521 0.4478838137057735 0.3773569245501239 T T T

0.5818952033773059 0.5009025307742047 0.3253184847293245 T T T

0.5116070954677913 0.6021647616224308 0.4847347599290597 T T T

0.6632141958607873 0.4668605400568404 0.2875697146530668 T T T

0.7255085021052888 0.5440443461012652 0.2611730324480346 T T T

0.7099673222613603 0.4074164835210423 0.3571735858344855 T T T

0.6406192239334871 0.4107072247623092 0.2045022174131647 T T T

0.5074469702919150 0.7270470559269450 0.2767908175636979 T T T

0.5508882859205604 0.7253032532247661 0.1844421873312411 T T T

0.3492039696991831 0.6797863737126556 0.2718359990980673 T T T

0.3283554326402921 0.6889391461357881 0.1722344408190099 T T T

0.4172513466883870 0.3539630635568931 0.3881865637104782 T T T

0.3726460465457578 0.2871827728525189 0.3250854165959758 T T T

0.3020905265041426 0.4716882646278253 0.4040886079278416 T T T

0.2293917379993970 0.4574694866798705 0.3336507863961027 T T T

0.5999163488042136 0.6104142467282331 0.5247182847888646 T T T

0.6120597917613662 0.5639463491006506 0.6147420381054894 T T T

0.4397341251140390 0.6469841152842696 0.5333983657562812 T T T

0.3885307741716442 0.5887052040206641 0.6000371444544230 T T T

0.7889634705528126 0.5191340328277300 0.2338679985853169 T T T

0.7403456756105442 0.5858555142176113 0.3195644813020369 T T T

0.6937190758704097 0.5861379512159047 0.2103866743424267 T T T

0.7720286258253163 0.3789762678106116 0.3301417214811629 T T T

0.6658896340356706 0.3524408866783698 0.3774842401326169 T T T

0.7265146722338445 0.4464107556995217 0.4170309535390478 T T T

0.6064895964203711 0.4516123167325460 0.1541185427084861 T T T

0.5962097716357088 0.3552327435685883 0.2226277319227003 T T T

0.7015971083027144 0.3828862591672373 0.1745012216082786 T T T

0.5607319169430783 0.7208354040038115 0.3273252045708827 T T T

0.4764202066231783 0.7930839212305197 0.2889621430322548 T T T

0.5027789540266804 0.7387897052170197 0.1309948604339126 T T T

0.5802720970703921 0.6593458247054461 0.1722089291316538 T T T

0.6043866787897499 0.7754551022313231 0.1801665707048626 T T T

0.3299758300837879 0.7425241755193631 0.3062234811150824 T T T

0.3055831417265182 0.6275474018913202 0.2997762926175272 T T T

0.2560743099904362 0.6959833059111575 0.1614977901328728 T T T

0.3519704617411725 0.6298682402661995 0.1357257096957217 T T T

0.3607015569800905 0.7480244912788303 0.1434586833060966 T T T

0.4037447792077167 0.3338272390220935 0.4581554655475600 T T T

0.4898464457253078 0.3499482295607557 0.3791714490205560 T T T

0.4043201309289176 0.2213754020569807 0.3325978630649101 T T T

0.3798872110591004 0.3083784313084810 0.2552599389703949 T T T

0.3013227070325649 0.2783409800619108 0.3391627406187485 T T T

0.3012643785341407 0.5433380147339960 0.4226573420526502 T T T

0.2838193577978913 0.4356873670609289 0.4660772956878640 T T T

0.2209145835473689 0.3867570547768805 0.3172456995873488 T T T

0.2458416040777935 0.4930189110435528 0.2716758622663217 T T T

0.1650178214535137 0.4827203968720725 0.3583137151752809 T T T

0.6163772805809186 0.6822336590076821 0.5324610746173252 T T T

0.6480290351998862 0.5829504024833438 0.4767935326048118 T T T

0.6822437825001751 0.5674817015504360 0.6359028244961276 T T T

0.5927769233313976 0.4933485305302600 0.6098950190408050 T T T

0.5717778533166911 0.5953048645322943 0.6673434122813683 T T T

0.3914256469709122 0.6742060025091609 0.4844899056465314 T T T

0.4674155770784847 0.7060582280392346 0.5680172958869496 T T T

0.3333678459583603 0.6267363784169678 0.6301432918043697 T T T

0.4315414901355481 0.5643435840734401 0.6539611700024975 T T T

0.3602514616410805 0.5302342146122712 0.5661878384011628 T T T

Configuration of Cu substrate

1.00000000000000

15.1407000000000007 0.0000000000000000 0.0000000000000000

-7.5703500000000004 13.1122308309999998 0.0000000000000000

0.0000000000000000 0.0000000000000000 26.1812000000000005

Cu

108

Selective dynamics

Direct

0.2222200000000001 0.1111099999999965 0.1964400000000026 F F F

0.1125329567016909 0.2244337200783566 0.2758013164826853 T T T

0.0022462654813935 0.3366284404939657 0.3549853995428703 T T T

0.0555599999999998 0.1111099999999965 0.1964400000000026 F F F

0.2788890563495376 0.2243222450195725 0.2757893432246674 T T T

0.1692006259451520 0.3372613201804163 0.3552663145611555 T T T

0.0555599999999998 0.2777799999999999 0.1964400000000026 F F F

0.2784949949631557 0.0573832358088929 0.2756765261181743 T T T

0.1689874130801276 0.1704401633026507 0.3549521271266570 T T T

0.2222200000000001 0.2777799999999999 0.1964400000000026 F F F

0.1121115565549027 0.0571801431096010 0.2756747038800930 T T T

0.0024165125301479 0.1702640511930722 0.3549910960025365 T T T

0.5555599999999998 0.1111099999999965 0.1964400000000026 F F F

0.4450834744385839 0.2238419153006874 0.2756685842646340 T T T

0.3357268256798160 0.3371362782509615 0.3552338978648413 T T T

0.3888900000000035 0.1111099999999965 0.1964400000000026 F F F

0.6119339320822125 0.2234320089542085 0.2756235687420416 T T T

0.5019646351603192 0.3364045007025224 0.3549112753387177 T T T

0.3888900000000035 0.2777799999999999 0.1964400000000026 F F F

0.6120322483891051 0.0568849513489844 0.2756240924131176 T T T

0.5016615192834648 0.1693887337722555 0.3548732936868096 T T T

0.5555599999999998 0.2777799999999999 0.1964400000000026 F F F

0.4450731038908329 0.0571056103564527 0.2757026421256612 T T T

0.3352755967683166 0.1700954146477961 0.3549422942398293 T T T

0.8888900000000035 0.1111099999999965 0.1964400000000026 F F F

0.7786408464492113 0.2235100720389757 0.2756738472776361 T T T

0.6684972833705674 0.3358485246163176 0.3549066443808528 T T T

0.7222200000000001 0.1111099999999965 0.1964400000000026 F F F

0.9457822168998409 0.2239548346811829 0.2757318935564532 T T T

0.8350877369902535 0.3359628233071614 0.3549537559345126 T T T

0.7222200000000001 0.2777799999999999 0.1964400000000026 F F F

0.9458938737909671 0.0574490714474795 0.2756843218437027 T T T

0.8353086859547707 0.1695435922611984 0.3549725764833692 T T T

0.8888900000000035 0.2777799999999999 0.1964400000000026 F F F

0.7791476231345850 0.0572037896584561 0.2756990714094814 T T T

0.6683884985660106 0.1692918770004936 0.3548266292746328 T T T

0.2222200000000001 0.4444400000000002 0.1964400000000026 F F F

0.1122147486602194 0.5569268082022968 0.2757230494835681 T T T

0.0021153672746188 0.6694779913312962 0.3548071240874560 T T T

0.0555599999999998 0.4444400000000002 0.1964400000000026 F F F

0.2794457647340842 0.5577262339807457 0.2759336868347444 T T T

0.1689103643277830 0.6698455525228180 0.3547210398977725 T T T

0.0555599999999998 0.6111099999999965 0.1964400000000026 F F F

0.2793104031182561 0.3912239898520522 0.2760398213930612 T T T

0.1689623558514135 0.5035791308723895 0.3553030122693626 T T T

0.2222200000000001 0.6111099999999965 0.1964400000000026 F F F

0.1124817066884682 0.3907489628423156 0.2759270566631823 T T T

0.0021549952416792 0.5029674454398189 0.3548845319938727 T T T

0.5555599999999998 0.4444400000000002 0.1964400000000026 F F F

0.4456713872149679 0.5576049919713043 0.2759896764753993 T T T

0.3357702422210355 0.6701809846722748 0.3552578674635778 T T T

0.3888900000000035 0.4444400000000002 0.1964400000000026 F F F

0.6121937959225607 0.5568048651262413 0.2757982236245515 T T T

0.5024488756908063 0.6701695079882343 0.3551684116197690 T T T

0.3888900000000035 0.6111099999999965 0.1964400000000026 F F F

0.6120686642614660 0.3900329640791979 0.2756564076086862 T T T

0.5023751646500860 0.5033791885502988 0.3553132497185579 T T T

0.5555599999999998 0.6111099999999965 0.1964400000000026 F F F

0.4455350075795126 0.3906393849703724 0.2758260952879434 T T T

0.3358167223061379 0.5037038844135939 0.3553804688215353 T T T

0.8888900000000035 0.4444400000000002 0.1964400000000026 F F F

0.7787394960449450 0.5566726230312501 0.2757159857707450 T T T

0.6688452026711386 0.6697391368568709 0.3548410881273747 T T T

0.7222200000000001 0.4444400000000002 0.1964400000000026 F F F

0.9453504463933553 0.5566793356728018 0.2756667163890382 T T T

0.8353416806609361 0.6694865953741733 0.3549438955207177 T T T

0.7222200000000001 0.6111099999999965 0.1964400000000026 F F F

0.9453386920160878 0.3901669477844670 0.2756982901106076 T T T

0.8353123090923952 0.5026506759640369 0.3549849002556805 T T T

0.8888900000000035 0.6111099999999965 0.1964400000000026 F F F

0.7785452460315441 0.3898584479324962 0.2756752548655633 T T T

0.6686977837508951 0.5027810752612051 0.3548963243335269 T T T

0.2222200000000001 0.7777799999999999 0.1964400000000026 F F F

0.1117210713366227 0.8904908379296353 0.2756776215709487 T T T

0.0023498875030890 0.0034173765171358 0.3549908263809247 T T T

0.0555599999999998 0.7777799999999999 0.1964400000000026 F F F

0.2784684222727321 0.8905490317412745 0.2756964407431068 T T T

0.1684570554180040 0.0032867377591607 0.3549865410064216 T T T

0.0555599999999998 0.9444400000000002 0.1964400000000026 F F F

0.2788722352432583 0.7239135232666982 0.2757090769360693 T T T

0.1684290808789549 0.8363149122963132 0.3548464925793982 T T T

0.2222200000000001 0.9444400000000002 0.1964400000000026 F F F

0.1118525625329248 0.7235746189918472 0.2756509607921184 T T T

0.0020233838658152 0.8364772786276325 0.3549952250563025 T T T

0.5555599999999998 0.7777799999999999 0.1964400000000026 F F F

0.4452537676178145 0.8906552343295511 0.2756692671129082 T T T

0.3349972576380165 0.0029944483043202 0.3549438563308182 T T T

0.3888900000000035 0.7777799999999999 0.1964400000000026 F F F

0.6125518168678633 0.8906377026552779 0.2755953896412838 T T T

0.5016415717949072 0.0026115604487414 0.3548627126951117 T T T

0.3888900000000035 0.9444400000000002 0.1964400000000026 F F F

0.6124172825342419 0.7238556185077462 0.2757223533671108 T T T

0.5021193032373275 0.8364520169958911 0.3548474148648629 T T T

0.5555599999999998 0.9444400000000002 0.1964400000000026 F F F

0.4457829600251727 0.7241975843795827 0.2755927834328563 T T T

0.3352466235877447 0.8363104746590011 0.3547143703875456 T T T

0.8888900000000035 0.7777799999999999 0.1964400000000026 F F F

0.7791511222250588 0.8906365660427076 0.2757073887097951 T T T

0.6686015069553184 0.0026755571796546 0.3548208984302815 T T T

0.7222200000000001 0.7777799999999999 0.1964400000000026 F F F

0.9457341821523536 0.8905200547641192 0.2756815924230103 T T T

0.8355307358077790 0.0031394566521955 0.3549069768082376 T T T

0.7222200000000001 0.9444400000000002 0.1964400000000026 F F F

0.9453002050791433 0.7235369875202265 0.2756322062003250 T T T

0.8355287072670213 0.8364451241161692 0.3548927315474485 T T T

0.8888900000000035 0.9444400000000002 0.1964400000000026 F F F

0.7788688799244259 0.7235324384022707 0.2757416631579745 T T T

0.6688678677355688 0.8363427313266668 0.3547627092614710 T T T

Configuration of precursor adsorbing on Cu substrate

1.00000000000000

15.1407000000000007 0.0000000000000000 0.0000000000000000

-7.5703500000000004 13.1122308309999998 0.0000000000000000

0.0000000000000000 0.0000000000000000 26.1812000000000005

Cu H Ta N C

108 39 1 4 16

Selective dynamics

Direct

0.2222200000000001 0.1111099999999965 0.1964400000000026 F F F

0.1111938184345358 0.2226025138203058 0.2756201086445434 T T T

-0.0006609588720054 0.3338062477362012 0.3550786674411172 T T T

0.0555599999999998 0.1111099999999965 0.1964400000000026 F F F

0.2770225416031729 0.2215358032477128 0.2751563989504151 T T T

0.1655081371875662 0.3330811225154803 0.3546447669044581 T T T

0.0555599999999998 0.2777799999999999 0.1964400000000026 F F F

0.2770926386265032 0.0547935853148488 0.2757101009106465 T T T

0.1658909852986283 0.1667215377841498 0.3549625897078640 T T T

0.2222200000000001 0.2777799999999999 0.1964400000000026 F F F

0.1107022343122058 0.0554275355714265 0.2757287663637712 T T T

-0.0004488515594389 0.1673785578794913 0.3552223393150261 T T T

0.5555599999999998 0.1111099999999965 0.1964400000000026 F F F

0.4437146479191061 0.2216757881264279 0.2754725555927496 T T T

0.3323068155469569 0.3316389505195595 0.3526742249663419 T T T

0.3888900000000035 0.1111099999999965 0.1964400000000026 F F F

0.6107225698943193 0.2224452854685887 0.2756670472022172 T T T

0.5000541936355036 0.3328068457019597 0.3537895797051500 T T T

0.3888900000000035 0.2777799999999999 0.1964400000000026 F F F

0.6105778790617803 0.0557931155166250 0.2754265535576533 T T T

0.4996604895600992 0.1666809839993409 0.3548861784080765 T T T

0.5555599999999998 0.2777799999999999 0.1964400000000026 F F F

0.4438602342638951 0.0553328168131725 0.2756745852636566 T T T

0.3324945692740306 0.1658276655456774 0.3546307155114182 T T T

0.8888900000000035 0.1111099999999965 0.1964400000000026 F F F

0.7776389597596272 0.2227030444751870 0.2756592311547640 T T T

0.6663273448786829 0.3338290171912529 0.3548992988627224 T T T

0.7222200000000001 0.1111099999999965 0.1964400000000026 F F F

0.9445566152843121 0.2227619354690674 0.2757219465671399 T T T

0.8331241614837535 0.3342533033099524 0.3549285163779149 T T T

0.7222200000000001 0.2777799999999999 0.1964400000000026 F F F

0.9447645835871206 0.0564034664332304 0.2756366120516092 T T T

0.8331086882065635 0.1676946913901806 0.3549403852475986 T T T

0.8888900000000035 0.2777799999999999 0.1964400000000026 F F F

0.7780920389318499 0.0562235959437737 0.2754395700634161 T T T

0.6664030799422965 0.1673236394031216 0.3548916459588766 T T T

0.2222200000000001 0.4444400000000002 0.1964400000000026 F F F

0.1111259318221971 0.5559258052236348 0.2746690768341173 T T T

-0.0009508769824453 0.6675286266524728 0.3543997962234605 T T T

0.0555599999999998 0.4444400000000002 0.1964400000000026 F F F

0.2789685557301910 0.5564789378726424 0.2776965557051159 T T T

0.1651779760787637 0.6678233558500768 0.3520450967723073 T T T

0.0555599999999998 0.6111099999999965 0.1964400000000026 F F F

0.2779874349242664 0.3903593591397021 0.2766848688901220 T T T

0.1647335297541051 0.4986337536588243 0.3528761924644723 T T T

0.2222200000000001 0.6111099999999965 0.1964400000000026 F F F

0.1109652013846421 0.3889003341094363 0.2750694431066387 T T T

-0.0012787143075773 0.5000539518193194 0.3537182619958334 T T T

0.5555599999999998 0.4444400000000002 0.1964400000000026 F F F

0.4433582253088777 0.5556499946442759 0.2773301614102988 T T T

0.3327139955189757 0.6668619732337292 0.3602562676654163 T T T

0.3888900000000035 0.4444400000000002 0.1964400000000026 F F F

0.6110289672578700 0.5557726011648166 0.2751926143856373 T T T

0.4998636959796088 0.6676327022356859 0.3585437756052427 T T T

0.3888900000000035 0.6111099999999965 0.1964400000000026 F F F

0.6111872628469196 0.3894193024289336 0.2755333233332613 T T T

0.5014917439709031 0.5004629912283380 0.3530971672679833 T T T

0.5555599999999998 0.6111099999999965 0.1964400000000026 F F F

0.4439817749201139 0.3887669283025903 0.2749390186323487 T T T

0.3339322874310600 0.5009820318822823 0.3601153922731825 T T T

0.8888900000000035 0.4444400000000002 0.1964400000000026 F F F

0.7774793883863166 0.5562545879791170 0.2757199254941762 T T T

0.6665410043869983 0.6667358842063832 0.3549888436884441 T T T

0.7222200000000001 0.4444400000000002 0.1964400000000026 F F F

0.9441850805642920 0.5564278134751884 0.2754649316937194 T T T

0.8327786336778792 0.6673307271668647 0.3553121058915480 T T T

0.7222200000000001 0.6111099999999965 0.1964400000000026 F F F

0.9443652816988010 0.3890100338962559 0.2754677397971460 T T T

0.8329879628857414 0.5007615408035924 0.3549146546980272 T T T

0.8888900000000035 0.6111099999999965 0.1964400000000026 F F F

0.7777709454602900 0.3895207419005588 0.2756485970012366 T T T

0.6665403738434024 0.5002719445685035 0.3550899824956371 T T T

0.2222200000000001 0.7777799999999999 0.1964400000000026 F F F

0.1105686503488447 0.8890236009266035 0.2756559153189957 T T T

-0.0006121072484931 0.0006534449319523 0.3549851751887497 T T T

0.0555599999999998 0.7777799999999999 0.1964400000000026 F F F

0.2773614107075328 0.8886243543181357 0.2754539960081830 T T T

0.1660040944232899 0.0000392301572911 0.3551193777287172 T T T

0.0555599999999998 0.9444400000000002 0.1964400000000026 F F F

0.2788307100858994 0.7219221665141418 0.2764665107263329 T T T

0.1662842915915105 0.8339720073402908 0.3547838924857280 T T T

0.2222200000000001 0.9444400000000002 0.1964400000000026 F F F

0.1108038282930092 0.7229029429258654 0.2749154271961335 T T T

-0.0006529050392294 0.8338995449491332 0.3550800179182336 T T T

0.5555599999999998 0.7777799999999999 0.1964400000000026 F F F

0.4442350979027217 0.8889778657521147 0.2749599114549722 T T T

0.3328856352322550 0.0002224798978490 0.3552552177146346 T T T

0.3888900000000035 0.7777799999999999 0.1964400000000026 F F F

0.6111254836296149 0.8889786826500655 0.2747339770292703 T T T

0.4999363801134830 0.0007886817382215 0.3548718703447094 T T T

0.3888900000000035 0.9444400000000002 0.1964400000000026 F F F

0.6097579745772500 0.7213877976731794 0.2763507631699945 T T T

0.4997848279216289 0.8350950585679762 0.3528041153960176 T T T

0.5555599999999998 0.9444400000000002 0.1964400000000026 F F F

0.4437577293337611 0.7211603284235796 0.2773676688035477 T T T

0.3334941244865964 0.8350183125931335 0.3540983483456374 T T T

0.8888900000000035 0.7777799999999999 0.1964400000000026 F F F

0.7780402257833552 0.8894347586303099 0.2750807361261886 T T T

0.6667362816167188 0.0016243243373873 0.3536362794217703 T T T

0.7222200000000001 0.7777799999999999 0.1964400000000026 F F F

0.9442027242734754 0.8892936411094523 0.2757297623128869 T T T

0.8335500111685078 0.0013507870352483 0.3544859721638458 T T T

0.7222200000000001 0.9444400000000002 0.1964400000000026 F F F

0.9437077594432882 0.7229067229907218 0.2756600044858875 T T T

0.8329001738999124 0.8338556079542085 0.3549702739398063 T T T

0.8888900000000035 0.9444400000000002 0.1964400000000026 F F F

0.7772137238665666 0.7226062134575330 0.2757214335154412 T T T

0.6680081441666849 0.8347807537896982 0.3525291671249112 T T T

0.2730149381565605 0.5766324242037416 0.6678383007062382 T T T

0.2239845568794386 0.4959548299852772 0.6132852369854258 T T T

0.2387739951330078 0.6204141656104935 0.6121832035805198 T T T

0.4293904439722415 0.5589284611704376 0.6687392189127610 T T T

0.5072703646693090 0.5875954283335898 0.6132297056843358 T T T

0.3836173678641834 0.4766784933436610 0.6145949664473406 T T T

0.4136771365942362 0.7767037498204420 0.6094506397079763 T T T

0.5252011462047846 0.7651471449466124 0.6101907895052122 T T T

0.4469504241411889 0.7339746976523255 0.6657646792537277 T T T

0.1524658344240323 0.5467392952449734 0.4336930664691989 T T T

0.1523834763753228 0.6617800383320068 0.4424061506267060 T T T

0.1354165818135748 0.6326224181017556 0.5375025211087497 T T T

0.1530326392632146 0.5262559355385278 0.5306767474619704 T T T

0.0428066105483422 0.5227409499126464 0.5041546447673445 T T T

0.3396614358165292 0.8193862835397934 0.4372454905551946 T T T

0.4216207248047962 0.8043078469356540 0.4783896149704315 T T T

0.3468600186058517 0.8912998126086552 0.5227550530956945 T T T

0.2968117059760595 0.7690654915054106 0.5520524266682941 T T T

0.2206174620508491 0.7941601551366343 0.5088339788823848 T T T

0.6468579859943999 0.6758554257508677 0.4465916659030681 T T T

0.5486136785801479 0.5910646056827015 0.4868131302533217 T T T

0.7055772415391115 0.6674876367567226 0.5345583618636645 T T T

0.6295191800626678 0.7158438554423190 0.5610410583064038 T T T

0.7344276708605756 0.7934840931475619 0.5199453679688554 T T T

0.5541680830233170 0.8525757447181422 0.4350181337206432 T T T

0.6699636482264669 0.8539198485581394 0.4434293257502691 T T T

0.6640081772880156 0.8841451996656450 0.5382336084983008 T T T

0.5421412628841038 0.8716987112233159 0.5309261641608441 T T T

0.6500851925354695 0.9742933818503132 0.5006873639001280 T T T

0.1975798036781544 0.3536512960429491 0.4403779214549425 T T T

0.1988239364703223 0.4548522099479573 0.4726571969651777 T T T

0.1229929028263327 0.3109809081807577 0.5288996455833502 T T T

0.2459660710850461 0.3935184519292168 0.5554114170343423 T T T

0.2220054759704657 0.2823972285743671 0.5205442958931777 T T T

0.4716283423314862 0.4674592431793723 0.4370348907739423 T T T

0.3592569413771978 0.3477978203441732 0.4394216121011097 T T T

0.4934695536271931 0.3741460919379584 0.5039915688519928 T T T

0.3753378815406186 0.3399643909783499 0.5339871220496454 T T T

0.4755258544942504 0.4682488398807962 0.5357981488426491 T T T

0.3902503593789625 0.6204605204769283 0.4814187904292496 T T T

0.2904557265899263 0.6675568161011761 0.4596596399611028 T T T

0.5398818171888784 0.7198471174089570 0.4669811946765019 T T T

0.3817443097439707 0.6165009803785857 0.5501602416489114 T T T

0.3429716240595714 0.4722071151463618 0.4614306859394115 T T T

0.3822139054277343 0.6196245052575564 0.6052331101342252 T T T

0.2727528684909448 0.5753341735307061 0.6257923691500473 T T T

0.4284123727872681 0.5568175215043571 0.6266980803953547 T T T

0.4461246057420923 0.7306884328404878 0.6237575580386518 T T T

0.1791314056946657 0.6125585296257409 0.4601578863694868 T T T

0.1250031888152549 0.5717686800981734 0.5112484217377696 T T T

0.3395787446872965 0.7763440292729685 0.4720111983840056 T T T

0.2980255609460912 0.8087681777108678 0.5167276159095177 T T T

0.6012595151249107 0.6732630055524567 0.4805961914785261 T T T

0.6715624189152472 0.7155985023446985 0.5268522887397314 T T T

0.5957181221004296 0.8304424692584130 0.4624384107532652 T T T

0.6140689573022809 0.8931945063075194 0.5110601192982831 T T T

0.2343464753510986 0.4066496954332363 0.4732063049540448 T T T

0.2054503469984411 0.3450337383195288 0.5225458259672112 T T T

0.4028258391081758 0.4208963617481493 0.4607942259783189 T T T

0.4382497678491706 0.3995803013776398 0.5117808689631990 T T T

Final configuration of precursor decomposing the first ligand on the Cu substrate

Cu H Ta N C

1.00000000000000

15.1407000000000007 0.0000000000000000 0.0000000000000000

-7.5703500000000004 13.1122308309999998 0.0000000000000000

0.0000000000000000 0.0000000000000000 26.1812000000000005

Cu H Ta N C

108 39 1 4 16

Selective dynamics

Direct

0.2222200000000001 0.1111099999999965 0.1964400000000026 F F F

0.1114543220718431 0.2200697516272783 0.2761788910732861 T T T

0.9980787910695810 0.3321484862723310 0.3547304202818751 T T T

0.0555599999999998 0.1111099999999965 0.1964400000000026 F F F

0.2774102849755309 0.2214456124541186 0.2764268504037588 T T T

0.1628616135824075 0.3349584331936057 0.3522326396340887 T T T

0.0555599999999998 0.2777799999999999 0.1964400000000026 F F F

0.2766757539955904 0.0536380285334062 0.2747282862779272 T T T

0.1671559196378567 0.1586126555646032 0.3565731203644802 T T T

0.2222200000000001 0.2777799999999999 0.1964400000000026 F F F

0.1114325012037654 0.0550120832342316 0.2764813789939353 T T T

0.9978289326684617 0.1638218521852792 0.3536136602956717 T T T

0.5555599999999998 0.1111099999999965 0.1964400000000026 F F F

0.4448146883076163 0.2221842850551710 0.2748372338603460 T T T

0.3273911632951183 0.3307256757981989 0.3626624344099625 T T T

0.3888900000000035 0.1111099999999965 0.1964400000000026 F F F

0.6116414964814680 0.2220251111762650 0.2754495811719551 T T T

0.4997601719497737 0.3318978907228569 0.3543464498823923 T T T

0.3888900000000035 0.2777799999999999 0.1964400000000026 F F F

0.6119451648564436 0.0553583790243621 0.2754132354733159 T T T

0.5020639227053039 0.1654184865724631 0.3544695326535461 T T T

0.5555599999999998 0.2777799999999999 0.1964400000000026 F F F

0.4446944197575888 0.0552789052791809 0.2754535543786290 T T T

0.3369895487394245 0.1622380415072939 0.3512209555116712 T T T

0.8888900000000035 0.1111099999999965 0.1964400000000026 F F F

0.7780830743702509 0.2216646179333921 0.2757231220526464 T T T

0.6661357269571155 0.3320122542432173 0.3552741638377270 T T T

0.7222200000000001 0.1111099999999965 0.1964400000000026 F F F

0.9444988945026083 0.2215590856681260 0.2753997072493064 T T T

0.8325457121641193 0.3321994501304317 0.3550452660625183 T T T

0.7222200000000001 0.2777799999999999 0.1964400000000026 F F F

0.9441988427317227 0.0536919837538008 0.2756399959664577 T T T

0.8326062529606872 0.1648058729498132 0.3552476778260996 T T T

0.8888900000000035 0.2777799999999999 0.1964400000000026 F F F

0.7782501447065044 0.0546390579816673 0.2762621813121129 T T T

0.6668575302558608 0.1655357249171772 0.3549914939880381 T T T

0.2222200000000001 0.4444400000000002 0.1964400000000026 F F F

0.1114251243671731 0.5552073266990192 0.2757816278761211 T T T

0.9997083184780777 0.6647967900333782 0.3552181844967990 T T T

0.0555599999999998 0.4444400000000002 0.1964400000000026 F F F

0.2778935133083564 0.5555456199972895 0.2769311847282694 T T T

0.1670616987361484 0.6655851099819969 0.3554526283905943 T T T

0.0555599999999998 0.6111099999999965 0.1964400000000026 F F F

0.2775072973700361 0.3868903124964973 0.2766543352395558 T T T

0.1666645045187778 0.5005290444797126 0.3555457935234259 T T T

0.2222200000000001 0.6111099999999965 0.1964400000000026 F F F

0.1105562402016886 0.3883009320840358 0.2750094882050149 T T T

0.9996498294631593 0.4989465483989832 0.3549428535144585 T T T

0.5555599999999998 0.4444400000000002 0.1964400000000026 F F F

0.4436963685179407 0.5544578610677675 0.2761744238133588 T T T

0.3354598074217492 0.6678446909639746 0.3592700622657219 T T T

0.3888900000000035 0.4444400000000002 0.1964400000000026 F F F

0.6100445417445712 0.5542357559107671 0.2757194735419972 T T T

0.5001153246829091 0.6666274255663254 0.3550786600693741 T T T

0.3888900000000035 0.6111099999999965 0.1964400000000026 F F F

0.6104398760547767 0.3883501905318454 0.2756140850646318 T T T

0.4979203007088344 0.4975326012471157 0.3550654043296007 T T T

0.5555599999999998 0.6111099999999965 0.1964400000000026 F F F

0.4409401447686818 0.3863636795601550 0.2777420928421961 T T T

0.3326169152739027 0.4997447077562640 0.3550225921531620 T T T

0.8888900000000035 0.4444400000000002 0.1964400000000026 F F F

0.7772109378829057 0.5547137868018552 0.2756663469198415 T T T

0.6654399059325442 0.6642434992016106 0.3551793653867890 T T T

0.7222200000000001 0.4444400000000002 0.1964400000000026 F F F

0.9442192340738345 0.5549768997982696 0.2756229692339005 T T T

0.8323511908708880 0.6645312220199813 0.3551362620463649 T T T

0.7222200000000001 0.6111099999999965 0.1964400000000026 F F F

0.9442108446824236 0.3885105457395426 0.2755594238418055 T T T

0.8324898452647247 0.4985071127232175 0.3548922672109853 T T T

0.8888900000000035 0.6111099999999965 0.1964400000000026 F F F

0.7773362327085067 0.3882949309345349 0.2756957908328843 T T T

0.6654437723583158 0.4982532736305174 0.3551069789059937 T T T

0.2222200000000001 0.7777799999999999 0.1964400000000026 F F F

0.1104866821713557 0.8869214871417483 0.2754445286606355 T T T

0.9985251192743689 0.9965635473363648 0.3549064403573965 T T T

0.0555599999999998 0.7777799999999999 0.1964400000000026 F F F

0.2779415908291654 0.8869769666319272 0.2760821860384794 T T T

0.1655656137563497 0.9937549658114051 0.3544396081031991 T T T

0.0555599999999998 0.9444400000000002 0.1964400000000026 F F F

0.2791096786383633 0.7216721157558368 0.2769105729187658 T T T

0.1666816007916765 0.8302850859753335 0.3551089596476729 T T T

0.2222200000000001 0.9444400000000002 0.1964400000000026 F F F

0.1113953897889530 0.7209149196504185 0.2757788057346673 T T T

0.9991282065317080 0.8301811304017477 0.3553879036366041 T T T

0.5555599999999998 0.7777799999999999 0.1964400000000026 F F F

0.4440559546268119 0.8887702951443401 0.2737479351056251 T T T

0.3329129141549885 0.9973435346995174 0.3533842930583475 T T T

0.3888900000000035 0.7777799999999999 0.1964400000000026 F F F

0.6118414899133526 0.8886769143721835 0.2740074864640931 T T T

0.5009789017498747 0.9983696953289396 0.3533620720609818 T T T

0.3888900000000035 0.9444400000000002 0.1964400000000026 F F F

0.6104430122052662 0.7212505332084405 0.2758316282879676 T T T

0.5013025641199178 0.8329077501623559 0.3496234641520815 T T T

0.5555599999999998 0.9444400000000002 0.1964400000000026 F F F

0.4436754554046018 0.7200245298277963 0.2747226327422112 T T T

0.3357744315094351 0.8327805431864945 0.3565546202323517 T T T

0.8888900000000035 0.7777799999999999 0.1964400000000026 F F F

0.7776115541475406 0.8875934601786710 0.2757432970715925 T T T

0.6658708429991620 0.9977136802863811 0.3547735736635147 T T T

0.7222200000000001 0.7777799999999999 0.1964400000000026 F F F

0.9440519373709293 0.8871625195762576 0.2756414488938788 T T T

0.8328838409869519 0.9979211030074850 0.3553364117558360 T T T

0.7222200000000001 0.9444400000000002 0.1964400000000026 F F F

0.9441121265100065 0.7209486228209130 0.2758585283449762 T T T

0.8324309496011679 0.8307251509270941 0.3549761205268283 T T T

0.8888900000000035 0.9444400000000002 0.1964400000000026 F F F

0.7769251853835968 0.7206414895015101 0.2757538535052078 T T T

0.6663506791882696 0.8314633609629638 0.3541907549412713 T T T

0.2513133848574753 0.5032776701055184 0.5343141576396633 T T T

0.2378892056257911 0.5445208651210223 0.4722179916046372 T T T

0.2503311247395956 0.6195603184607634 0.5271125973969504 T T T

0.4153373755934382 0.4977207689636884 0.5040833339411890 T T T

0.5126895425820592 0.6066495261969465 0.4714204613071971 T T T

0.3918577704976371 0.5303665162933200 0.4420923547112423 T T T

0.4198661480789454 0.7167205396091347 0.5735727863819449 T T T

0.5269480555611494 0.7082557501271509 0.5517331817718656 T T T

0.4220938373658072 0.6004954014574366 0.5812418667659500 T T T

0.2363030133882233 0.7358634206886023 0.4677081486314165 T T T

0.2103738885177506 0.8370803227051908 0.4690637489144152 T T T

0.2520091226126340 0.8631270235642532 0.5629282465336445 T T T

0.2983653322189923 0.7760663567577808 0.5602984672376792 T T T

0.1661329642966223 0.7317606809598303 0.5510391344371612 T T T

0.3413214971299681 0.9968346071137546 0.4412373335185862 T T T

0.4696414270714442 0.0310263475061134 0.4490778730520333 T T T

0.4370450689344452 0.1219064881731242 0.5135200463802718 T T T

0.4382344303577739 0.0211767887393250 0.5479715515989696 T T T

0.3203205932273931 0.0149068697385886 0.5330977512421029 T T T

0.7332453634895231 0.9089563754721937 0.4450239176173056 T T T

0.6285746772556209 0.7967347350981839 0.4691004181383391 T T T

0.7731098464040400 0.8512357920160341 0.5261509524486243 T T T

0.6816263305072567 0.8702868856051609 0.5599468877815355 T T T

0.7918230078180528 0.9758195980048257 0.5325506484324052 T T T

0.6255617019205246 0.0667526687463820 0.4544240891434796 T T T

0.7412896724280742 0.0692230257601523 0.4630301536905534 T T T

0.7131884380227262 0.0531642269172679 0.5585886344992019 T T T

0.5885363888564186 0.0333373080527863 0.5499363181051748 T T T

0.6964117148393001 0.1536189830043710 0.5344942625683657 T T T

0.1139018018351265 0.1599556911719091 0.4696034437852816 T T T

0.1082736049974997 0.2439651876342097 0.4247458523355050 T T T

0.1276214903694353 0.3193908263849394 0.5073200206053059 T T T

0.2427052207103131 0.3936306153881546 0.4727487109553579 T T T

0.2375576108114998 0.3109283700976775 0.5227626060304971 T T T

0.3256856963565156 0.1564081696458146 0.4286566827689953 T T T

0.2519735560712633 0.1485607974998686 0.4822925170502401 T T T

0.4403435491419785 0.2586391431073949 0.4972176376122448 T T T

0.3816965416788136 0.3348512671659947 0.5028959414754937 T T T

0.4489709663981287 0.3403616922365558 0.4462408863004583 T T T

0.4671791838950696 0.8420267089917246 0.4456259740495554 T T T

0.3652883807319753 0.8806456166187004 0.4694732779303337 T T T

0.6113288894293873 0.9247011181209928 0.4714244193537169 T T T

0.4201573486446790 0.7171231385806109 0.4718586907752780 T T T

0.2413972929908041 0.2331615984324955 0.4208129310207696 T T T

0.3921039639244626 0.6290010197584692 0.5031588353359382 T T T

0.2756440042030164 0.5701106564754426 0.5095816469577485 T T T

0.4302988874375018 0.5619375190456282 0.4790149290591756 T T T

0.4435394034461737 0.6660461599696532 0.5558120183001733 T T T

0.2601010056254012 0.8101574200548853 0.4856877045231656 T T T

0.2435683511054165 0.7952596771738527 0.5434104859998499 T T T

0.3940666791282368 0.9879771486591054 0.4678827886485237 T T T

0.3972139528487162 0.0387756510277626 0.5187739406329328 T T T

0.6763239572230333 0.8789392535765543 0.4766740550942643 T T T

0.7335479677281356 0.8955662276576319 0.5269366727987261 T T T

0.6630560827370360 0.0353522733211236 0.4787382954977986 T T T

0.6657678086950156 0.0706921368335500 0.5337007000265999 T T T

0.1609105281188903 0.2357106272765748 0.4516033899760998 T T T

0.1948792672362529 0.3197261405894665 0.4911321553862180 T T T

0.3019117140471863 0.2023121835866206 0.4524593275305335 T T T

0.3982439834901967 0.2889943652458342 0.4763636002383436 T T T

Transition state configuration of precursor decomposing the first ligand on the Cu substrate

1.00000000000000

15.1407000000000007 0.0000000000000000 0.0000000000000000

-7.5703500000000004 13.1122308309999998 0.0000000000000000

0.0000000000000000 0.0000000000000000 26.1812000000000005

Cu H Ta N C

108 39 1 4 16

Selective dynamics

Direct

0.2222200000000001 0.1111099999999965 0.1964400000000026 F F F

0.1109893993143260 0.2237753935472194 0.2751918416707236 T T T

0.9983994462743356 0.3348757598817156 0.3546173109608429 T T T

0.0555599999999998 0.1111099999999965 0.1964400000000026 F F F

0.2776144567832917 0.2261635995985621 0.2770243984368483 T T T

0.1609181452829602 0.3334842585061982 0.3520835631936336 T T T

0.0555599999999998 0.2777799999999999 0.1964400000000026 F F F

0.2771617786932849 0.0564577393281355 0.2752745501730078 T T T

0.1656967876638200 0.1669669866903709 0.3541417714905208 T T T

0.2222200000000001 0.2777799999999999 0.1964400000000026 F F F

0.1118637635603929 0.0564538505267540 0.2752645682803344 T T T

0.9985256695553275 0.1664836370873460 0.3547836363007990 T T T

0.5555599999999998 0.1111099999999965 0.1964400000000026 F F F

0.4425651047877965 0.2223025557601373 0.2755094285301321 T T T

0.3279621312321958 0.3442414635051936 0.3570062997817553 T T T

0.3888900000000035 0.1111099999999965 0.1964400000000026 F F F

0.6093916642298377 0.2226333658965555 0.2756084811864927 T T T

0.4994013524032839 0.3352567406719201 0.3536382549073723 T T T

0.3888900000000035 0.2777799999999999 0.1964400000000026 F F F

0.6111191190753538 0.0565260969945333 0.2751905398813059 T T T

0.4986720299483923 0.1675388346941580 0.3548140593571637 T T T

0.5555599999999998 0.2777799999999999 0.1964400000000026 F F F

0.4438134895304497 0.0555323488235241 0.2754491066200599 T T T

0.3323178604171231 0.1680605403356265 0.3540506337440507 T T T

0.8888900000000035 0.1111099999999965 0.1964400000000026 F F F

0.7765848417079285 0.2227554703845598 0.2756786495701784 T T T

0.6650371572954584 0.3339273053625397 0.3547776166285011 T T T

0.7222200000000001 0.1111099999999965 0.1964400000000026 F F F

0.9435526686096151 0.2228582341428595 0.2756777461096338 T T T

0.8317976192389815 0.3335825011672240 0.3548800049548959 T T T

0.7222200000000001 0.2777799999999999 0.1964400000000026 F F F

0.9456974318770245 0.0566880872675394 0.2754281847448121 T T T

0.8327539215156884 0.1675934949680566 0.3548939998096438 T T T

0.8888900000000035 0.2777799999999999 0.1964400000000026 F F F

0.7784279917590353 0.0564070753813733 0.2753913728703174 T T T

0.6654368589708064 0.1672606270426641 0.3548491817231596 T T T

0.2222200000000001 0.4444400000000002 0.1964400000000026 F F F

0.1115660479880078 0.5554004029432400 0.2754171338903646 T T T

0.0003987174103784 0.6670486278264487 0.3543442875705377 T T T

0.0555599999999998 0.4444400000000002 0.1964400000000026 F F F

0.2789246489798689 0.5562440390588548 0.2772745323571545 T T T

0.1667292841142937 0.6663952094890085 0.3525430392960245 T T T

0.0555599999999998 0.6111099999999965 0.1964400000000026 F F F

0.2782975959504762 0.3928080063617554 0.2761711006980466 T T T

0.1691975308138599 0.5011636760265764 0.3550635998158526 T T T

0.2222200000000001 0.6111099999999965 0.1964400000000026 F F F

0.1112338633548601 0.3906562607103926 0.2753178285945259 T T T

0.9996216801465320 0.5001557474365159 0.3547495307871459 T T T

0.5555599999999998 0.4444400000000002 0.1964400000000026 F F F

0.4439045024448078 0.5564805031035130 0.2776859836332618 T T T

0.3355267104856623 0.6619136861432748 0.3588423133913474 T T T

0.3888900000000035 0.4444400000000002 0.1964400000000026 F F F

0.6111913053363333 0.5561409103025887 0.2753619533115834 T T T

0.5016656719846374 0.6695059285905806 0.3590167814478123 T T T

0.3888900000000035 0.6111099999999965 0.1964400000000026 F F F

0.6094770611409536 0.3893804299948999 0.2753882734081652 T T T

0.5048780917101037 0.5063960429665844 0.3543638109924099 T T T

0.5555599999999998 0.6111099999999965 0.1964400000000026 F F F

0.4416452363556123 0.3898268169159760 0.2772139001652142 T T T

0.3554173046191465 0.5172928513241108 0.3982180709207303 T T T

0.8888900000000035 0.4444400000000002 0.1964400000000026 F F F

0.7774372708652566 0.5553923529328864 0.2755759213433671 T T T

0.6691887120662091 0.6693106646159586 0.3547271796857650 T T T

0.7222200000000001 0.4444400000000002 0.1964400000000026 F F F

0.9445125568599647 0.5554631408481955 0.2756274722247253 T T T

0.8344713153218620 0.6678354952342271 0.3546585534016551 T T T

0.7222200000000001 0.6111099999999965 0.1964400000000026 F F F

0.9434718558865998 0.3890221150880362 0.2753629162784823 T T T

0.8330456890288461 0.5002463613440696 0.3546286045555245 T T T

0.8888900000000035 0.6111099999999965 0.1964400000000026 F F F

0.7765218736841497 0.3887963883512110 0.2755200356462481 T T T

0.6674986311425490 0.5015423572631436 0.3549744995952410 T T T

0.2222200000000001 0.7777799999999999 0.1964400000000026 F F F

0.1113714613205078 0.8890215779054849 0.2753116392027504 T T T

0.0010916889830074 0.0010482025440723 0.3546867117631338 T T T

0.0555599999999998 0.7777799999999999 0.1964400000000026 F F F

0.2778564298027519 0.8890263572097356 0.2750026577486436 T T T

0.1671754610099144 0.0009688595265891 0.3547426969316482 T T T

0.0555599999999998 0.9444400000000002 0.1964400000000026 F F F

0.2799766858549395 0.7217005863488382 0.2762369567004648 T T T

0.1670872068956200 0.8336672395314059 0.3540134179944118 T T T

0.2222200000000001 0.9444400000000002 0.1964400000000026 F F F

0.1117478551061317 0.7221095725093308 0.2750044291812682 T T T

0.0013344838492113 0.8341189049261659 0.3547064171738979 T T T

0.5555599999999998 0.7777799999999999 0.1964400000000026 F F F

0.4455298122759204 0.8895206072402966 0.2749084421748325 T T T

0.3330550804862725 0.0006406690200901 0.3543238022179162 T T T

0.3888900000000035 0.7777799999999999 0.1964400000000026 F F F

0.6120271890433792 0.8896476592353101 0.2748824820806939 T T T

0.5002271046833794 0.0010355637527322 0.3541416951430288 T T T

0.3888900000000035 0.9444400000000002 0.1964400000000026 F F F

0.6105781606358396 0.7223458700262034 0.2763137198567869 T T T

0.5002976941949400 0.8342205785619974 0.3529428704688075 T T T

0.5555599999999998 0.9444400000000002 0.1964400000000026 F F F

0.4448420130225293 0.7216492432636457 0.2762144711169986 T T T

0.3341236607578301 0.8334372999733231 0.3525545249015372 T T T

0.8888900000000035 0.7777799999999999 0.1964400000000026 F F F

0.7796179205748386 0.8901934693959191 0.2748982031342986 T T T

0.6670903503999419 0.0013196827091328 0.3532496994421013 T T T

0.7222200000000001 0.7777799999999999 0.1964400000000026 F F F

0.9453193223004515 0.8891006534600672 0.2753986381297486 T T T

0.8343581588013430 0.0012275054105724 0.3545448219293233 T T T

0.7222200000000001 0.9444400000000002 0.1964400000000026 F F F

0.9445874280997020 0.7219388931184006 0.2753849900325530 T T T

0.8355286902467297 0.8349475671340008 0.3547458290988970 T T T

0.8888900000000035 0.9444400000000002 0.1964400000000026 F F F

0.7780481660932123 0.7225499727107764 0.2753962929852189 T T T

0.6696037333513487 0.8356543067568846 0.3525170735329869 T T T

0.2793779333519170 0.5469770745261843 0.6465846967832752 T T T

0.2225937335671246 0.4847232010342218 0.5879877506083283 T T T

0.2430493626429932 0.6092228524955713 0.6006791330996099 T T T

0.4418578124384139 0.5495759573943997 0.6404582606003287 T T T

0.5090209280018443 0.5763866165303985 0.5812834425166526 T T T

0.3868766306040630 0.4653904831083019 0.5878776648537273 T T T

0.4107752404665467 0.7623089718638398 0.5912621159955934 T T T

0.5236221626895355 0.7537952729185602 0.5879416264280164 T T T

0.4478147986037860 0.7149153663880924 0.6445736167674270 T T T

0.1739348065493238 0.6088070718193249 0.4379048726145521 T T T

0.1703293526425763 0.7219248595012436 0.4486637605835196 T T T

0.1632401270017680 0.6863444537163984 0.5451391876018528 T T T

0.1669176733324524 0.5734722035453551 0.5301445795100509 T T T

0.0622904146617357 0.5868803185627858 0.5093598780195635 T T T

0.3306736952339598 0.8713687242508814 0.4355693987207667 T T T

0.4380927935100867 0.8783589542548181 0.4653572623865020 T T T

0.3717949890803867 0.9625537603115422 0.5195718784681802 T T T

0.3459460932897118 0.8482605481408757 0.5520694870633016 T T T

0.2466126819312838 0.8552096060044576 0.5191042644525022 T T T

0.6652305275135817 0.7354355799888138 0.4444579881268219 T T T

0.5659886956198196 0.6413504270874764 0.4815434184339769 T T T

0.7280161246731554 0.7134002134878914 0.5279207204707673 T T T

0.6489280281239893 0.7495976348954415 0.5616095872817304 T T T

0.7502080178420977 0.8395347727857490 0.5221283665243994 T T T

0.5802101445257766 0.9110395808961538 0.4391755794984213 T T T

0.6925431741367680 0.9088512970511423 0.4520532619983610 T T T

0.6697490793312729 0.9268052629360292 0.5470078826461477 T T T

0.5511399228149391 0.9194118641451283 0.5342963729291444 T T T

0.6661079101794694 0.0244256555976840 0.5105963880913978 T T T

0.1528457369699474 0.2723707139079282 0.4374512445908667 T T T

0.1854844331539293 0.4013117876302220 0.4454481412663747 T T T

0.1239823154307291 0.3102301464503325 0.5248790411763075 T T T

0.2556976768173418 0.4012912817560997 0.5329932624826222 T T T

0.2146839755434538 0.2687754859189040 0.5250700662407303 T T T

0.4326021547706818 0.3545901691980467 0.4256267391066429 T T T

0.3253429376665782 0.2581051831937863 0.4581261561488956 T T T

0.4896470784300600 0.3656122689559507 0.5080862164138317 T T T

0.3817617563393584 0.3603988063801298 0.5376084336235126 T T T

0.4687708993215421 0.4714677029116938 0.5031625413427000 T T T

0.4074223303556382 0.6784005722541581 0.4652013569327718 T T T

0.3100262451853943 0.7320342307088988 0.4644720461221988 T T T

0.5583622553121007 0.7740632129894415 0.4709684313871578 T T T

0.3797555172212475 0.6125410710322136 0.5259358604055417 T T T

0.3118519141094057 0.3832760366794865 0.4317311155407196 T T T

0.3822738403094258 0.6068373746567152 0.5814687200992581 T T T

0.2751191769642973 0.5588143370979791 0.6053692811873351 T T T

0.4332147568641567 0.5458403270981940 0.5986464525024011 T T T

0.4452666470900415 0.7163108424290248 0.6025578235515309 T T T

0.1984536195815808 0.6730545735282756 0.4651656219204073 T T T

0.1452625286762664 0.6279704052632916 0.5157682112417777 T T T

0.3544618196192241 0.8420402803473361 0.4690663564458751 T T T

0.3280097705224614 0.8787794097171475 0.5179695299722898 T T T

0.6190938816370403 0.7244178799592578 0.4795962925752601 T T T

0.6901958575071874 0.7592278603239090 0.5256534296652541 T T T

0.6160819191124012 0.8851965904098386 0.4680624089626705 T T T

0.6264539385407565 0.9418337690124338 0.5180561541291374 T T T

0.2102785052736078 0.3459970994095857 0.4553924521296033 T T T

0.2017803060744005 0.3309612894454682 0.5130928088592264 T T T

0.3730067427590877 0.3420036497788342 0.4543435475755080 T T T

0.4310834580451403 0.3879174718636853 0.5038905798200031 T T T

Final configuration of precursor decomposing the second ligand on the Cu substrate

1.00000000000000

15.1407000000000007 0.0000000000000000 0.0000000000000000

-7.5703500000000004 13.1122308309999998 0.0000000000000000

0.0000000000000000 0.0000000000000000 26.1812000000000005

Cu H Ta N C

108 39 1 4 16

Selective dynamics

Direct

0.2222200000000001 0.1111099999999965 0.1964400000000026 F F F

0.1109255006723248 0.2211645651204044 0.2764467443565993 T T T

0.9972016289045958 0.3334230888206404 0.3545582751734931 T T T

0.0555599999999998 0.1111099999999965 0.1964400000000026 F F F

0.2763745438351319 0.2215853526448043 0.2768876422372387 T T T

0.1618618170876327 0.3361818495362854 0.3526683059438497 T T T

0.0555599999999998 0.2777799999999999 0.1964400000000026 F F F

0.2754903190969588 0.0539716481794506 0.2745840475485793 T T T

0.1643353089282188 0.1595106207671594 0.3571958226064266 T T T

0.2222200000000001 0.2777799999999999 0.1964400000000026 F F F

0.1103521967092459 0.0556310001484288 0.2761527758407841 T T T

0.9965143663966527 0.1649157712467088 0.3532767072912207 T T T

0.5555599999999998 0.1111099999999965 0.1964400000000026 F F F

0.4433530204648215 0.2214151893026504 0.2751049213339460 T T T

0.3258704645530044 0.3288373815583951 0.3638789698965361 T T T

0.3888900000000035 0.1111099999999965 0.1964400000000026 F F F

0.6105003370309487 0.2216188531657848 0.2755688465260869 T T T

0.4978096209509388 0.3305049928965740 0.3543953487513145 T T T

0.3888900000000035 0.2777799999999999 0.1964400000000026 F F F

0.6110218954381013 0.0552698279633755 0.2753550092015495 T T T

0.5005995939567179 0.1653926693535006 0.3548937870132103 T T T

0.5555599999999998 0.2777799999999999 0.1964400000000026 F F F

0.4438996355859104 0.0550143940660473 0.2753302140480581 T T T

0.3346083487145725 0.1616558960395472 0.3517519502976754 T T T

0.8888900000000035 0.1111099999999965 0.1964400000000026 F F F

0.7773934659353879 0.2216950006169508 0.2757391471522251 T T T

0.6644593834848834 0.3313343830101574 0.3551259263823805 T T T

0.7222200000000001 0.1111099999999965 0.1964400000000026 F F F

0.9444001955522306 0.2226895272516442 0.2753117752456543 T T T

0.8316412803133297 0.3327418388590849 0.3550148258661209 T T T

0.7222200000000001 0.2777799999999999 0.1964400000000026 F F F

0.9433512648436588 0.0543623793236831 0.2751960426209538 T T T

0.8319788516996530 0.1660748494614016 0.3551917424488635 T T T

0.8888900000000035 0.2777799999999999 0.1964400000000026 F F F

0.7775963270409552 0.0551289119881619 0.2754707857831495 T T T

0.6666612845988933 0.1662211500230565 0.3552736127048131 T T T

0.2222200000000001 0.4444400000000002 0.1964400000000026 F F F

0.1121903302479709 0.5585208953651667 0.2782059816485893 T T T

0.9908763594391196 0.6616119351781909 0.3517732875583760 T T T

0.0555599999999998 0.4444400000000002 0.1964400000000026 F F F

0.2777994866626576 0.5559117594565525 0.2770535553228330 T T T

0.1683486307724900 0.6698898592418575 0.3647862101247653 T T T

0.0555599999999998 0.6111099999999965 0.1964400000000026 F F F

0.2766844605437457 0.3872999292413947 0.2774521631802593 T T T

0.1658004719266815 0.5027974590988632 0.3557578395565515 T T T

0.2222200000000001 0.6111099999999965 0.1964400000000026 F F F

0.1106416367069272 0.3901843261993853 0.2751717353778670 T T T

0.9977997735886740 0.4996755399520952 0.3546925984731377 T T T

0.5555599999999998 0.4444400000000002 0.1964400000000026 F F F

0.4420795625792143 0.5526660731690746 0.2746086093969068 T T T

0.3371781127243088 0.6680933404039047 0.3589082166760235 T T T

0.3888900000000035 0.4444400000000002 0.1964400000000026 F F F

0.6077904393930912 0.5531009775041993 0.2758024965661824 T T T

0.4975362573454327 0.6589781462038503 0.3523551776622199 T T T

0.3888900000000035 0.6111099999999965 0.1964400000000026 F F F

0.6089258011634572 0.3875506976112297 0.2754961990370516 T T T

0.4952022538120169 0.4949022496708035 0.3559197124392401 T T T

0.5555599999999998 0.6111099999999965 0.1964400000000026 F F F

0.4392851427647544 0.3856473105891777 0.2782887613954613 T T T

0.3309657546784457 0.4991002787210568 0.3564320188130808 T T T

0.8888900000000035 0.4444400000000002 0.1964400000000026 F F F

0.7754785171744170 0.5539474017829497 0.2756501711958919 T T T

0.6619735020800729 0.6619434299493556 0.3547088724576853 T T T

0.7222200000000001 0.4444400000000002 0.1964400000000026 F F F

0.9431998740566773 0.5544250954995229 0.2749799822741295 T T T

0.8279667935377617 0.6633682997821336 0.3542280732076131 T T T

0.7222200000000001 0.6111099999999965 0.1964400000000026 F F F

0.9440444896346197 0.3894284664630469 0.2753452596491876 T T T

0.8289701540181099 0.4966180477880209 0.3550234906196346 T T T

0.8888900000000035 0.6111099999999965 0.1964400000000026 F F F

0.7763532742961178 0.3879810783616160 0.2755623541066380 T T T

0.6631706713352453 0.4968125766098607 0.3550354541694203 T T T

0.2222200000000001 0.7777799999999999 0.1964400000000026 F F F

0.1100685261509355 0.8860253428417377 0.2760052997829172 T T T

0.9963231481540675 0.9972725627526778 0.3545477572635315 T T T

0.0555599999999998 0.7777799999999999 0.1964400000000026 F F F

0.2755283515676986 0.8864474863811068 0.2759222440423156 T T T

0.1633419447089847 0.9949334887457912 0.3533612333012013 T T T

0.0555599999999998 0.9444400000000002 0.1964400000000026 F F F

0.2765932021675758 0.7207810442511664 0.2778426033077810 T T T

0.1604757400201095 0.8307984986510122 0.3569932794660040 T T T

0.2222200000000001 0.9444400000000002 0.1964400000000026 F F F

0.1104231845972940 0.7209185631510143 0.2770285849019685 T T T

0.9942378889248976 0.8309879759861404 0.3534856111364346 T T T

0.5555599999999998 0.7777799999999999 0.1964400000000026 F F F

0.4437106003782610 0.8891845651372856 0.2738809962327520 T T T

0.3327153960529564 0.9985168893570203 0.3524050935677454 T T T

0.3888900000000035 0.7777799999999999 0.1964400000000026 F F F

0.6104625974233742 0.8877335094062585 0.2755208318212166 T T T

0.5009704503578404 0.0009568518338308 0.3543363598654407 T T T

0.3888900000000035 0.9444400000000002 0.1964400000000026 F F F

0.6095307406099109 0.7209468805803856 0.2752438752726857 T T T

0.4991215271147635 0.8361100591374957 0.3526695253154532 T T T

0.5555599999999998 0.9444400000000002 0.1964400000000026 F F F

0.4434509967430608 0.7212647157846164 0.2749100587086122 T T T

0.3379868967819473 0.8350770088479750 0.3529378012940237 T T T

0.8888900000000035 0.7777799999999999 0.1964400000000026 F F F

0.7756720532916085 0.8872337483007677 0.2761046649989743 T T T

0.6663276706944283 0.0000154465720306 0.3539613513071800 T T T

0.7222200000000001 0.7777799999999999 0.1964400000000026 F F F

0.9432143915195889 0.8879533411792743 0.2746964424681781 T T T

0.8319320223918575 0.9991876936391512 0.3544109147258948 T T T

0.7222200000000001 0.9444400000000002 0.1964400000000026 F F F

0.9426655512768828 0.7215056517687808 0.2747327397255401 T T T

0.8308050798697594 0.8310121489856005 0.3533698828884279 T T T

0.8888900000000035 0.9444400000000002 0.1964400000000026 F F F

0.7748279842704594 0.7202440950924410 0.2754935092342427 T T T

0.6650558285310355 0.8315911672289295 0.3563096329958985 T T T

0.2279342704313911 0.5014409263152414 0.5566171184873682 T T T

0.2379610533395382 0.5239488261928397 0.4891901032879534 T T T

0.2350560602332775 0.6138816160950028 0.5315565316884752 T T T

0.3787997093671074 0.4874030521656165 0.5699919704333328 T T T

0.5003201662479335 0.5802504881091411 0.5456439457468532 T T T

0.4047604297545091 0.4942661980370137 0.5029507044779600 T T T

0.3943789716979551 0.7480996001337150 0.5721607802034635 T T T

0.4958619792738777 0.7227487223132097 0.5840788367140565 T T T

0.3738838620664109 0.6444513992244882 0.6124464319638473 T T T

0.0019914012476719 0.6976003126696759 0.4293290213198854 T T T

0.0730304229168804 0.7662720070667178 0.4828885841353605 T T T

0.1002930353339565 0.6167601222994809 0.5055059561483387 T T T

0.0327192374561676 0.5539359791090096 0.4491282029884423 T T T

0.9687299699958573 0.5791858213819566 0.4992068608618863 T T T

0.2316326999898008 0.8977818936398875 0.4640724301062949 T T T

0.3159903626042420 0.8766758639099850 0.4265577811690069 T T T

0.3495887230811409 0.8668450432129532 0.5159202243046060 T T T

0.3042663951126670 0.7452125118013662 0.4873235822560293 T T T

0.2229855498122262 0.7683897805019639 0.5282901371023726 T T T

0.8111795467676799 0.8695431055299325 0.4742244809880335 T T T

0.7488976001859156 0.7556697381248105 0.4382709007358129 T T T

0.7797279446084971 0.7162459071003181 0.5247726308529053 T T T

0.6463081836280421 0.6582065192235673 0.5137445583418275 T T T

0.7091965590749325 0.7689464113505355 0.5529130769426265 T T T

0.5915181461467122 0.9109579670107369 0.4625125461647593 T T T

0.7224803345944389 0.9789904034661719 0.4515677213080453 T T T

0.7550757767258567 0.9445820339275731 0.5431547893177699 T T T

0.6206481505410683 0.8727891349395084 0.5537786799934785 T T T

0.6814156533110945 0.0059340678726085 0.5399089015665164 T T T

0.1093966395267210 0.1625141246680646 0.4695764016690023 T T T

0.1132273765638159 0.2540386023631870 0.4265666853643160 T T T

0.1359859976782810 0.3250434547878118 0.5105048232724776 T T T

0.2544306576047049 0.3935570887452655 0.4777073521206448 T T T

0.2377221590215364 0.3030653077018357 0.5258510173832652 T T T

0.3140179283700052 0.1449579903203208 0.4290330694890937 T T T

0.2419910322231001 0.1406960603150954 0.4826472884046713 T T T

0.4296591086393722 0.2372632726639765 0.4988786988976872 T T T

0.3813986764543582 0.3228179992887928 0.5052226741392881 T T T

0.4474057669105052 0.3243374225110126 0.4487145943003927 T T T

0.5113638841125761 0.7491145524952956 0.4348435778086719 T T T

0.1619408385395113 0.7600142821454733 0.4223283705926730 T T T

0.6588350530144232 0.8190156722886892 0.4555689624152585 T T T

0.4263861865092091 0.6748461545665884 0.4857145358545334 T T T

0.2393016725870626 0.2314946440805546 0.4220015866253335 T T T

0.3801594951939937 0.6159391039320889 0.5312005718520257 T T T

0.2629981273926402 0.5601442564939862 0.5267639891466896 T T T

0.4180319477465761 0.5392927117784214 0.5378620205595871 T T T

0.4132228102980556 0.6874755938575798 0.5778367476214721 T T T

0.0682235453712479 0.7111622298166489 0.4536590777950144 T T T

0.0416692562436090 0.6099194533809902 0.4786142960371076 T T T

0.2488628551025426 0.8375919386482447 0.4524735375502159 T T T

0.2823590253653651 0.8013094658858329 0.4987266078207390 T T T

0.7384931790564319 0.7968539690330874 0.4706333803200158 T T T

0.7168441101672749 0.7317181453438548 0.5185379211106907 T T T

0.6652039780998747 0.9131695980542397 0.4741796120166029 T T T

0.6810376251799135 0.9346836735527315 0.5312396154565941 T T T

0.1615120324944370 0.2385181047703504 0.4529740758819510 T T T

0.2001367367401927 0.3194443245401050 0.4943466177007432 T T T

0.2945052197801152 0.1936255779242701 0.4532816945923280 T T T

0.3934196402981653 0.2743050641377895 0.4782486951512450 T T T

Transition state configuration of precursor decomposing the second ligand on the Cu substrate

1.00000000000000

15.1407000000000007 0.0000000000000000 0.0000000000000000

-7.5703500000000004 13.1122308309999998 0.0000000000000000

0.0000000000000000 0.0000000000000000 26.1812000000000005

Cu H Ta N C

108 39 1 4 16

Selective dynamics

Direct

0.2222200000000001 0.1111099999999965 0.1964400000000026 F F F

0.1111551402753015 0.2207093479679387 0.2761439671548513 T T T

0.9973588430955177 0.3323169791713146 0.3546196123387330 T T T

0.0555599999999998 0.1111099999999965 0.1964400000000026 F F F

0.2771110361597874 0.2218874829716069 0.2766941808672581 T T T

0.1622123069562768 0.3347262998665111 0.3523394089179395 T T T

0.0555599999999998 0.2777799999999999 0.1964400000000026 F F F

0.2764673799636700 0.0540519549247157 0.2745363241036011 T T T

0.1655819087065472 0.1597346366061594 0.3570226061974279 T T T

0.2222200000000001 0.2777799999999999 0.1964400000000026 F F F

0.1109106312884244 0.0554109113189715 0.2762152041000256 T T T

0.9968780700349850 0.1643201173427357 0.3532149946559707 T T T

0.5555599999999998 0.1111099999999965 0.1964400000000026 F F F

0.4448158725622134 0.2226655389257383 0.2750462907112000 T T T

0.3272491198611944 0.3299516713121179 0.3637029276802984 T T T

0.3888900000000035 0.1111099999999965 0.1964400000000026 F F F

0.6114441217716901 0.2226996098516226 0.2754569494014935 T T T

0.4996508347551989 0.3324623932266045 0.3544564270008808 T T T

0.3888900000000035 0.2777799999999999 0.1964400000000026 F F F

0.6105523046903411 0.0552224596938277 0.2752837175320581 T T T

0.5022875281333729 0.1669135967976890 0.3548283983704157 T T T

0.5555599999999998 0.2777799999999999 0.1964400000000026 F F F

0.4449676112150195 0.0560753335791845 0.2753197833481405 T T T

0.3363750920443905 0.1632980048281678 0.3515311313378142 T T T

0.8888900000000035 0.1111099999999965 0.1964400000000026 F F F

0.7772503933673524 0.2216499430413969 0.2756477074629336 T T T

0.6662250120286020 0.3330133017548580 0.3551934473440738 T T T

0.7222200000000001 0.1111099999999965 0.1964400000000026 F F F

0.9444218989832027 0.2224575019933659 0.2752170038046314 T T T

0.8323656959456529 0.3328497148445350 0.3549484733091777 T T T

0.7222200000000001 0.2777799999999999 0.1964400000000026 F F F

0.9438885369374903 0.0540257632118410 0.2751575951810736 T T T

0.8319912202301735 0.1655420581220999 0.3549180311840786 T T T

0.8888900000000035 0.2777799999999999 0.1964400000000026 F F F

0.7782218664809758 0.0555659403824602 0.2753408197886599 T T T

0.6668329104884695 0.1665153539125953 0.3549610335756172 T T T

0.2222200000000001 0.4444400000000002 0.1964400000000026 F F F

0.1118548785571162 0.5566217393107171 0.2765912320128167 T T T

0.9967499359769976 0.6638248166970544 0.3535782286450542 T T T

0.0555599999999998 0.4444400000000002 0.1964400000000026 F F F

0.2778115584051619 0.5554524466549843 0.2769751403400916 T T T

0.1671368862812284 0.6640811244109266 0.3568170149893561 T T T

0.0555599999999998 0.6111099999999965 0.1964400000000026 F F F

0.2777595527913435 0.3875509004883719 0.2769079214258885 T T T

0.1658898039030334 0.4998278560222358 0.3557276132793515 T T T

0.2222200000000001 0.6111099999999965 0.1964400000000026 F F F

0.1108513530542085 0.3893492863384742 0.2750219449671798 T T T

0.9989156468450524 0.4993168133116258 0.3550749543520296 T T T

0.5555599999999998 0.4444400000000002 0.1964400000000026 F F F

0.4439010437153663 0.5547829579424453 0.2766400053723971 T T T

0.3364582843654289 0.6654665508063530 0.3589238622089440 T T T

0.3888900000000035 0.4444400000000002 0.1964400000000026 F F F

0.6095156306084647 0.5544862834999734 0.2760041008843395 T T T

0.5007822312313754 0.6661553761324521 0.3574680067251007 T T T

0.3888900000000035 0.6111099999999965 0.1964400000000026 F F F

0.6104568502409652 0.3890592218169869 0.2756389645880449 T T T

0.4972064699614058 0.4974752570418027 0.3556711334787021 T T T

0.5555599999999998 0.6111099999999965 0.1964400000000026 F F F

0.4404118836968778 0.3865070570928738 0.2780425018716859 T T T

0.3322956731035659 0.4985710944049863 0.3556197017392074 T T T

0.8888900000000035 0.4444400000000002 0.1964400000000026 F F F

0.7769285953976458 0.5550450649010984 0.2758716303681655 T T T

0.6644288633055531 0.6646893629978343 0.3554119211910436 T T T

0.7222200000000001 0.4444400000000002 0.1964400000000026 F F F

0.9440774033181626 0.5550064302816041 0.2753241030800053 T T T

0.8312113038034206 0.6649688931243035 0.3550147409662547 T T T

0.7222200000000001 0.6111099999999965 0.1964400000000026 F F F

0.9442423016933645 0.3890908148494743 0.2754387394679233 T T T

0.8319029224626853 0.4987673114696390 0.3550935355491846 T T T

0.8888900000000035 0.6111099999999965 0.1964400000000026 F F F

0.7772628732009419 0.3888776672304237 0.2756562740110655 T T T

0.6653509463464869 0.4989264256283856 0.3551704953029145 T T T

0.2222200000000001 0.7777799999999999 0.1964400000000026 F F F

0.1111271329296627 0.8861709951991218 0.2763600321989420 T T T

0.9974078496508342 0.9973602332312828 0.3545174724885732 T T T

0.0555599999999998 0.7777799999999999 0.1964400000000026 F F F

0.2767190947481177 0.8867388725309513 0.2767308726256374 T T T

0.1648674781712805 0.9961083385802384 0.3533825052550382 T T T

0.0555599999999998 0.9444400000000002 0.1964400000000026 F F F

0.2786354842118564 0.7219456921309697 0.2775576045253172 T T T

0.1683038850159873 0.8346422471562770 0.3582186447303345 T T T

0.2222200000000001 0.9444400000000002 0.1964400000000026 F F F

0.1115873250816867 0.7221917168768613 0.2772098677714987 T T T

0.9971704450085769 0.8310199661934732 0.3542643065847129 T T T

0.5555599999999998 0.7777799999999999 0.1964400000000026 F F F

0.4410286462205135 0.8895223008751726 0.2734488135683810 T T T

0.3358877071193263 0.0014108413224687 0.3521992243435463 T T T

0.3888900000000035 0.7777799999999999 0.1964400000000026 F F F

0.6109642741335017 0.8884793411033697 0.2737166517276214 T T T

0.5002823729738838 0.9998594781726102 0.3547190639221220 T T T

0.3888900000000035 0.9444400000000002 0.1964400000000026 F F F

0.6096322232822057 0.7212319608378084 0.2758484790978589 T T T

0.4962400259788125 0.8368307623886113 0.3468816225366141 T T T

0.5555599999999998 0.9444400000000002 0.1964400000000026 F F F

0.4433588213731746 0.7199667678785915 0.2744587424944046 T T T

0.3335787573291759 0.8291956274332459 0.3597182950941691 T T T

0.8888900000000035 0.7777799999999999 0.1964400000000026 F F F

0.7760908770083239 0.8874532873940192 0.2761197117744522 T T T

0.6659453432864717 -0.0003173907081071 0.3528152765866841 T T T

0.7222200000000001 0.7777799999999999 0.1964400000000026 F F F

0.9436466752559244 0.8876439435368931 0.2751170120983271 T T T

0.8322267354891333 0.9991104712917751 0.3542177742948731 T T T

0.7222200000000001 0.9444400000000002 0.1964400000000026 F F F

0.9433672174030301 0.7216447825722141 0.2754457931041431 T T T

0.8318022807756437 0.8317942465269842 0.3543143205094478 T T T

0.8888900000000035 0.9444400000000002 0.1964400000000026 F F F

0.7755114015661206 0.7208498781255233 0.2759442594281025 T T T

0.6644063611131883 0.8324618518270617 0.3555893839929571 T T T

0.2395759433729446 0.5027008704869507 0.5482053533700666 T T T

0.2414064838439156 0.5324625520793023 0.4818864420152098 T T T

0.2447950970503182 0.6181329609167759 0.5291766255277984 T T T

0.3986184841838400 0.4875698335282230 0.5444004092891950 T T T

0.5124074486648765 0.5874573561857042 0.5161952560690631 T T T

0.4071625744800569 0.5081708209381131 0.4767164834044805 T T T

0.4016419138142603 0.7364422624558604 0.5739377161442116 T T T

0.5109012377112446 0.7209301015092929 0.5719443793599818 T T T

0.3974984902926572 0.6282373233587851 0.6035281829518366 T T T

0.0491716423432210 0.7152893843197995 0.4325464755774701 T T T

0.1001229955621592 0.8085119703507502 0.4810757502741783 T T T

0.1707844641650935 0.7064117182436496 0.5243325054365802 T T T

0.1279465618865026 0.6202835644314698 0.4717619686042003 T T T

0.0381992223028259 0.6308712968619113 0.5116128116625367 T T T

0.2646896361055039 0.9615555977870138 0.4604109817778483 T T T

0.3553208512012112 0.9400449066329506 0.4285810589153406 T T T

0.3925928774469076 0.9572547736370078 0.5176418555430766 T T T

0.3475958065004379 0.8264710485553680 0.5025583796857204 T T T

0.2678071266341964 0.8633928777767574 0.5358188695459468 T T T

0.8104501572608021 0.9004255025480077 0.4679048594161211 T T T

0.7286244239475623 0.7734627072118573 0.4481202758828479 T T T

0.7857941303059457 0.7665903786854763 0.5335325655241140 T T T

0.6537959646907465 0.7269829374981941 0.5370445680889095 T T T

0.7462546412945776 0.8504044838967966 0.5589270277452499 T T T

0.5976923810055768 0.9544587833213252 0.4569699449452427 T T T

0.7304826233417082 0.0151534664377633 0.4513537416825362 T T T

0.7443838767860437 0.9928212968787162 0.5470612495245984 T T T

0.6086634300542435 0.9251584079948024 0.5517982120281919 T T T

0.6755232466743594 0.0564750421022072 0.5334450792778193 T T T

0.1102831858934214 0.1605827732266704 0.4690379520390468 T T T

0.1115892719606053 0.2508497163491947 0.4259936195334343 T T T

0.1315770486939707 0.3206910326535154 0.5099237772569588 T T T

0.2496657966195142 0.3926159727580518 0.4773763705298039 T T T

0.2361058973372503 0.3030629205540932 0.5253834955200392 T T T

0.3173064904917694 0.1479268668015353 0.4289508875488784 T T T

0.2450761466348803 0.1433629175384670 0.4825824937621499 T T T

0.4328314935381298 0.2432755911661955 0.4983834325528808 T T T

0.3824312245188355 0.3272333734083003 0.5039055400359931 T T T

0.4485296688660552 0.3284049527194472 0.4476231276358148 T T T

0.5148018727424525 0.7841751272802747 0.4340148844407620 T T T

0.2065840023147523 0.8155500575200588 0.4270854398702492 T T T

0.6560073547153930 0.8550743053004233 0.4615475539392485 T T T

0.4268075054946256 0.6911934198385375 0.4783126163813349 T T T

0.2401825567807274 0.2321568877811283 0.4216393302205278 T T T

0.3882206451488497 0.6204381092802993 0.5202029526151172 T T T

0.2711661625114860 0.5645663564703439 0.5198432785797636 T T T

0.4286776579495495 0.5458717574059561 0.5141814610166654 T T T

0.4271509580532572 0.6802208353177451 0.5705948610724203 T T T

0.1154724454961798 0.7553735649117364 0.4586957176567118 T T T

0.1139702880183667 0.6740362105706833 0.4936937386149934 T T T

0.2879303369539194 0.9033955833666077 0.4545331476932148 T T T

0.3253552727088312 0.8852754913396159 0.5055488792457983 T T T

0.7353050606791451 0.8321631920302476 0.4753985052080198 T T T

0.7299471355412114 0.7924600127008091 0.5295743070237539 T T T

0.6668660797830390 0.9540355509459559 0.4736694731606987 T T T

0.6739015132338625 0.9832490435055747 0.5299276204972211 T T T

0.1609733146244233 0.2370404697600529 0.4525003276535613 T T T

0.1973098130231804 0.3178586590423264 0.4938755649516287 T T T

0.2969120481485030 0.1962112543977296 0.4529862443073994 T T T

0.3953199360274845 0.2786114576288305 0.4774509781308534 T T T

Configuration of SiO2 substrate

1.00000000000000

14.7629999999999999 0.0000000000000000 0.0000000000000000

-7.3815000000000000 12.7851330359999995 0.0000000000000000

0.0000000000000000 0.0000000000000000 30.1555999999999997

O Si H

72 36 18

Selective dynamics

Direct

0.0677568828833657 0.1232787953372565 0.2147573248365049 T T T

0.0883299999999991 0.1360000000000028 0.0383199999999988 F F F

0.0476699999999965 0.2449999999999974 0.0980100000000022 F F F

0.1882699099088754 0.2834064923437865 0.1609559931661835 T T T

0.1360000000000028 0.0883299999999991 0.1407499999999970 F F F

0.2449999999999974 0.0476699999999965 0.0810600000000008 F F F

0.2801503667950769 0.2097245916339716 0.2065611835103525 T T T

0.2856700000000032 0.1973300000000009 0.0213699999999974 F F F

0.4012837630442974 0.1224513617976086 0.2149880631062615 T T T

0.4216699999999989 0.1360000000000028 0.0383199999999988 F F F

0.3810000000000002 0.2449999999999974 0.0980100000000022 F F F

0.5209256083723162 0.2831205830810006 0.1610390179743888 T T T

0.4693299999999994 0.0883299999999991 0.1407499999999970 F F F

0.5783300000000011 0.0476699999999965 0.0810600000000008 F F F

0.6136541257695356 0.2101512629440521 0.2063614640854396 T T T

0.6189999999999998 0.1973300000000009 0.0213699999999974 F F F

0.7349924148349913 0.1227997515932202 0.2150387020674884 T T T

0.7550000000000026 0.1360000000000028 0.0383199999999988 F F F

0.7143299999999968 0.2449999999999974 0.0980100000000022 F F F

0.8542735177582159 0.2832356386930428 0.1610190987997626 T T T

0.8026699999999991 0.0883299999999991 0.1407499999999970 F F F

0.9116700000000009 0.0476699999999965 0.0810600000000008 F F F

0.9475176711988738 0.2111706904513611 0.2061625596314300 T T T

0.9523300000000035 0.1973300000000009 0.0213699999999974 F F F

0.0672940350960829 0.4558301640706759 0.2145170846889002 T T T

0.0883299999999991 0.4693299999999994 0.0383199999999988 F F F

0.0476699999999965 0.5783300000000011 0.0980100000000022 F F F

0.1882526769791895 0.6169896417603269 0.1610746692673786 T T T

0.1360000000000028 0.4216699999999989 0.1407499999999970 F F F

0.2449999999999974 0.3810000000000002 0.0810600000000008 F F F

0.2800553094036360 0.5427290727808725 0.2063059174174455 T T T

0.2856700000000032 0.5306700000000006 0.0213699999999974 F F F

0.4025610774196053 0.4578986404219094 0.2151710852201631 T T T

0.4216699999999989 0.4693299999999994 0.0383199999999988 F F F

0.3810000000000002 0.5783300000000011 0.0980100000000022 F F F

0.5216496465588776 0.6167698850918413 0.1607979500648966 T T T

0.4693299999999994 0.4216699999999989 0.1407499999999970 F F F

0.5783300000000011 0.3810000000000002 0.0810600000000008 F F F

0.6145103435498385 0.5436909938195313 0.2059529129703179 T T T

0.6189999999999998 0.5306700000000006 0.0213699999999974 F F F

0.7348824261879301 0.4567107649417039 0.2149020467412228 T T T

0.7550000000000026 0.4693299999999994 0.0383199999999988 F F F

0.7143299999999968 0.5783300000000011 0.0980100000000022 F F F

0.8548811199961648 0.6167174881312860 0.1609582952129642 T T T

0.8026699999999991 0.4216699999999989 0.1407499999999970 F F F

0.9116700000000009 0.3810000000000002 0.0810600000000008 F F F

0.9470041876066091 0.5429916109354735 0.2063659933581405 T T T

0.9523300000000035 0.5306700000000006 0.0213699999999974 F F F

0.0679494212471567 0.7895518584698422 0.2149331046409344 T T T

0.0883299999999991 0.8026699999999991 0.0383199999999988 F F F

0.0476699999999965 0.9116700000000009 0.0980100000000022 F F F

0.1877535729499868 0.9499189626096864 0.1609989388265163 T T T

0.1360000000000028 0.7550000000000026 0.1407499999999970 F F F

0.2449999999999974 0.7143299999999968 0.0810600000000008 F F F

0.2804399365061983 0.8773138443378699 0.2063555393458216 T T T

0.2856700000000032 0.8639999999999972 0.0213699999999974 F F F

0.4011206497729916 0.7899300874813306 0.2148485640019970 T T T

0.4216699999999989 0.8026699999999991 0.0383199999999988 F F F

0.3810000000000002 0.9116700000000009 0.0980100000000022 F F F

0.5213258723915573 0.9499355591201422 0.1609224496861543 T T T

0.4693299999999994 0.7550000000000026 0.1407499999999970 F F F

0.5783300000000011 0.7143299999999968 0.0810600000000008 F F F

0.6133058160617040 0.8764138867341273 0.2064819441309993 T T T

0.6189999999999998 0.8639999999999972 0.0213699999999974 F F F

0.7339082122340557 0.7888211810616639 0.2147523185200129 T T T

0.7550000000000026 0.8026699999999991 0.0383199999999988 F F F

0.7143299999999968 0.9116700000000009 0.0980100000000022 F F F

0.8543326634063249 0.9498278729803786 0.1610280804630762 T T T

0.8026699999999991 0.7550000000000026 0.1407499999999970 F F F

0.9116700000000009 0.7143299999999968 0.0810600000000008 F F F

0.9464757584481163 0.8766894279988762 0.2065468031075284 T T T

0.9523300000000035 0.8639999999999972 0.0213699999999974 F F F

0.9999481585385368 0.1566137983661520 0.0598481585633692 T T T

0.1561074898706067 0.9994250702285541 0.1198520230459721 T T T

0.1703898969462472 0.1732173962078889 0.1810055034884002 T T T

0.1842463637772553 0.2030117378908258 0.0008944479472177 T T T

0.3332766958757816 0.1566357436875876 0.0598235083521388 T T T

0.4894223947978915 0.9994235821565027 0.1198622786111869 T T T

0.5035049287569393 0.1730405136391013 0.1810502493133157 T T T

0.5178137093418727 0.2036706712072061 0.0010554970995145 T T T

0.6666209997587060 0.1566185292791431 0.0598403877774416 T T T

0.8227064117464309 0.9993924700138308 0.1198868618392197 T T T

0.8370196605690978 0.1732725223458331 0.1809991914998150 T T T

0.8507915458562962 0.2027777663406098 0.0008350564869275 T T T

0.9999552881618570 0.4899432558901253 0.0598256571345814 T T T

0.1560905760109961 0.3327276853594014 0.1198897020906600 T T T

0.1701828196777626 0.5065694630710667 0.1809329486655216 T T T

0.1840619216466237 0.5358412609354630 0.0007638798164464 T T T

0.3332665418896568 0.4899568623690769 0.0598519625510505 T T T

0.4894071557016346 0.3326893610029913 0.1198889324280472 T T T

0.5042391425423745 0.5068738999521969 0.1809016869058553 T T T

0.5176740557573041 0.5366476307556240 0.0009613355281033 T T T

0.6666145646320132 0.4899708716717939 0.0598362968367852 T T T

0.8227259785383865 0.3327543021193335 0.1198595469519148 T T T

0.8371961824558554 0.5065664435813915 0.1809769517881108 T T T

0.8510853311824746 0.5369484302377359 0.0010329259806596 T T T

0.9999330053291757 0.8233136381152306 0.0598486625783465 T T T

0.1561275655554866 0.6660948017515906 0.1198710942844770 T T T

0.1702133900896712 0.8398847321144700 0.1810100679506021 T T T

0.1844009074914297 0.8701204769616950 0.0009894002764597 T T T

0.3332844131569104 0.8232804111648164 0.0598352891282303 T T T

0.4893863412242752 0.6661483467616662 0.1198436791888398 T T T

0.5035278878352472 0.8398462458409668 0.1810079593294631 T T T

0.5175333017907278 0.8695983008944665 0.0008556717211334 T T T

0.6665929515092373 0.8232653420053424 0.0598483600798900 T T T

0.8227736464830286 0.6660524235970868 0.1198808125027497 T T T

0.8365828410107881 0.8396556708428378 0.1810375510967717 T T T

0.8508626018565835 0.8696618286258797 0.0008747852762028 T T T

0.0285046448332622 0.0463094994677746 0.2153916478706464 T T T

0.3155260641075301 0.1681818504167012 0.2114221728862091 T T T

0.3626812611064665 0.0454432353523657 0.2155104466949780 T T T

0.6490373921214854 0.1686909717673544 0.2114117873813228 T T T

0.6962936133816768 0.0458325011935727 0.2157657126061636 T T T

0.9833079676678480 0.1700938400350935 0.2113206003494099 T T T

0.0280969477176356 0.3787517075459803 0.2146338291897507 T T T

0.3157367517634526 0.5013377132140491 0.2108870143366985 T T T

0.3646670143617996 0.3810765046698137 0.2170159912219702 T T T

0.6506755758913911 0.5027876304180836 0.2108088757421385 T T T

0.6961235717458720 0.3797857748046241 0.2158485944207200 T T T

0.9829104528065571 0.5017327746818907 0.2110013763292429 T T T

0.0291294340095689 0.7125798631799043 0.2155267861899048 T T T

0.3161234426093174 0.8361167647577048 0.2113713291226276 T T T

0.3624365490368433 0.7129973976042763 0.2157613468991002 T T T

0.6489144320592644 0.8350245668234990 0.2113583010618214 T T T

0.6951846773780588 0.7118040147653133 0.2149991594577187 T T T

0.9821928727139806 0.8355044877202715 0.2114727079763981 T T T

Final configuration of precursor adsorbing on SiO2 substrate

O Si H Ta N C

1.00000000000000

14.7629999999999999 0.0000000000000000 0.0000000000000000

-7.3815000000000000 12.7851330359999995 0.0000000000000000

0.0000000000000000 0.0000000000000000 30.1555999999999997

O Si H Ta N C

72 36 57 1 4 16

Selective dynamics

Direct

0.0628391781948068 0.1234097098445304 0.2137109804696564 T T T

0.0883299999999991 0.1360000000000028 0.0383199999999988 F F F

0.0476699999999965 0.2449999999999974 0.0980100000000022 F F F

0.1885912103501042 0.2822668918047384 0.1606514191212014 T T T

0.1360000000000028 0.0883299999999991 0.1407499999999970 F F F

0.2449999999999974 0.0476699999999965 0.0810600000000008 F F F

0.2693571643060224 0.1950579893618780 0.2098061065107686 T T T

0.2856700000000032 0.1973300000000009 0.0213699999999974 F F F

0.4007579082020030 0.1205501872703749 0.2147751407088165 T T T

0.4216699999999989 0.1360000000000028 0.0383199999999988 F F F

0.3810000000000002 0.2449999999999974 0.0980100000000022 F F F

0.5209084008880183 0.2827779861851667 0.1610176578304987 T T T

0.4693299999999994 0.0883299999999991 0.1407499999999970 F F F

0.5783300000000011 0.0476699999999965 0.0810600000000008 F F F

0.6129540372209590 0.2105095317546883 0.2069664677339418 T T T

0.6189999999999998 0.1973300000000009 0.0213699999999974 F F F

0.7298776980350539 0.1210586591341141 0.2139038800080044 T T T

0.7550000000000026 0.1360000000000028 0.0383199999999988 F F F

0.7143299999999968 0.2449999999999974 0.0980100000000022 F F F

0.8536163128006393 0.2828835027242391 0.1611652988411407 T T T

0.8026699999999991 0.0883299999999991 0.1407499999999970 F F F

0.9116700000000009 0.0476699999999965 0.0810600000000008 F F F

0.9414087658475339 0.2066333027126532 0.2085183293930655 T T T

0.9523300000000035 0.1973300000000009 0.0213699999999974 F F F

0.0720392857039940 0.4608617515019802 0.2147157471505778 T T T

0.0883299999999991 0.4693299999999994 0.0383199999999988 F F F

0.0476699999999965 0.5783300000000011 0.0980100000000022 F F F

0.1893702079211508 0.6172754624149945 0.1603290726473290 T T T

0.1360000000000028 0.4216699999999989 0.1407499999999970 F F F

0.2449999999999974 0.3810000000000002 0.0810600000000008 F F F

0.2849091716988710 0.5409317836231295 0.2059068148424004 T T T

0.2856700000000032 0.5306700000000006 0.0213699999999974 F F F

0.4054876698316150 0.4736642637422861 0.2167979346327016 T T T

0.4216699999999989 0.4693299999999994 0.0383199999999988 F F F

0.3810000000000002 0.5783300000000011 0.0980100000000022 F F F

0.5287613089467271 0.6192412732501182 0.1588832581197224 T T T

0.4693299999999994 0.4216699999999989 0.1407499999999970 F F F

0.5783300000000011 0.3810000000000002 0.0810600000000008 F F F

0.6089671900220068 0.5363154445077853 0.2072518329507531 T T T

0.6189999999999998 0.5306700000000006 0.0213699999999974 F F F

0.7367455300932804 0.4551035733174515 0.2153822115951485 T T T

0.7550000000000026 0.4693299999999994 0.0383199999999988 F F F

0.7143299999999968 0.5783300000000011 0.0980100000000022 F F F

0.8542892591765181 0.6164468933145173 0.1611138879929399 T T T

0.8026699999999991 0.4216699999999989 0.1407499999999970 F F F

0.9116700000000009 0.3810000000000002 0.0810600000000008 F F F

0.9487395041451379 0.5446492151990299 0.2062942607630731 T T T

0.9523300000000035 0.5306700000000006 0.0213699999999974 F F F

0.0642327209633038 0.7882815163082668 0.2140618603570061 T T T

0.0883299999999991 0.8026699999999991 0.0383199999999988 F F F

0.0476699999999965 0.9116700000000009 0.0980100000000022 F F F

0.1877361960820636 0.9498338416561154 0.1610712001332431 T T T

0.1360000000000028 0.7550000000000026 0.1407499999999970 F F F

0.2449999999999974 0.7143299999999968 0.0810600000000008 F F F

0.2766022889943685 0.8740582023467738 0.2077138722066678 T T T

0.2856700000000032 0.8639999999999972 0.0213699999999974 F F F

0.3940536104921151 0.7820917396516052 0.2140741449100219 T T T

0.4216699999999989 0.8026699999999991 0.0383199999999988 F F F

0.3810000000000002 0.9116700000000009 0.0980100000000022 F F F

0.5183329202815088 0.9484464290156112 0.1615298065234043 T T T

0.4693299999999994 0.7550000000000026 0.1407499999999970 F F F

0.5783300000000011 0.7143299999999968 0.0810600000000008 F F F

0.6066411492975661 0.8748241647622663 0.2091345567910921 T T T

0.6189999999999998 0.8639999999999972 0.0213699999999974 F F F

0.7291768726403021 0.7889835408828390 0.2128843345717399 T T T

0.7550000000000026 0.8026699999999991 0.0383199999999988 F F F

0.7143299999999968 0.9116700000000009 0.0980100000000022 F F F

0.8556153950957253 0.9506233507503623 0.1609181766493677 T T T

0.8026699999999991 0.7550000000000026 0.1407499999999970 F F F

0.9116700000000009 0.7143299999999968 0.0810600000000008 F F F

0.9412665808534797 0.8735111148699843 0.2087192992088021 T T T

0.9523300000000035 0.8639999999999972 0.0213699999999974 F F F

0.9999499999999983 0.1566100000000006 0.0598499999999973 F F F

0.1561099999999982 0.9994299999999967 0.1198499999999996 F F F

0.1681529038176279 0.1720025018200957 0.1809992276404984 T T T

0.1842499999999987 0.2030099999999990 0.0008899999999983 F F F

0.3332800000000020 0.1566400000000030 0.0598200000000020 F F F

0.4894200000000026 0.9994200000000006 0.1198600000000027 F F F

0.5035316423655090 0.1730835860187696 0.1809526290281340 T T T

0.5178099999999972 0.2036700000000025 0.0010600000000025 F F F

0.6666200000000018 0.1566199999999967 0.0598400000000012 F F F

0.8227100000000007 0.9993899999999982 0.1198899999999981 F F F

0.8346970183195808 0.1723378073210640 0.1811869567168358 T T T

0.8507900000000035 0.2027799999999971 0.0008399999999966 F F F

0.9999600000000015 0.4899399999999972 0.0598299999999981 F F F

0.1560899999999990 0.3327299999999980 0.1198899999999981 F F F

0.1779498112429536 0.5090796426796018 0.1813016747807339 T T T

0.1840600000000023 0.5358400000000003 0.0007599999999996 F F F

0.3332699999999988 0.4899600000000035 0.0598499999999973 F F F

0.4894099999999995 0.3326899999999995 0.1198899999999981 F F F

0.4999537674324372 0.5066799899742094 0.1815988587370683 T T T

0.5176700000000025 0.5366500000000016 0.0009599999999992 F F F

0.6666099999999986 0.4899699999999996 0.0598400000000012 F F F

0.8227300000000000 0.3327499999999972 0.1198600000000027 F F F

0.8384303326725373 0.5067490997194142 0.1810001668751511 T T T

0.8510899999999992 0.5369499999999974 0.0010300000000001 F F F

0.9999299999999991 0.8233099999999993 0.0598499999999973 F F F

0.1561299999999974 0.6660899999999970 0.1198699999999988 F F F

0.1683608353143740 0.8390074069180997 0.1809879043228406 T T T

0.1843999999999966 0.8701200000000000 0.0009900000000016 F F F

0.3332800000000020 0.8232799999999969 0.0598400000000012 F F F

0.4893900000000002 0.6661500000000018 0.1198400000000035 F F F

0.4987507223948811 0.8372263536153568 0.1815099181949549 T T T

0.5175300000000007 0.8695999999999984 0.0008600000000030 F F F

0.6665899999999993 0.8232700000000008 0.0598499999999973 F F F

0.8227699999999984 0.6660499999999985 0.1198800000000020 F F F

0.8349542878326162 0.8394681541491309 0.1808252846009779 T T T

0.8508599999999973 0.8696600000000032 0.0008699999999990 F F F

0.0245011815770582 0.0465452628107030 0.2147661327847743 T T T

0.3118686251202405 0.1600792649126177 0.2087194562758938 T T T

0.3621452092753898 0.0435152986783791 0.2142920187041568 T T T

0.6490652207743182 0.1692004971948506 0.2111315389142732 T T T

0.6896706740676777 0.0440664824817816 0.2135648389405063 T T T

0.9807606074542576 0.1679785087349899 0.2106128393841331 T T T

0.0309475309252392 0.3841955373126140 0.2141506582709027 T T T

0.3486336864265951 0.5010439441007648 0.2107946114987200 T T T

0.6470476321646998 0.4970339125550609 0.2084788484688715 T T T

0.6972426547120847 0.3780796690362038 0.2144382378185981 T T T

0.9885398209085352 0.5064709336179476 0.2098732595864945 T T T

0.0257004814639572 0.7111676731882497 0.2138172513309868 T T T

0.3112978075225143 0.8313267313465857 0.2112497129084900 T T T

0.3552578686281933 0.7043895258610817 0.2117881815231044 T T T

0.6458982212239661 0.8363800250899800 0.2113673962040110 T T T

0.6859556737840791 0.7127438110264706 0.2091675671655651 T T T

0.9794903953860654 0.8339951370722929 0.2114447849570595 T T T

0.3692333383047526 0.4042556184364443 0.2661802389293584 T T T

0.4669126316616641 0.5841798357666192 0.4973485419770540 T T T

0.4656774088640887 0.6443918958156445 0.4462178414778606 T T T

0.5540836546177630 0.5968222770920306 0.4537442042778892 T T T

0.2714684959271025 0.4583386391053637 0.4893653003446872 T T T

0.2212903049518431 0.3809060077812489 0.4405587384244356 T T T

0.2716649076902584 0.5185782653777977 0.4379537400354465 T T T

0.4830067076870250 0.3979519419722735 0.4554341462686082 T T T

0.3460702556079767 0.3102816677807496 0.4504924133061152 T T T

0.3977413814702686 0.3863372167678705 0.4996742575608621 T T T

0.5811377078797690 0.6751519716567316 0.2849382447171038 T T T

0.6723128119273198 0.6349121725912212 0.2734437463438582 T T T

0.7824999945437980 0.7355521295341134 0.3378433044232310 T T T

0.6863103311808771 0.7659197679478922 0.3559177653489556 T T T

0.7549079657888456 0.8187087513767253 0.3050284253727469 T T T

0.7008556392925769 0.5340680716267059 0.3206283700334680 T T T

0.5866646005958458 0.4273790363400565 0.3421487736147597 T T T

0.7200112683635993 0.4991480837262543 0.4002407383670814 T T T

0.6140469479765116 0.5180395706158052 0.4150256624275670 T T T

0.7330804734876963 0.6253499327044167 0.3953626442620646 T T T

0.1994522902224389 0.2721795214591864 0.2690492693760191 T T T

0.2147744124100086 0.3909551907608915 0.2892190200554907 T T T

0.0898840469817941 0.2581596833029991 0.3371762278661663 T T T

0.2069720551868670 0.3162544024781013 0.3692831449969916 T T T

0.1626904509779408 0.1932295004996689 0.3428197826220014 T T T

0.4593525087015351 0.3310123869082560 0.2873931888756118 T T T

0.3297499302550619 0.2271001206603458 0.2815262354064600 T T T

0.3088003586149526 0.2114621023123959 0.3645632182495859 T T T

0.4338611082451749 0.3226413998216913 0.3718143722419924 T T T

0.4210516895954584 0.2065831853325193 0.3473567326236022 T T T

0.4705710685034177 0.7808164663676749 0.3041088825025184 T T T

0.5349666200232761 0.7447233415427306 0.3435201940417372 T T T

0.4822825623172449 0.8615876283143109 0.3782385031365578 T T T

0.4100014844480526 0.7354126646699086 0.4030737395227888 T T T

0.3492039635620827 0.7781400331011099 0.3637150171206542 T T T

0.2559565499391994 0.5474532252353654 0.2821580060708513 T T T

0.2843947561711531 0.6713428953030203 0.2995215876219248 T T T

0.1203635297898700 0.5326374708645432 0.3342822166909158 T T T

0.2146963248714471 0.6008615694620758 0.3766215277888350 T T T

0.1881763430180001 0.4751090596033478 0.3595216203379721 T T T

0.4274607010981981 0.5167099823945875 0.3380125447463769 T T T

0.5759316500195624 0.5608388749402451 0.3279334485352194 T T T

0.3453005510750344 0.3725339892556718 0.2985313543359993 T T T

0.4051996730516265 0.4750872968708322 0.3949551493679593 T T T

0.3864674679228628 0.6239759665069187 0.3247803800065512 T T T

0.3917846518797887 0.4745538381479406 0.4425013964762599 T T T

0.4747995637062203 0.5814554800229956 0.4611524459054195 T T T

0.2823185574550209 0.4570191249290332 0.4532975093314136 T T T

0.4054477000886294 0.3869895449458056 0.4633599535369726 T T T

0.6362567212778600 0.6550161300154684 0.3008217664692694 T T T

0.7196334593920710 0.7492446284751111 0.3264858693117603 T T T

0.6361787911535117 0.5130161944631197 0.3442813494813137 T T T

0.6785522497778587 0.5412438886470170 0.3914382455291445 T T T

0.2286323561643044 0.3261686609829439 0.2974026964929521 T T T

0.1695929999444417 0.2704222635066671 0.3392632511814497 T T T

0.3812502230318984 0.2937761229519609 0.3027327768707195 T T T

0.3862927455655978 0.2572362490257092 0.3493780981856602 T T T

0.4578863487284366 0.7346408850941160 0.3347731982389561 T T T

0.4223801799423654 0.7796158943989582 0.3721465282718270 T T T

0.2800605058246148 0.5986502406198184 0.3115340447831940 T T T

0.1965862506798715 0.5494470329280503 0.3476711592044595 T T T

Final state configuration of H-transfer 1 reaction on SiO2 substrate

1.00000000000000

14.7629999999999999 0.0000000000000000 0.0000000000000000

-7.3815000000000000 12.7851330359999995 0.0000000000000000

0.0000000000000000 0.0000000000000000 30.1555999999999997

O Si H Ta N C

72 36 57 1 4 16

Selective dynamics

Direct

0.0668239769141081 0.1283045076627403 0.2135031035323820 T T T

0.0883299999999991 0.1360000000000028 0.0383199999999988 F F F

0.0476699999999965 0.2449999999999974 0.0980100000000022 F F F

0.1904145759654341 0.2841519535310297 0.1605802667498395 T T T

0.1360000000000028 0.0883299999999991 0.1407499999999970 F F F

0.2449999999999974 0.0476699999999965 0.0810600000000008 F F F

0.2745333713761013 0.1977296099370704 0.2077511296272476 T T T

0.2856700000000032 0.1973300000000009 0.0213699999999974 F F F

0.4021898522862841 0.1180675677664880 0.2148903152828865 T T T

0.4216699999999989 0.1360000000000028 0.0383199999999988 F F F

0.3810000000000002 0.2449999999999974 0.0980100000000022 F F F

0.5207893896554694 0.2825934216642736 0.1610692617681195 T T T

0.4693299999999994 0.0883299999999991 0.1407499999999970 F F F

0.5783300000000011 0.0476699999999965 0.0810600000000008 F F F

0.6149596458178976 0.2111699811581573 0.2062093301519781 T T T

0.6189999999999998 0.1973300000000009 0.0213699999999974 F F F

0.7342650037980505 0.1223942853941722 0.2148319062514901 T T T

0.7550000000000026 0.1360000000000028 0.0383199999999988 F F F

0.7143299999999968 0.2449999999999974 0.0980100000000022 F F F

0.8543349536289853 0.2833214210612383 0.1610163744172581 T T T

0.8026699999999991 0.0883299999999991 0.1407499999999970 F F F

0.9116700000000009 0.0476699999999965 0.0810600000000008 F F F

0.9462778810374175 0.2111420456053352 0.2070359666441277 T T T

0.9523300000000035 0.1973300000000009 0.0213699999999974 F F F

0.0705896598751750 0.4574829948319987 0.2147370386478471 T T T

0.0883299999999991 0.4693299999999994 0.0383199999999988 F F F

0.0476699999999965 0.5783300000000011 0.0980100000000022 F F F

0.1887324408276854 0.6173895094078645 0.1609652996685398 T T T

0.1360000000000028 0.4216699999999989 0.1407499999999970 F F F

0.2449999999999974 0.3810000000000002 0.0810600000000008 F F F

0.2837573622449554 0.5442305180207256 0.2043402419096623 T T T

0.2856700000000032 0.5306700000000006 0.0213699999999974 F F F

0.4117453646904323 0.4648522778697384 0.2190019728201449 T T T

0.4216699999999989 0.4693299999999994 0.0383199999999988 F F F

0.3810000000000002 0.5783300000000011 0.0980100000000022 F F F

0.5221304854242490 0.6164232923735042 0.1601690620720433 T T T

0.4693299999999994 0.4216699999999989 0.1407499999999970 F F F

0.5783300000000011 0.3810000000000002 0.0810600000000008 F F F

0.6199417042508770 0.5463171716027162 0.2031799741616993 T T T

0.6189999999999998 0.5306700000000006 0.0213699999999974 F F F

0.7382921260209159 0.4553232355341663 0.2159507088289345 T T T

0.7550000000000026 0.4693299999999994 0.0383199999999988 F F F

0.7143299999999968 0.5783300000000011 0.0980100000000022 F F F

0.8547060226749892 0.6166024384597293 0.1610214712798239 T T T

0.8026699999999991 0.4216699999999989 0.1407499999999970 F F F

0.9116700000000009 0.3810000000000002 0.0810600000000008 F F F

0.9502249964929632 0.5460831531781949 0.2053953744153461 T T T

0.9523300000000035 0.5306700000000006 0.0213699999999974 F F F

0.0690017699853342 0.7890289109627560 0.2152447357211500 T T T

0.0883299999999991 0.8026699999999991 0.0383199999999988 F F F

0.0476699999999965 0.9116700000000009 0.0980100000000022 F F F

0.1880293672282463 0.9498083124951080 0.1609462222555464 T T T

0.1360000000000028 0.7550000000000026 0.1407499999999970 F F F

0.2449999999999974 0.7143299999999968 0.0810600000000008 F F F

0.2813732319028119 0.8771705906029794 0.2059308067338748 T T T

0.2856700000000032 0.8639999999999972 0.0213699999999974 F F F

0.4021145704497187 0.7891015615524140 0.2150738043343097 T T T

0.4216699999999989 0.8026699999999991 0.0383199999999988 F F F

0.3810000000000002 0.9116700000000009 0.0980100000000022 F F F

0.5213632818074387 0.9500228687497270 0.1610102301867684 T T T

0.4693299999999994 0.7550000000000026 0.1407499999999970 F F F

0.5783300000000011 0.7143299999999968 0.0810600000000008 F F F

0.6146178565777163 0.8775655048586017 0.2061370442392629 T T T

0.6189999999999998 0.8639999999999972 0.0213699999999974 F F F

0.7349164617568533 0.7881155070949148 0.2153004216981742 T T T

0.7550000000000026 0.8026699999999991 0.0383199999999988 F F F

0.7143299999999968 0.9116700000000009 0.0980100000000022 F F F

0.8539510335642504 0.9495698521011775 0.1611731195166470 T T T

0.8026699999999991 0.7550000000000026 0.1407499999999970 F F F

0.9116700000000009 0.7143299999999968 0.0810600000000008 F F F

0.9466244459551945 0.8758223520018191 0.2064332717961648 T T T

0.9523300000000035 0.8639999999999972 0.0213699999999974 F F F

0.9999499999999983 0.1566100000000006 0.0598499999999973 F F F

0.1561099999999982 0.9994299999999967 0.1198499999999996 F F F

0.1712002755451465 0.1734913386583941 0.1807594980175367 T T T

0.1842499999999987 0.2030099999999990 0.0008899999999983 F F F

0.3332800000000020 0.1566400000000030 0.0598200000000020 F F F

0.4894200000000026 0.9994200000000006 0.1198600000000027 F F F

0.5043381810132885 0.1730428931739141 0.1809851636460067 T T T

0.5178099999999972 0.2036700000000025 0.0010600000000025 F F F

0.6666200000000018 0.1566199999999967 0.0598400000000012 F F F

0.8227100000000007 0.9993899999999982 0.1198899999999981 F F F

0.8369125262368158 0.1733031842895159 0.1810472725453351 T T T

0.8507900000000035 0.2027799999999971 0.0008399999999966 F F F

0.9999600000000015 0.4899399999999972 0.0598299999999981 F F F

0.1560899999999990 0.3327299999999980 0.1198899999999981 F F F

0.1723371290719626 0.5074615783217027 0.1803486958395197 T T T

0.1840600000000023 0.5358400000000003 0.0007599999999996 F F F

0.3332699999999988 0.4899600000000035 0.0598499999999973 F F F

0.4894099999999995 0.3326899999999995 0.1198899999999981 F F F

0.5044329332863171 0.5067866268459795 0.1817325166426045 T T T

0.5176700000000025 0.5366500000000016 0.0009599999999992 F F F

0.6666099999999986 0.4899699999999996 0.0598400000000012 F F F

0.8227300000000000 0.3327499999999972 0.1198600000000027 F F F

0.8383705509117050 0.5069532427275405 0.1809430440489308 T T T

0.8510899999999992 0.5369499999999974 0.0010300000000001 F F F

0.9999299999999991 0.8233099999999993 0.0598499999999973 F F F

0.1561299999999974 0.6660899999999970 0.1198699999999988 F F F

0.1706038987472098 0.8399376862029868 0.1809421564073759 T T T

0.1843999999999966 0.8701200000000000 0.0009900000000016 F F F

0.3332800000000020 0.8232799999999969 0.0598400000000012 F F F

0.4893900000000002 0.6661500000000018 0.1198400000000035 F F F

0.5041419402480969 0.8399549830247324 0.1808843800908733 T T T

0.5175300000000007 0.8695999999999984 0.0008600000000030 F F F

0.6665899999999993 0.8232700000000008 0.0598499999999973 F F F

0.8227699999999984 0.6660499999999985 0.1198800000000020 F F F

0.8368834801724532 0.8395056868115844 0.1809926401217723 T T T

0.8508599999999973 0.8696600000000032 0.0008699999999990 F F F

0.0302090347309942 0.0522101198875475 0.2176537787585175 T T T

0.3127068804979416 0.1585337366671311 0.2087958897791040 T T T

0.3636725892232775 0.0409760287884495 0.2134245460027984 T T T

0.6503205826959970 0.1695988487897253 0.2112437195852053 T T T

0.6952177345142019 0.0453825918479112 0.2148696681822813 T T T

0.9856760627323453 0.1724446409642084 0.2100506072408947 T T T

0.0301656678332378 0.3804155216407423 0.2144316425040742 T T T

0.3222235987862803 0.5050133553393295 0.2076933915855079 T T T

0.6520928612393746 0.5030058958072772 0.2102014614945915 T T T

0.6981893054178272 0.3783170610613809 0.2150511973412463 T T T

0.9854598045176246 0.5046904673625932 0.2106924998743338 T T T

0.0299966326043233 0.7119444352096593 0.2151437322635985 T T T

0.3166811247140728 0.8356291114281902 0.2109665926366233 T T T

0.3631550093022256 0.7121088368138828 0.2145300447870611 T T T

0.6490356835586439 0.8353690057888770 0.2115223518710039 T T T

0.6951546260430064 0.7109563563407661 0.2143352162495091 T T T

0.9819652178703819 0.8343605410295893 0.2112985251580710 T T T

0.8372389336084822 0.8526664882558421 0.2939236300802222 T T T

0.6401697138394908 0.5927001500300690 0.4114757145103596 T T T

0.5368761366385897 0.4686351549228505 0.3929676917746647 T T T

0.5146377402804632 0.5759221563837471 0.3984373794487297 T T T

0.7583756991462920 0.5951459328695989 0.3489596294590314 T T T

0.7137938206533150 0.5769032854115537 0.2927909121845289 T T T

0.6566842607113230 0.4704199064134968 0.3307666098548125 T T T

0.5932367527184681 0.7250613105876624 0.3453813993704244 T T T

0.6712758681931064 0.7261820878618011 0.3002342118294337 T T T

0.7227124411453190 0.7493859915639334 0.3551715361386130 T T T

0.1811145684676632 0.3667876693216527 0.2808828283590898 T T T

0.1632071866524214 0.4760663736492958 0.2811068838433499 T T T

0.1292740728409498 0.4564274769998917 0.3630081782501904 T T T

0.1577693905318824 0.3529283749105261 0.3649179824223339 T T T

0.0469455834973189 0.3373771771418674 0.3350151473415650 T T T

0.2988578525512864 0.6395409807100805 0.2977070083867019 T T T

0.4248521901089219 0.6583411588152117 0.3085628489192516 T T T

0.3755076517732263 0.7217390916245208 0.3721456781122967 T T T

0.3705166707792600 0.6035997952496643 0.3888075660981656 T T T

0.2528062960646977 0.6055287661352864 0.3780811014480463 T T T

0.0166529992139459 0.8729564189881174 0.2956363244230147 T T T

0.9130683301413285 0.7698674242692647 0.3257370153911907 T T T

0.0748406065228266 0.8602764881484362 0.3715626292471796 T T T

0.9742953463337471 0.8792520511161683 0.3952758590390886 T T T

0.0786225781811112 0.9815042322785388 0.3649123154744913 T T T

0.8998427352774669 0.0143321982863043 0.2750798016492624 T T T

0.0235013872802057 0.0356468577543296 0.2926492984675804 T T T

0.9859540234560171 0.0776130910712354 0.3697225756197042 T T T

0.8632425182164170 0.0569063626695865 0.3506124215073498 T T T

0.9812548992918764 0.1610764419966273 0.3282285708554724 T T T

0.2285316898661449 0.1452940075296993 0.3164503618412837 T T T

0.1850043984924739 0.2362846742539446 0.3153431335223118 T T T

0.1675800159149361 0.1749571221260879 0.3914490583133272 T T T

0.2723150354771207 0.3071488552843604 0.3903127690612394 T T T

0.2995580203635413 0.2032283978126230 0.3935579136276530 T T T

0.4898161159353691 0.3316717873547645 0.2812210475290655 T T T

0.3928466390365202 0.1990324479191017 0.2813540077087708 T T T

0.5402114697036069 0.2423820903336278 0.3342205383037964 T T T

0.4187028061896001 0.1863212794738587 0.3624789604676621 T T T

0.5062192381056434 0.3238931698727683 0.3652934082487249 T T T

0.3945331406567973 0.4460274275459426 0.2863104963047789 T T T

0.3065796697199161 0.5032352129194706 0.3073854757357282 T T T

0.8919675551623243 0.8974761661076869 0.3169518019132127 T T T

0.5164630527796490 0.5321706055567716 0.3122660618519433 T T T

0.3466283581673082 0.3012002485991587 0.3077543726563050 T T T

0.6069179747567119 0.5854833782452999 0.3403523047348145 T T T

0.5728124831507060 0.5537388859337327 0.3888535984118278 T T T

0.6891237927732163 0.5552882430188291 0.3273996539023258 T T T

0.6517347075462752 0.7037621432193788 0.3351894641111670 T T T

0.1938154629926103 0.4349994173309923 0.3012463406470900 T T T

0.1287808701889688 0.3932983818926985 0.3436191178804933 T T T

0.3424138290176799 0.6125409448015660 0.3186460677224758 T T T

0.3340198041387978 0.6364307627091534 0.3672810536178446 T T T

0.9620595389746924 0.8557123233954371 0.3239787508911220 T T T

0.0259888439441696 0.8963162516318590 0.3664368143141701 T T T

0.9422488210149851 0.0075584695852058 0.3038590620594732 T T T

0.9432736120571334 0.0798752936641378 0.3402898220132116 T T T

0.2482456341624015 0.2242530027201184 0.3280088649140893 T T T

0.2469880352671652 0.2272832544712458 0.3787746447959037 T T T

0.4261380202124343 0.2705879003454044 0.3017195258848062 T T T

0.4753021781540383 0.2550626553865243 0.3435503998699119 T T T

Transition state configuration of H-transfer 1 reaction on SiO2 substrate

1.00000000000000

14.7629999999999999 0.0000000000000000 0.0000000000000000

-7.3815000000000000 12.7851330359999995 0.0000000000000000

0.0000000000000000 0.0000000000000000 30.1555999999999997

O Si H Ta N C

72 36 57 1 4 16

Selective dynamics

Direct

0.0660166139218334 0.1244539119250567 0.2143044582157881 T T T

0.0883299999999991 0.1360000000000028 0.0383199999999988 F F F

0.0476699999999965 0.2449999999999974 0.0980100000000022 F F F

0.1882909364566749 0.2831780352175658 0.1608892461807345 T T T

0.1360000000000028 0.0883299999999991 0.1407499999999970 F F F

0.2449999999999974 0.0476699999999965 0.0810600000000008 F F F

0.2756013219130113 0.1998103321070569 0.2066905381667568 T T T

0.2856700000000032 0.1973300000000009 0.0213699999999974 F F F

0.4014022890680496 0.1190443631329237 0.2150270747329573 T T T

0.4216699999999989 0.1360000000000028 0.0383199999999988 F F F

0.3810000000000002 0.2449999999999974 0.0980100000000022 F F F

0.5205698863089913 0.2826205531653511 0.1611064194225469 T T T

0.4693299999999994 0.0883299999999991 0.1407499999999970 F F F

0.5783300000000011 0.0476699999999965 0.0810600000000008 F F F

0.6140320515996756 0.2110588452113022 0.2064952594345967 T T T

0.6189999999999998 0.1973300000000009 0.0213699999999974 F F F

0.7334660198417519 0.1223268597848224 0.2146803286964785 T T T

0.7550000000000026 0.1360000000000028 0.0383199999999988 F F F

0.7143299999999968 0.2449999999999974 0.0980100000000022 F F F

0.8542363114494750 0.2832649200525486 0.1610536441816574 T T T

0.8026699999999991 0.0883299999999991 0.1407499999999970 F F F

0.9116700000000009 0.0476699999999965 0.0810600000000008 F F F

0.9457777850181008 0.2101248720265161 0.2069631504541063 T T T

0.9523300000000035 0.1973300000000009 0.0213699999999974 F F F

0.0712669730848860 0.4567307335823915 0.2156205544284960 T T T

0.0883299999999991 0.4693299999999994 0.0383199999999988 F F F

0.0476699999999965 0.5783300000000011 0.0980100000000022 F F F

0.1872264715807844 0.6166870304336621 0.1610814659989921 T T T

0.1360000000000028 0.4216699999999989 0.1407499999999970 F F F

0.2449999999999974 0.3810000000000002 0.0810600000000008 F F F

0.2845287707337872 0.5473891150561543 0.2040682153771371 T T T

0.2856700000000032 0.5306700000000006 0.0213699999999974 F F F

0.4048244814516446 0.4657433945590062 0.2170274748635373 T T T

0.4216699999999989 0.4693299999999994 0.0383199999999988 F F F

0.3810000000000002 0.5783300000000011 0.0980100000000022 F F F

0.5213467611953481 0.6154530689960498 0.1604095620852078 T T T

0.4693299999999994 0.4216699999999989 0.1407499999999970 F F F

0.5783300000000011 0.3810000000000002 0.0810600000000008 F F F

0.6135844911458952 0.5406682961110788 0.2060568908517304 T T T

0.6189999999999998 0.5306700000000006 0.0213699999999974 F F F

0.7361039399919428 0.4550855791089234 0.2154703272812649 T T T

0.7550000000000026 0.4693299999999994 0.0383199999999988 F F F

0.7143299999999968 0.5783300000000011 0.0980100000000022 F F F

0.8542102900141693 0.6163528230210955 0.1611395894154536 T T T

0.8026699999999991 0.4216699999999989 0.1407499999999970 F F F

0.9116700000000009 0.3810000000000002 0.0810600000000008 F F F

0.9483231649496545 0.5442931896082462 0.2060129248530950 T T T

0.9523300000000035 0.5306700000000006 0.0213699999999974 F F F

0.0663015847639752 0.7888522040661559 0.2145294646962032 T T T

0.0883299999999991 0.8026699999999991 0.0383199999999988 F F F

0.0476699999999965 0.9116700000000009 0.0980100000000022 F F F

0.1878230154857390 0.9497869660077075 0.1609670614866028 T T T

0.1360000000000028 0.7550000000000026 0.1407499999999970 F F F

0.2449999999999974 0.7143299999999968 0.0810600000000008 F F F

0.2788152463827321 0.8758612806514781 0.2069383679064891 T T T

0.2856700000000032 0.8639999999999972 0.0213699999999974 F F F

0.3981106746411179 0.7866524318353012 0.2144592284037260 T T T

0.4216699999999989 0.8026699999999991 0.0383199999999988 F F F

0.3810000000000002 0.9116700000000009 0.0980100000000022 F F F

0.5201056346252296 0.9494097150054456 0.1611861452619721 T T T

0.4693299999999994 0.7550000000000026 0.1407499999999970 F F F

0.5783300000000011 0.7143299999999968 0.0810600000000008 F F F

0.6105786922404687 0.8760846735169798 0.2076429307215735 T T T

0.6189999999999998 0.8639999999999972 0.0213699999999974 F F F

0.7316877626954195 0.7904269758896090 0.2134836525056280 T T T

0.7550000000000026 0.8026699999999991 0.0383199999999988 F F F

0.7143299999999968 0.9116700000000009 0.0980100000000022 F F F

0.8555066791947308 0.9505334687859121 0.1608644507588165 T T T

0.8026699999999991 0.7550000000000026 0.1407499999999970 F F F

0.9116700000000009 0.7143299999999968 0.0810600000000008 F F F

0.9438496900123824 0.8747583387154997 0.2076857039017695 T T T

0.9523300000000035 0.8639999999999972 0.0213699999999974 F F F

0.9999499999999983 0.1566100000000006 0.0598499999999973 F F F

0.1561099999999982 0.9994299999999967 0.1198499999999996 F F F

0.1697987529458947 0.1728233411935053 0.1808910665585734 T T T

0.1842499999999987 0.2030099999999990 0.0008899999999983 F F F

0.3332800000000020 0.1566400000000030 0.0598200000000020 F F F

0.4894200000000026 0.9994200000000006 0.1198600000000027 F F F

0.5035943199807554 0.1728540743852577 0.1811019787799766 T T T

0.5178099999999972 0.2036700000000025 0.0010600000000025 F F F

0.6666200000000018 0.1566199999999967 0.0598400000000012 F F F

0.8227100000000007 0.9993899999999982 0.1198899999999981 F F F

0.8364433254212271 0.1730768247743869 0.1810696115744868 T T T

0.8507900000000035 0.2027799999999971 0.0008399999999966 F F F

0.9999600000000015 0.4899399999999972 0.0598299999999981 F F F

0.1560899999999990 0.3327299999999980 0.1198899999999981 F F F

0.1720288408971796 0.5072348288725227 0.1807094172103557 T T T

0.1840600000000023 0.5358400000000003 0.0007599999999996 F F F

0.3332699999999988 0.4899600000000035 0.0598499999999973 F F F

0.4894099999999995 0.3326899999999995 0.1198899999999981 F F F

0.5025564370284368 0.5054762550308677 0.1817828945224452 T T T

0.5176700000000025 0.5366500000000016 0.0009599999999992 F F F

0.6666099999999986 0.4899699999999996 0.0598400000000012 F F F

0.8227300000000000 0.3327499999999972 0.1198600000000027 F F F

0.8374628285867598 0.5064363434035358 0.1810160902434708 T T T

0.8510899999999992 0.5369499999999974 0.0010300000000001 F F F

0.9999299999999991 0.8233099999999993 0.0598499999999973 F F F

0.1561299999999974 0.6660899999999970 0.1198699999999988 F F F

0.1694256263024868 0.8395589815845068 0.1810086293949777 T T T

0.1843999999999966 0.8701200000000000 0.0009900000000016 F F F

0.3332800000000020 0.8232799999999969 0.0598400000000012 F F F

0.4893900000000002 0.6661500000000018 0.1198400000000035 F F F

0.5017350250569954 0.8389650096870556 0.1811969785610620 T T T

0.5175300000000007 0.8695999999999984 0.0008600000000030 F F F

0.6665899999999993 0.8232700000000008 0.0598499999999973 F F F

0.8227699999999984 0.6660499999999985 0.1198800000000020 F F F

0.8361423508969982 0.8398893791045680 0.1808359283978415 T T T

0.8508599999999973 0.8696600000000032 0.0008699999999990 F F F

0.0278765927650347 0.0476519174813381 0.2158545248603251 T T T

0.3119146703864024 0.1587461177497413 0.2084197271564187 T T T

0.3633645322588246 0.0419760157069141 0.2145542966263000 T T T

0.6495046085208187 0.1696157649550762 0.2115278645705225 T T T

0.6939305888550821 0.0453272450714501 0.2148419550823587 T T T

0.9831404906874547 0.1699440454422779 0.2110116945220330 T T T

0.0312040314994686 0.3797178108914325 0.2154054130098270 T T T

0.3215404994506896 0.5075653020382613 0.2107700476952059 T T T

0.6493696455451227 0.4995209702168437 0.2103855310032439 T T T

0.6968172511325450 0.3780122986004550 0.2152678091084942 T T T

0.9838498213539854 0.5031574171620252 0.2112984877059233 T T T

0.0275136322957328 0.7118310167474604 0.2146692563008477 T T T

0.3141402752700861 0.8340732469389263 0.2114063055685487 T T T

0.3593425507296880 0.7094376250273784 0.2135316883643769 T T T

0.6484146286136897 0.8363717834871105 0.2111270729527746 T T T

0.6915756399536227 0.7135135696745877 0.2133939932813431 T T T

0.9809231039227925 0.8344936424597199 0.2115253468124030 T T T

0.3909559833179810 0.4202285731439598 0.2438789050709461 T T T

0.4805760747117618 0.5795702102540472 0.4977356405688235 T T T

0.4664573270581474 0.6357710710207749 0.4476256220504160 T T T

0.5636586887411433 0.5984809215643732 0.4514368167452926 T T T

0.2869419855962257 0.4421340220386428 0.4938367220746449 T T T

0.2349861463384735 0.3631023986498026 0.4454625450616088 T T T

0.2748370860768735 0.4985117849642465 0.4433654712718702 T T T

0.5082230096423279 0.4039265143870924 0.4534855140387375 T T T

0.3731721107288770 0.3073290694708729 0.4505896674816684 T T T

0.4244216013248274 0.3844099999086157 0.4996051241957759 T T T

0.5812654148579255 0.6773508663160229 0.2831773683030799 T T T

0.6926662676592370 0.6627202170761565 0.2798602337684690 T T T

0.7594532004033654 0.7536390654863578 0.3517423315037658 T T T

0.6407309015940718 0.7519263965665470 0.3623557608734863 T T T

0.7236567362201312 0.8296300794955073 0.3181398530185410 T T T

0.6925013678341256 0.5219375018127591 0.3096765737329337 T T T

0.5850984862420670 0.4270094617182513 0.3397451216855251 T T T

0.7291348415180523 0.4967825277252307 0.3897464540401451 T T T

0.6456989518556080 0.5446646869102282 0.4074428254010745 T T T

0.7648434636203632 0.6287777785895866 0.3790922714880737 T T T

0.2087233890033032 0.2300672997502019 0.2824655110795030 T T T

0.2066685226798865 0.3368322836529551 0.3068021712369511 T T T

0.1183357860730556 0.2010937743290943 0.3589466282827920 T T T

0.2455946587745579 0.2765633835509976 0.3824706178516522 T T T

0.2086657049636675 0.1555731374499854 0.3555804550236823 T T T

0.4855392422738717 0.3431545527778109 0.2828024459962908 T T T

0.3646623799457535 0.2287104224852843 0.2736891039607025 T T T

0.3539171496917206 0.1727203545029524 0.3512560249521475 T T T

0.4585351013630349 0.2962027841203076 0.3673158260331666 T T T

0.4816076127973757 0.2132966238141241 0.3315010874636180 T T T

0.4168953487034666 0.7322401291224561 0.3024180249215074 T T T

0.4923759549743199 0.7063797404670864 0.3398264284886154 T T T

0.4223028223213821 0.8017390737014626 0.3788279788070953 T T T

0.3602910822047538 0.6720622262615418 0.4007276779308433 T T T

0.2917546464989429 0.7104923705439771 0.3634685479918927 T T T

0.2209413939270216 0.4726170268194561 0.2832395642803135 T T T

0.2334416628674062 0.5946647102409248 0.2940684338246605 T T T

0.0852201216129007 0.4476982677585056 0.3358169526222819 T T T

0.1775495373948531 0.5407256594323041 0.3736671130020350 T T T

0.1754401207238609 0.4214267115548522 0.3642794688371332 T T T

0.4236368519699027 0.4919317999676259 0.3369969526903045 T T T

0.5766067916957887 0.5595908867555421 0.3231097133669014 T T T

0.3549932410540801 0.3510246697012312 0.3039901772926835 T T T

0.4120947894683128 0.4678164047434818 0.3959959229193267 T T T

0.3537000774804486 0.5742979266475736 0.3201943773434633 T T T

0.4044513690212163 0.4673971854227413 0.4435501030949096 T T T

0.4836670983529786 0.5769071858967599 0.4612985237467320 T T T

0.2935366950337016 0.4411198184266297 0.4574821869136147 T T T

0.4290432420779677 0.3857865490396346 0.4631424425843453 T T T

0.6355539661325487 0.6633114848044402 0.3033351358053994 T T T

0.6932177554396088 0.7547452554451852 0.3358011755135017 T T T

0.6382908807236420 0.5118478456972538 0.3371135879471127 T T T

0.6983954109507883 0.5486687879733774 0.3808290903954159 T T T

0.2405882031826435 0.2855758289344856 0.3104072442111142 T T T

0.2015932216452235 0.2260731458264887 0.3544161898162125 T T T

0.4089786997739397 0.2905130824004926 0.2981464141603948 T T T

0.4264667096289398 0.2406133471151940 0.3395500034069205 T T T

0.4110091584790607 0.6852228727464537 0.3320856321408012 T T T

0.3683752342080790 0.7188638163016535 0.3710707415016262 T T T

0.2430232316008542 0.5319695593034366 0.3094271764035822 T T T

0.1661458890745960 0.4830726590708866 0.3481003792809919 T T T

Final state configuration of H-transfer 2 reaction on SiO2 substrate

1.00000000000000

14.7629999999999999 0.0000000000000000 0.0000000000000000

-7.3815000000000000 12.7851330359999995 0.0000000000000000

0.0000000000000000 0.0000000000000000 30.1555999999999997

O Si H Ta N C

72 36 46 1 3 12

Selective dynamics

Direct

0.0666177042005671 0.1249473377282229 0.2136874708791154 T T T

0.0883299999999991 0.1360000000000028 0.0383199999999988 F F F

0.0476699999999965 0.2449999999999974 0.0980100000000022 F F F

0.1887938743344009 0.2830975994860481 0.1604972455596325 T T T

0.1360000000000028 0.0883299999999991 0.1407499999999970 F F F

0.2449999999999974 0.0476699999999965 0.0810600000000008 F F F

0.2771375953839836 0.2006664999546999 0.2062399304981781 T T T

0.2856700000000032 0.1973300000000009 0.0213699999999974 F F F

0.4032094246875104 0.1205054371316336 0.2154689136086745 T T T

0.4216699999999989 0.1360000000000028 0.0383199999999988 F F F

0.3810000000000002 0.2449999999999974 0.0980100000000022 F F F

0.5216571683195852 0.2826873495959816 0.1607594352252306 T T T

0.4693299999999994 0.0883299999999991 0.1407499999999970 F F F

0.5783300000000011 0.0476699999999965 0.0810600000000008 F F F

0.6152492702520078 0.2111819701193340 0.2059927314858285 T T T

0.6189999999999998 0.1973300000000009 0.0213699999999974 F F F

0.7347089510121592 0.1219844734991753 0.2151842591965902 T T T

0.7550000000000026 0.1360000000000028 0.0383199999999988 F F F

0.7143299999999968 0.2449999999999974 0.0980100000000022 F F F

0.8535478189719896 0.2827040396827059 0.1610679083489046 T T T

0.8026699999999991 0.0883299999999991 0.1407499999999970 F F F

0.9116700000000009 0.0476699999999965 0.0810600000000008 F F F

0.9467310317752435 0.2109997575203550 0.2064680504732266 T T T

0.9523300000000035 0.1973300000000009 0.0213699999999974 F F F

0.0543334696205306 0.4497655609443143 0.2109322707080210 T T T

0.0883299999999991 0.4693299999999994 0.0383199999999988 F F F

0.0476699999999965 0.5783300000000011 0.0980100000000022 F F F

0.1894173736022040 0.6161349649797927 0.1608293096096318 T T T

0.1360000000000028 0.4216699999999989 0.1407499999999970 F F F

0.2449999999999974 0.3810000000000002 0.0810600000000008 F F F

0.2633641499473143 0.5314584781322580 0.2142027622443905 T T T

0.2856700000000032 0.5306700000000006 0.0213699999999974 F F F

0.4109426787805788 0.4688253711035060 0.2195455385836311 T T T

0.4216699999999989 0.4693299999999994 0.0383199999999988 F F F

0.3810000000000002 0.5783300000000011 0.0980100000000022 F F F

0.5229086772146943 0.6172687621403341 0.1599828742401144 T T T

0.4693299999999994 0.4216699999999989 0.1407499999999970 F F F

0.5783300000000011 0.3810000000000002 0.0810600000000008 F F F

0.6172430937930642 0.5465011318408131 0.2038740260526026 T T T

0.6189999999999998 0.5306700000000006 0.0213699999999974 F F F

0.7340721729307873 0.4538529814633065 0.2152644471067973 T T T

0.7550000000000026 0.4693299999999994 0.0383199999999988 F F F

0.7143299999999968 0.5783300000000011 0.0980100000000022 F F F

0.8539470734116179 0.6165965556428503 0.1611270360717683 T T T

0.8026699999999991 0.4216699999999989 0.1407499999999970 F F F

0.9116700000000009 0.3810000000000002 0.0810600000000008 F F F

0.9458280744295635 0.5449618746332092 0.2068003696598808 T T T

0.9523300000000035 0.5306700000000006 0.0213699999999974 F F F

0.0651440700033490 0.7868756256297260 0.2146615636394742 T T T

0.0883299999999991 0.8026699999999991 0.0383199999999988 F F F

0.0476699999999965 0.9116700000000009 0.0980100000000022 F F F

0.1872978057587105 0.9492591950589429 0.1610550372945596 T T T

0.1360000000000028 0.7550000000000026 0.1407499999999970 F F F

0.2449999999999974 0.7143299999999968 0.0810600000000008 F F F

0.2771066212941804 0.8750228950868006 0.2076816561736052 T T T

0.2856700000000032 0.8639999999999972 0.0213699999999974 F F F

0.3969048805022695 0.7840887217135091 0.2133443722169872 T T T

0.4216699999999989 0.8026699999999991 0.0383199999999988 F F F

0.3810000000000002 0.9116700000000009 0.0980100000000022 F F F

0.5212335456404347 0.9501202319783744 0.1611883817955473 T T T

0.4693299999999994 0.7550000000000026 0.1407499999999970 F F F

0.5783300000000011 0.7143299999999968 0.0810600000000008 F F F

0.6100403993440153 0.8748230787506515 0.2080004244655029 T T T

0.6189999999999998 0.8639999999999972 0.0213699999999974 F F F

0.7314567464916877 0.7883967193063555 0.2132197457262635 T T T

0.7550000000000026 0.8026699999999991 0.0383199999999988 F F F

0.7143299999999968 0.9116700000000009 0.0980100000000022 F F F

0.8550617319740041 0.9505068687900220 0.1610202743595721 T T T

0.8026699999999991 0.7550000000000026 0.1407499999999970 F F F

0.9116700000000009 0.7143299999999968 0.0810600000000008 F F F

0.9444153993610911 0.8752943274056975 0.2074938569091103 T T T

0.9523300000000035 0.8639999999999972 0.0213699999999974 F F F

0.9999499999999983 0.1566100000000006 0.0598499999999973 F F F

0.1561099999999982 0.9994299999999967 0.1198499999999996 F F F

0.1713702761289397 0.1736538763428328 0.1807208813860584 T T T

0.1842499999999987 0.2030099999999990 0.0008899999999983 F F F

0.3332800000000020 0.1566400000000030 0.0598200000000020 F F F

0.4894200000000026 0.9994200000000006 0.1198600000000027 F F F

0.5043888912269097 0.1733396812077643 0.1810367409251938 T T T

0.5178099999999972 0.2036700000000025 0.0010600000000025 F F F

0.6666200000000018 0.1566199999999967 0.0598400000000012 F F F

0.8227100000000007 0.9993899999999982 0.1198899999999981 F F F

0.8367923936978769 0.1729776390023616 0.1810243414667028 T T T

0.8507900000000035 0.2027799999999971 0.0008399999999966 F F F

0.9999600000000015 0.4899399999999972 0.0598299999999981 F F F

0.1560899999999990 0.3327299999999980 0.1198899999999981 F F F

0.1660595218728531 0.5034808494530552 0.1821530654181984 T T T

0.1840600000000023 0.5358400000000003 0.0007599999999996 F F F

0.3332699999999988 0.4899600000000035 0.0598499999999973 F F F

0.4894099999999995 0.3326899999999995 0.1198899999999981 F F F

0.5009208321210963 0.5060174984499994 0.1823410387193931 T T T

0.5176700000000025 0.5366500000000016 0.0009599999999992 F F F

0.6666099999999986 0.4899699999999996 0.0598400000000012 F F F

0.8227300000000000 0.3327499999999972 0.1198600000000027 F F F

0.8356262233882461 0.5062439207566882 0.1811482628032337 T T T

0.8510899999999992 0.5369499999999974 0.0010300000000001 F F F

0.9999299999999991 0.8233099999999993 0.0598499999999973 F F F

0.1561299999999974 0.6660899999999970 0.1198699999999988 F F F

0.1681916990596832 0.8389897073806836 0.1811601961384142 T T T

0.1843999999999966 0.8701200000000000 0.0009900000000016 F F F

0.3332800000000020 0.8232799999999969 0.0598400000000012 F F F

0.4893900000000002 0.6661500000000018 0.1198400000000035 F F F

0.5028720727611002 0.8395517031392004 0.1806620402760473 T T T

0.5175300000000007 0.8695999999999984 0.0008600000000030 F F F

0.6665899999999993 0.8232700000000008 0.0598499999999973 F F F

0.8227699999999984 0.6660499999999985 0.1198800000000020 F F F

0.8363947639213478 0.8398567210692320 0.1807353826668034 T T T

0.8508599999999973 0.8696600000000032 0.0008699999999990 F F F

0.0286402900807659 0.0481859569670746 0.2151662459280104 T T T

0.3145920767236916 0.1611121313803068 0.2093791925661179 T T T

0.3638221247692570 0.0436265374350171 0.2148137575450306 T T T

0.6502067363301478 0.1695067375552384 0.2114250306996439 T T T

0.6951660353067055 0.0450621025784770 0.2151009045338768 T T T

0.9854183290185716 0.1719156367478405 0.2102591626746033 T T T

0.0153596830946228 0.3727205077915343 0.2092265138873017 T T T

0.2694229651855267 0.6365620534031393 0.2649233239504980 T T T

0.6488554049447970 0.5026513210207071 0.2105857670382960 T T T

0.6941937965514740 0.3768817500529877 0.2143383653264692 T T T

0.9804877056491534 0.5017729139795293 0.2101723650392842 T T T

0.0252960947071585 0.7097238960544558 0.2137966860040805 T T T

0.3142553192194839 0.8350302514356969 0.2109760334048332 T T T

0.3505151244304727 0.7111606956829206 0.2039416894259887 T T T

0.6485656015094392 0.8352645879186582 0.2102995036840341 T T T

0.6915730528838040 0.7113027763515376 0.2107788415712406 T T T

0.9801889659940315 0.8340013289166137 0.2117696923066035 T T T

0.2122440983478739 0.2499917978084554 0.3965121326501944 T T T

0.2100552062875636 0.3681428030709668 0.3881643046712568 T T T

0.3300245728355762 0.3675843067153792 0.3836820280845714 T T T

0.0731912793846874 0.1580991753988670 0.3391880377513559 T T T

0.0914731582320698 0.2062300203877498 0.2838673934188378 T T T

0.0681074537350668 0.2738022425970499 0.3274031695394584 T T T

0.3634098060634514 0.2773903895686090 0.3213970433932687 T T T

0.2691790156499181 0.2131524805195966 0.2789410896340669 T T T

0.2453337741451449 0.1590579287345193 0.3336780844319094 T T T

0.5027152157490065 0.7033444287934856 0.2594292150730055 T T T

0.6018041186327494 0.6696299667330479 0.2650679989960665 T T T

0.6597253992583063 0.7635848050687315 0.3358782425543581 T T T

0.5530144523580987 0.7873778485746925 0.3369750231994075 T T T

0.6514610202721940 0.8462077967729655 0.2954303855891709 T T T

0.5956627931086942 0.5645426204815251 0.3233359985737820 T T T

0.4713617944674553 0.4533681729700731 0.3315219209546001 T T T

0.5556692096798486 0.5312766489099445 0.4030949870502099 T T T

0.4381918052535809 0.5357004770896694 0.3969867359429315 T T T

0.5612879572255380 0.6514905472802752 0.3899220547751483 T T T

0.2861425666426385 0.7478298302765864 0.3243223362508303 T T T

0.4020052596428556 0.7538139885616033 0.3049184426742713 T T T

0.4171382527631025 0.7628315479741090 0.3839193741748304 T T T

0.3863099922715492 0.6335369095739338 0.3701081315389416 T T T

0.2889559881654478 0.6576222797147295 0.3929418819972028 T T T

0.1147609770329083 0.4958953736778184 0.2797490682119275 T T T

0.1259855804661996 0.6091062772404641 0.3052317186973568 T T T

0.0355697215812043 0.4447818398344339 0.3513432561258725 T T T

0.1439015556536560 0.5461658989332960 0.3795709624158192 T T T

0.1549358170481057 0.4428630033117784 0.3542897677434809 T T T

0.3398430256617075 0.5039188474424847 0.2674704258429230 T T T

0.4689781708698746 0.5820546919924823 0.3041275484053827 T T T

0.2703337690295839 0.3900697757074028 0.3009826131471556 T T T

0.2679417193567133 0.6083983174112149 0.2962361357701795 T T T

0.2257248054277383 0.2950447165772800 0.3270941899070436 T T T

0.2457763781878768 0.3224328314260845 0.3768140690890149 T T T

0.1072988667695753 0.2295048758165308 0.3187832845235548 T T T

0.2790479469813789 0.2320148873631993 0.3143478402787139 T T T

0.5446170086999800 0.6816249212242198 0.2839808126504764 T T T

0.6051257834970585 0.7749273214749337 0.3150697926918511 T T T

0.5151893989322218 0.5389958351044442 0.3347775317568917 T T T

0.5178643639124232 0.5667894162532292 0.3838876836926275 T T T

0.3295957026676319 0.7047596201461133 0.3235166696125447 T T T

0.3559834153045003 0.6877116865606396 0.3703601501320861 T T T

0.1532098004210454 0.5516459756216605 0.3068656514036263 T T T

0.1212923167893365 0.4939475733693399 0.3507757270802330 T T T

Transition state configuration of H-transfer 2 reaction on SiO2 substrate

1.00000000000000

14.7629999999999999 0.0000000000000000 0.0000000000000000

-7.3815000000000000 12.7851330359999995 0.0000000000000000

0.0000000000000000 0.0000000000000000 30.1555999999999997

O Si H Ta N C

72 36 46 1 3 12

Selective dynamics

Direct

0.0647210668683619 0.1245269151546867 0.2133474472783193 T T T

0.0883299999999991 0.1360000000000028 0.0383199999999988 F F F

0.0476699999999965 0.2449999999999974 0.0980100000000022 F F F

0.1886510147742202 0.2831746365567317 0.1606329276509368 T T T

0.1360000000000028 0.0883299999999991 0.1407499999999970 F F F

0.2449999999999974 0.0476699999999965 0.0810600000000008 F F F

0.2745439697023220 0.1991718594035907 0.2074140108608886 T T T

0.2856700000000032 0.1973300000000009 0.0213699999999974 F F F

0.4026288141840686 0.1207250830467722 0.2151948029843528 T T T

0.4216699999999989 0.1360000000000028 0.0383199999999988 F F F

0.3810000000000002 0.2449999999999974 0.0980100000000022 F F F

0.5215387405846690 0.2828623155701902 0.1608398437729670 T T T

0.4693299999999994 0.0883299999999991 0.1407499999999970 F F F

0.5783300000000011 0.0476699999999965 0.0810600000000008 F F F

0.6149144364889122 0.2112846420229317 0.2061666690492606 T T T

0.6189999999999998 0.1973300000000009 0.0213699999999974 F F F

0.7344527814681571 0.1224675218567261 0.2150457504631159 T T T

0.7550000000000026 0.1360000000000028 0.0383199999999988 F F F

0.7143299999999968 0.2449999999999974 0.0980100000000022 F F F

0.8539518677024204 0.2830790357063940 0.1610672207337134 T T T

0.8026699999999991 0.0883299999999991 0.1407499999999970 F F F

0.9116700000000009 0.0476699999999965 0.0810600000000008 F F F

0.9464701460226125 0.2111774898095059 0.2066984398971031 T T T

0.9523300000000035 0.1973300000000009 0.0213699999999974 F F F

0.0601803006690981 0.4498425715786227 0.2142328497990244 T T T

0.0883299999999991 0.4693299999999994 0.0383199999999988 F F F

0.0476699999999965 0.5783300000000011 0.0980100000000022 F F F

0.1855337306668453 0.6149965687940464 0.1608290901030152 T T T

0.1360000000000028 0.4216699999999989 0.1407499999999970 F F F

0.2449999999999974 0.3810000000000002 0.0810600000000008 F F F

0.2724620908156502 0.5425701305771251 0.2090103235311524 T T T

0.2856700000000032 0.5306700000000006 0.0213699999999974 F F F

0.4210251465633573 0.4683085431399461 0.2210830021011817 T T T

0.4216699999999989 0.4693299999999994 0.0383199999999988 F F F

0.3810000000000002 0.5783300000000011 0.0980100000000022 F F F

0.5230166489929469 0.6171364506157523 0.1600308322536737 T T T

0.4693299999999994 0.4216699999999989 0.1407499999999970 F F F

0.5783300000000011 0.3810000000000002 0.0810600000000008 F F F

0.6247658825667912 0.5501856751458246 0.2009656754143640 T T T

0.6189999999999998 0.5306700000000006 0.0213699999999974 F F F

0.7368247366864284 0.4548305834654343 0.2158512026833078 T T T

0.7550000000000026 0.4693299999999994 0.0383199999999988 F F F

0.7143299999999968 0.5783300000000011 0.0980100000000022 F F F

0.8547051439843840 0.6165541828370895 0.1609897522152719 T T T

0.8026699999999991 0.4216699999999989 0.1407499999999970 F F F

0.9116700000000009 0.3810000000000002 0.0810600000000008 F F F

0.9482230531261582 0.5450737576788655 0.2060351439545658 T T T

0.9523300000000035 0.5306700000000006 0.0213699999999974 F F F

0.0649989047865357 0.7880684389690203 0.2143421943169460 T T T

0.0883299999999991 0.8026699999999991 0.0383199999999988 F F F

0.0476699999999965 0.9116700000000009 0.0980100000000022 F F F

0.1874421643492439 0.9496302529614556 0.1609670301479222 T T T

0.1360000000000028 0.7550000000000026 0.1407499999999970 F F F

0.2449999999999974 0.7143299999999968 0.0810600000000008 F F F

0.2772706255131214 0.8759341295526269 0.2075737807414340 T T T

0.2856700000000032 0.8639999999999972 0.0213699999999974 F F F

0.3951273048693349 0.7874330004026269 0.2125523755807840 T T T

0.4216699999999989 0.8026699999999991 0.0383199999999988 F F F

0.3810000000000002 0.9116700000000009 0.0980100000000022 F F F

0.5223878518592884 0.9504280140506198 0.1609182287367398 T T T

0.4693299999999994 0.7550000000000026 0.1407499999999970 F F F

0.5783300000000011 0.7143299999999968 0.0810600000000008 F F F

0.6068668391275570 0.8717309456877780 0.2092388015847206 T T T

0.6189999999999998 0.8639999999999972 0.0213699999999974 F F F

0.7301253298760197 0.7869290003845887 0.2134087679069248 T T T

0.7550000000000026 0.8026699999999991 0.0383199999999988 F F F

0.7143299999999968 0.9116700000000009 0.0980100000000022 F F F

0.8540697869149975 0.9499723635982887 0.1611673625278674 T T T

0.8026699999999991 0.7550000000000026 0.1407499999999970 F F F

0.9116700000000009 0.7143299999999968 0.0810600000000008 F F F

0.9431904295309466 0.8749937219496179 0.2078243799899843 T T T

0.9523300000000035 0.8639999999999972 0.0213699999999974 F F F

0.9999499999999983 0.1566100000000006 0.0598499999999973 F F F

0.1561099999999982 0.9994299999999967 0.1198499999999996 F F F

0.1703133340925293 0.1731882405811258 0.1808426549768369 T T T

0.1842499999999987 0.2030099999999990 0.0008899999999983 F F F

0.3332800000000020 0.1566400000000030 0.0598200000000020 F F F

0.4894200000000026 0.9994200000000006 0.1198600000000027 F F F

0.5042019736254602 0.1733167625206145 0.1810134946348616 T T T

0.5178099999999972 0.2036700000000025 0.0010600000000025 F F F

0.6666200000000018 0.1566199999999967 0.0598400000000012 F F F

0.8227100000000007 0.9993899999999982 0.1198899999999981 F F F

0.8366853892945999 0.1731029263641316 0.1810056617265810 T T T

0.8507900000000035 0.2027799999999971 0.0008399999999966 F F F

0.9999600000000015 0.4899399999999972 0.0598299999999981 F F F

0.1560899999999990 0.3327299999999980 0.1198899999999981 F F F

0.1648472158754136 0.5042727620779348 0.1816134873713236 T T T

0.1840600000000023 0.5358400000000003 0.0007599999999996 F F F

0.3332699999999988 0.4899600000000035 0.0598499999999973 F F F

0.4894099999999995 0.3326899999999995 0.1198899999999981 F F F

0.5063196625125183 0.5077149081728405 0.1815632202707196 T T T

0.5176700000000025 0.5366500000000016 0.0009599999999992 F F F

0.6666099999999986 0.4899699999999996 0.0598400000000012 F F F

0.8227300000000000 0.3327499999999972 0.1198600000000027 F F F

0.8372619139386226 0.5065741633532198 0.1810046175780155 T T T

0.8510899999999992 0.5369499999999974 0.0010300000000001 F F F

0.9999299999999991 0.8233099999999993 0.0598499999999973 F F F

0.1561299999999974 0.6660899999999970 0.1198699999999988 F F F

0.1685607201528291 0.8394017721883158 0.1810756767704433 T T T

0.1843999999999966 0.8701200000000000 0.0009900000000016 F F F

0.3332800000000020 0.8232799999999969 0.0598400000000012 F F F

0.4893900000000002 0.6661500000000018 0.1198400000000035 F F F

0.5014937463129842 0.8391091171654681 0.1808317512957286 T T T

0.5175300000000007 0.8695999999999984 0.0008600000000030 F F F

0.6665899999999993 0.8232700000000008 0.0598499999999973 F F F

0.8227699999999984 0.6660499999999985 0.1198800000000020 F F F

0.8352505582663716 0.8392389264530954 0.1809585778882332 T T T

0.8508599999999973 0.8696600000000032 0.0008699999999990 F F F

0.0267737743553875 0.0477358936879316 0.2148999156685245 T T T

0.3137428277301032 0.1609886335423107 0.2088110780830792 T T T

0.3635346978643426 0.0437105322982090 0.2148996574323362 T T T

0.6499935894958297 0.1696548709578857 0.2114055276892633 T T T

0.6948380862974051 0.0455948519640556 0.2153347937349306 T T T

0.9846858410365689 0.1714957848282160 0.2104884972787727 T T T

0.0203201465631131 0.3727163392772156 0.2124288587617225 T T T

0.2779422245624311 0.5430528139678183 0.2428484782757824 T T T

0.6546482647340783 0.5056171538413524 0.2093765032990574 T T T

0.6962263593708758 0.3778527624317934 0.2149280088019341 T T T

0.9811461707917235 0.5013994983057525 0.2115305572419022 T T T

0.0259508150605262 0.7110032748175777 0.2139894841885417 T T T

0.3147829834865930 0.8358402109786546 0.2107631166604903 T T T

0.3522555534133726 0.7109686245820369 0.2088618254811552 T T T

0.6463786133432771 0.8331916002816550 0.2108362808104257 T T T

0.6910364912308857 0.7098038845476833 0.2108822560253609 T T T

0.9801902207224557 0.8346413353619541 0.2116119067201376 T T T

0.2400280197431313 0.1972907347485079 0.4004738463577850 T T T

0.2456517830156024 0.3202638303688888 0.3959418461117964 T T T

0.3625290746178159 0.3136196135389276 0.3918752932624968 T T T

0.1039869475931707 0.1275688863169105 0.3391428366973897 T T T

0.1334704116102378 0.1894451884930208 0.2861826536879639 T T T

0.1136503869693500 0.2521756402395409 0.3327080480619189 T T T

0.3938470989571269 0.2282603428767764 0.3249484877669583 T T T

0.3000998460889505 0.1778922818538059 0.2813217063566902 T T T

0.2707417019181477 0.1129646774765752 0.3338507614751111 T T T

0.5674514413834190 0.6847024131722333 0.2836188148015669 T T T

0.6709686741489587 0.6579335652310376 0.2836889269098719 T T T

0.7050089124988145 0.7010799870406289 0.3630405987075538 T T T

0.5863232496253233 0.7028893237669335 0.3693285785792615 T T T

0.6907220283784373 0.8015020516403247 0.3373962347469474 T T T

0.6534510553524291 0.5080805174555482 0.3112884796196929 T T T

0.5270447001039331 0.3974998905300220 0.3142314489976267 T T T

0.6063468009482095 0.4118362815558272 0.3857099257089553 T T T

0.5098919126134777 0.4494477412096282 0.3931844966762908 T T T

0.6446659773898508 0.5463253643873635 0.3916023998600194 T T T

0.3257461641902868 0.7078870176268963 0.2911300778187346 T T T

0.4422502900557366 0.7051352848333267 0.2849210509256158 T T T

0.4628438775951683 0.7916081416385448 0.3537686509683607 T T T

0.4271251359565169 0.6621292765518803 0.3705401107314729 T T T

0.3336013228069550 0.7043407084134389 0.3717725239369198 T T T

0.1534323517094730 0.4380529707534834 0.2971460496101272 T T T

0.1725345506787879 0.5633665529311792 0.3091234241564990 T T T

0.1056568512075150 0.4375116369964053 0.3736669757814538 T T T

0.2258256183649505 0.5511421497125629 0.3870450175950019 T T T

0.2241648768915177 0.4333132687152054 0.3731706274970751 T T T

0.3914827691593573 0.4804278195398726 0.2841746545426638 T T T

0.5309448737834156 0.5403953999099091 0.3118927720684518 T T T

0.3176527149426645 0.3575474071954451 0.3112133934141856 T T T

0.3104785632159543 0.5581864236550451 0.2981834394582366 T T T

0.2638141012711716 0.2571154437461721 0.3329278942276148 T T T

0.2790278672411413 0.2733468731826325 0.3834109207868490 T T T

0.1465159182288928 0.2033429632201804 0.3219387024445688 T T T

0.3098468768051934 0.1895670867946170 0.3172194751305802 T T T

0.6057225324478603 0.6511176845045230 0.3040983836844089 T T T

0.6493503771215370 0.7178787510210539 0.3459702924500541 T T T

0.5760266829437100 0.4772003169550406 0.3272334008354428 T T T

0.5852291666293200 0.4715130017514350 0.3774101297434970 T T T

0.3703148416595184 0.6725816073099083 0.3049982083614923 T T T

0.3997939530742396 0.7093838301336773 0.3530919127239617 T T T

0.2028443498517773 0.5097381021815165 0.3161977101393590 T T T

0.1895096919273991 0.4821238913916595 0.3654795587843550 T T T
